# An insurmountable obstacle: Experiences of Chinese women undergoing in vitro fertilization

**DOI:** 10.1371/journal.pone.0311660

**Published:** 2024-10-07

**Authors:** Xunxun Ying, Yunxian Zhou, Yang Jin, Danhong Wu, Lingling Kong, Pingpei Dong, Xiuling Xu

**Affiliations:** 1 Hangzhou Hospital of Traditional Chinese Medicine Affiliated to Zhejiang Chinese Medical University, Hangzhou, China; 2 School of Nursing, Zhejiang Chinese Medical University, Hangzhou, China; Kasr Alainy Medical School, Cairo University, EGYPT

## Abstract

**Objective:**

This study aimed to explore the perceptions of women who have undergone unsuccessful in vitro fertilization (IVF) in Hangzhou, Zhejiang Province, China, and to explore how treatment failure has impacted their lives and relationships, thereby enabling the public to understand the unique experiences of these women.

**Design:**

A descriptive qualitative approach was employed, with purposive sampling used to recruit participants from the gynecological department of a traditional Chinese medicine clinic at a large tertiary hospital in Zhejiang province. Twelve women undergoing IVF treatment was involved in the study. Data were collected through face-to-face semi-structured interviews, which were transcribed verbatim. Conventional content analysis methods were used to analyze the data.

**Results:**

Following analysis, three main categories emerged: 1) The Psychological Experience of Initial Miscarriage from IVF Treatment, 2) The Psychological Experience of Repeated Treatment Failure, and 3) Interpersonal experiences and challenges. Women experiencing initial failure from IVF treatment reported emotions such as surprise, doubt, sadness, disappointment, and embarrassment. They perceived the process as harder than expected, leading to unexpected daily life challenges and difficulties in achieving success. As unsuccessful attempts persisted, they endured psychological suffering and lived in the shadow of repeated treatment failures. Doubts, perplexity, and anxiety grew, making reproduction seem like an insurmountable obstacle in their lives. In their interpersonal experience, women often felt guilt toward family, faced social isolation, and found it challenging to discuss IVF openly everywhere. They encountered a lack of understanding from others but also found mutual sympathy and support among people who shared similar experiences.

**Conclusions:**

The study provided an insight into the suffering of Chinese women undergoing IVF treatment, highlighting the challenges of overcoming treatment failures. The findings not only inform Chinese infertile women considering IVF treatment but also contribute to the development of more effective support services by healthcare providers.

## 1. Introduction

Infertility is a disease characterized by the failure to achieve a successful pregnancy after 12 months or longer of regular, unprotected sexual intercourse or due to an impairment in one’s ability to reproduce either as an individual or with one’s partner [1]. Infertility is estimated to affect 8%–12% of reproductive-age couples worldwide [2]. To fulfill their desire for parenthood, many infertile couples choose in vitro fertilization (IVF), a sequence of procedures that involves the extracorporeal fertilization of gametes, including conventional in vitro insemination and intracytoplasmic sperm injection (ICSI), which together constitute the majority of assisted reproductive technologies (ARTs) [1].

IVF brings hope to couples who are unable to conceive through conventional infertility treatments. However, it involves numerous invasive and exhausting testing procedures and may not always result in success [3]. The global success rate of IVF, measured by combined delivery rates, is 19.9% per cycle [4], illustrating both the hope and challenges that couples face in their journey to parenthood. Consequently, couples may need to undergo multiple IVF treatments to achieve their goal. Repeated treatment can put a strain on couples not only financially but also on their relationship, lifestyle, and physical and emotional wellbeing [5–7]. Anxiety and depression are the most common negative psychological outcomes resulting from IVF treatment failure [8–11]. Research indicates that depression and anxiety levels in women increase immediately after treatment failure, persisting above baseline measures even six months later [11, 12]. As treatment time is prolonged, these symptoms may become exacerbated [13, 14], affecting their quality of life, sex life, and marital relationship [8, 15, 16].

How do these feelings arise, and what are the influencing factors? It’s valuable to explore these questions through women’s experiences in order to develop personalized support for them. Previous studies have extensively examined women undergoing IVF treatment, focusing on their interpersonal relationships, psychological needs, and decision-making processes. These qualitative studies highlight common experiences such as feelings of loss, frustration, inadequacy, hopelessness, isolation, and guilt [17–21]. However, less attention has been paid to women’s experiences after treatment failure, especially in China, a country with a long-standing cultural history tied to childbirth [22]. To address this gap, the present study explored the experiences of Chinese women following unsuccessful IVF treatment, focusing on the psychological and emotional ramifications and their subsequent impact on relationships, aiming to capture the intricate challenges these women encounter after treatment failure. A qualitative approach is necessary in the absence of prior empirical research, as it offers a descriptive account that can serve as a foundation for future studies. By fostering an in-depth understanding of emotional and psychological complexities, qualitative methods provide rich, nuanced insights into the unique experiences of women. Through personal narratives, this approach captures the depth and variability of their responses to treatment failure [23]. This investigation aims to help healthcare providers develop tailored interventions, offering psychosocial support and counseling to promote women’s health and well-being during treatment.

## 2. Materials and methods

### 2.1. Study design

We used a descriptive qualitative approach to investigate the perspectives of Chinese infertile women based on their subjective experiences of failed IVF treatment. This approach follows the framework proposed by Lincoln and Guba and aims to provide a comprehensive summary and straightforward description of these experiences [24, 25]. It is particularly useful for personal experiences and insights that can inform and guide medical practice or policy development [26].

### 2.2. Sampling and recruitment

A purposive sampling method, combined with a maximum variation strategy, was employed to select participants who provided rich, relevant information about the research phenomenon [27]. Maximum variation in age, occupation, duration of infertility and treatment, number of miscarriages, and time since the last miscarriage was used to select patients who experienced IVF treatment failure. Inclusion criteria included: (i) Women aged between 20 and 50 years who have experienced one or more miscarriages after IVF treatment; (ii) no history of mental illness, with normal communication ability, and voluntary participation; and (iii) Chinese citizens born and living in China. Purposive sampling continued until thematic saturation was reached during data analysis. Participants were recruited via outpatient and inpatient departments at a large tertiary hospital in Hangzhou, the provincial capital of Zhejiang Province, southeast China, between December 2020 and January 2022.

### 2.3. Data collection

Data were collected using face-to-face semi-structured interviews. The interviews were conducted by the researcher in a private room at the clinic, scheduled at a convenient time for the participants. No one else was present during the interviews, which lasted between 30 and 90 minutes, depending on the participants’ preferences. The interview guide generally included the following questions: (1) How has IVF treatment affected your daily life? (2) What are your feelings after experiencing a miscarriage through IVF treatment? For women who underwent multiple failures, questions were asked about how the treatment process or repeated failures have impacted their lives. (3) How have your interpersonal relationships been affected during the treatment? (4) How do you envision your life in the future? Questions were adjusted based on participant responses. Field notes were taken during and after the interviews.

### 2.4. Data analysis

All interviews were conducted in Chinese, audio-recorded, and transcribed verbatim within one week. The data were analyzed using the conventional content analysis approach [28], which involves deriving codes directly from the data without preconceived categories. Initially, two researchers independently read each interview transcript multiple times to immerse themselves in the content and gain an overall understanding. Next, line-by-line coding was performed to identify narrative data related to experiences of IVF treatment failure. Codes and concepts generated from this process were then grouped into subthemes and themes based on observed similarities and differences. Definitions were developed for each theme and subtheme, and supporting quotes from the data were selected to illustrate these themes. Data collection and analysis occurred simultaneously, with ongoing revisions until data saturation was reached. In total, over 800 initial codes were extracted from the intervews and categorized into 9 themes and 13 subthemes.

### 2.5. Rigor and trustworthiness

For rigor, four criteria were used to establish trustworthiness in this study: credibility, dependability, confirmability, and transferability [24]. As a clinical nurse, the researcher built a trusting relationship with participants through daily nursing interactions, ensuring authenticity. Credibility was enhanced through peer debriefing, with two researchers collaborating closely and resolving disagreements with guidance from a senior qualitative researcher. The researcher documented assumptions and perceptions before data collection and used reflexive strategies, reflecting on how her role as both a mother and clinician might influence the interviews, thereby maintaining objectivity. Dependability was ensured through consistent documentation of the research process, coding, and decision-making. Confirmability was reinforced by an audit trail, ensuring the findings were based on participants’ experiences rather than researcher bias. Transferability was supported by providing rich descriptions of participants’ contexts, allowing other researchers to assess the applicability of the findings in different settings. Additionally, member checking was conducted with three participants to confirm that the subthemes and themes accurately reflected their experiences.

### 2.6. Ethical considerations

All participants were informed about the research content, expressed their willingness to participate, and signed informed consent forms before the interviews. To maintain participant anonymity, each participant was assigned a code, and a separate list linking codes to identities was stored in a secure document on the researcher’s password-protected computer. Only the researcher had access to the passwords. Ethics approval for this study was obtained from the Scientific Research Ethics Committee of Hangzhou Hospital of Traditional Chinese Medicine Affiliated with Zhejiang Chinese Medical University (approval document No. 2020KY090).

## 3. Results

A total of 12 women experiencing failed IVF treatments, aged between 29 and 40 years, were recruited via inpatient and outpatient departments. Among the participants, three are housewives, six are ordinary workers, and three are self-employed entrepreneurs. The average duration of infertility was 4.91 years, with a mean of 2.67 cycles of treatment. All the women were married. Almost all remain childless, with the exception of one participant who has a daughter from a previous marriage. Table 1 show the demographic and clinical characteristics of the study participants (n = 12).

**Table 1:** Participants’ characteristics (n = 12).

Characteristics of the participants (n = 12)							
No.	Age	Education level	Occupation	Infertility duration (Years)	Cycles of treatment	Numbers of Miscarriages	Duration from last time treatment failure (Months)
1	39	Secondary or less	Housewife	2	2	1	6
2	30	Diploma degree	Office director	3	2	1	4
3	37	Bachelor’s degree	Office worker	9	5	2	1
4	38	Bachelor’s degree	Self-employed entrepreneur	6	3	2	8
5	40	Bachelor’s degree	Office worker	4	3	1	1
6	32	Bachelor’s degree	Self-employed entrepreneur	5	3	2	5
7	37	Diploma degree	Office worker	3	2	1	7
8	40	Diploma degree	Sales manager	10	2	1	3
9	29	Secondary or less	Housewife	4	2	1	1
10	33	Diploma degree	Office worker	6	1	3	1
11	37	Bachelor’s degree	Self-employed entrepreneur	4	4	3	2
12	40	Diploma degree	Housewife	3	3	2	2

**Fig 1:**
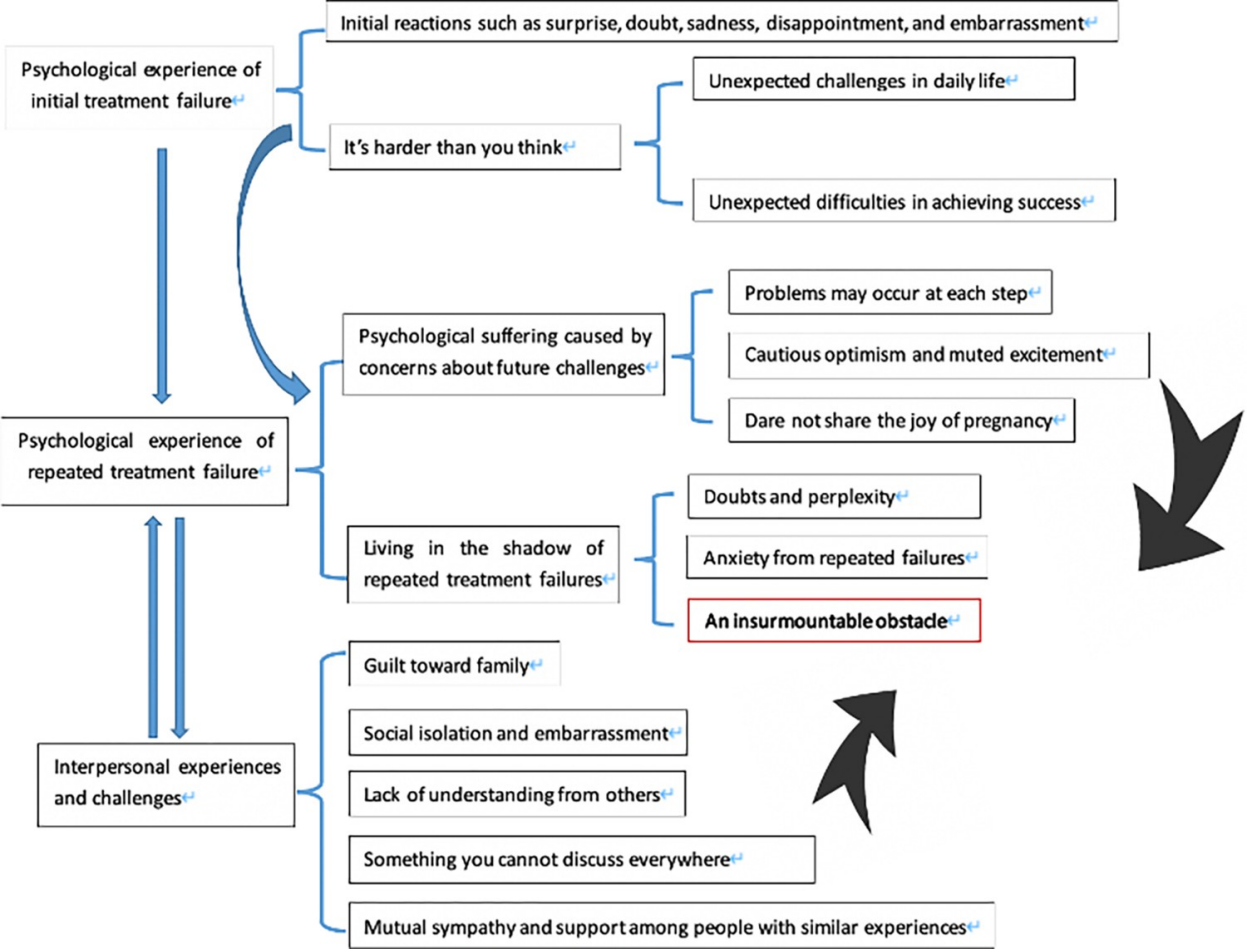
The overall experiences of Chinese women undergoing failed in vitro fertilization treatment.

The analysis revealed three main categories: 1. The Psychological Experience of Initial Miscarriage from IVF Treatment. 2. The Psychological Experience of Repeated Treatment Failure. 3. Interpersonal experiences and challenges. The core category of this study was “An insurmountable obstacle.” It doesn’t mean women had this perception immediately after a treatment failure. However, many women felt frustrated after experiencing initial failures, leading to a series of psychological reactions such as surprise, doubt, sadness, disappointment, and embarrassment. They perceived the process as harder than expected, leading to unexpected daily life challenges and unexpected difficulties in achieving success. As unsuccessful attempts persisted, they endured psychological suffering and lived in the shadow of repeated treatment failures. Doubts, perplexity, and anxiety grew, making reproduction seem like an insurmountable obstacle in their lives. With no other goals, they found themselves spending all their time undergoing treatments without any progress. The overall experiences of Chinese women undergoing failed in vitro fertilization treatment are outlined in Fig 1.

### 3.1. Psychological experience of initial treatment failure

#### 3.1.1. The initial psychological reactions

This section examines the immediate psychological reactions of individuals following the initial miscarriage subsequent to their IVF treatment. It can be categorized into five subthemes: surprise and overwhelm, doubt and denial, sadness and pain, disappointment and self-blame, embarrassment and loss of face.

*3*.*1*.*1*.*1*. *Surprise and overwhelm*. Due to heightened expectations or unfamiliarity with the IVF treatment process, five participants found themselves surprised and shocked by the abrupt fetal arrest, unsure of how to navigate through it.

One participant expressed her immediate reaction to the miscarriage:

I couldn’t fathom how something like this could happen. I kept thinking about my severe morning sickness, considering enduring it for another three or four weeks. Why did it suddenly take a turn for the worse? (P1)

Another participant shared that both she and her husband were perplexed when informed about the miscarriage:

Following the ultrasound indication of fetal arrest, the doctor directly asked whether we preferred medical or surgical abortion. I was immediately shocked because I had never considered this possibility before. I asked my husband, and he was equally shocked. (P6)

*3*.*1*.*1*.*2*. *Doubt and denial*. This subtheme encapsulates the participants’ initial feeling of doubt and confusion upon learning of the miscarriage, often resisting acceptance until re-examination confirmed the reality.

One participant conveyed her initial disbelief:

I thought it was impossible at that moment. I had never known that a miscarriage could occur so easily following IVF treatment, particularly when all the items appeared to have been thoroughly screened. (P1)

Another participant described her confusion and disbelief upon hearing about the miscarriage:

It was growing normally, and there was no indication of any issues. Suddenly, there was no heartbeat. Initially, I couldn’t wrap my head around it, wondering if it was real or not. I opted for another ultrasound to confirm. (P5)

Yet another participant disclosed continuing to take traditional Chinese medicine for pregnancy protection even after the miscarriage was confirmed:

The doctor asked us whether we wanted medical or surgical abortion. We were shocked. My husband suggested going home first, so we did. Upon returning home, my mother suggested trying traditional Chinese medicine for pregnancy protection. I took it for another two or three days, and then started bleeding. (P6)

*3*.*1*.*1*.*3*. *Sadness and* ***p****ain*. All participants experienced different degrees of sadness and sorrow following the miscarriage. The depth of emotional pain varied depending on individual circumstances and psychological resilience.

For instance, one participant who had difficulty conceiving a second child acknowledged experiencing profound sadness:

As a mother, losing a baby is undeniably heartbreaking. Regardless of how long the baby was in my belly or the strength of my emotional connection, enduring this hardship makes me feel the loss. (P2)

Another participant, reflecting on a past miscarriage through IVF, expressed lingering pain:

At that time, I would often cry in bed at night. While I try not to remember it now, recalling those moments still brings pain and distressing memories. (P7)

Yet another participant shared a similar sentiment:

I felt my whole world collapse. After all, I’m not getting any younger, and getting pregnant was a significant achievement. The emotional state was quite dire, and I frequently woke up crying in the middle of the night. (P12)

*3*.*1*.*1*.*4*. *Disappointment and self-blame*. Four participants experienced a sense of disappointment due to failed fertility aspirations, attributing part of the responsibility to themselves.

One participant expressed her feelings:

It provided hope, and then it vanished suddenly. Following that, I felt rather useless. (P5)

Another participant blamed herself regard to her extended family when talking about her experience of miscarriage, believing that she should have made more efforts to find a doctor for further treatment:

I felt that I did not try hard enough to protect the baby, and I should have gone to various hospitals to find doctors and consult with them. If I had consulted more doctors, the situation might have been different. (P12)

*3*.*1*.*1*.*5*. *Embarrassment and loss of face*. Embarrassment signifies an awkward or disconcerting feeling resulting from unforeseen circumstances, while loss of face denotes a decline in self-esteem and dignity. Three participants mentioned feelings of embarrassment and loss of face.

One participant experienced embarrassment upon discovering the miscarriage during a hospital examination with other patients:

I felt quite embarrassed at that moment because so many people heard about it. There would definitely be discussions behind my back. They might say, ’ Why did she miscarry?’ So, at that time, I felt quite embarrassed. (P1)

Another participant, hailing from a rural area where many relatives were aware of her treatment, felt humiliated after the miscarriage:

Now, even relatives knew about it (referring to the IVF treatment), and the miscarriage made me feel embarrassed." (P5)

#### 3.1.2. It’s harder than you think

Many participants underestimate the complex nature of the IVF treatment process, often assuming it involves only a few simple steps such as egg extraction, embryo matching, and transplantation. In reality, IVF is a complex and multistep process that involves various medical procedures, laboratory techniques, and careful monitoring to achieve success. This misconception can lead to unexpected challenges in daily life, and unexpected difficulties in achieving success.

*3*.*1*.*2*.*1*. *Unexpected challenges in daily life*. The numerous medical examinations, treatments, and frequent visits to the hospital that associated with IVF treatment can be overwhelming and impact daily life. Participants often find the process tedious and challenging, and some even face the difficult decision of whether or not to take time off from work.

One participant reflected on the inconvenience of requesting leave, stating:

Everyone thought that the process of IVF treatment involves only the extraction of eggs and sperm followed by implantation, which appears to be very simple. It was not until I went through it that I realized all the steps were not simple. It took me four months from the initial physical examination to the first egg collection. When the eggs were collected, the match was not good, so we had to make an additional attempt. After two rounds of egg collection, I only produced three embryos. It was too tedious. I had to consider whether or not to go to work. It’s not easy to take leave from work. (P1)

Some participants had to give up their jobs because of the medical treatments. One participant expressed regret for leaving her job, saying:

There were so many examinations, such as hysteroscopy and tests of the fallopian tubes. You have to go to the hospital all the time. I have not been to work for almost a year, even though I have a good job.” (P5)

*3*.*1*.*2*.*2*. *Unexpected difficulties in achieving success*. Many participants undergoing IVF treatment held a high expectations for the likelihood of success. They believed that pregnancy through IVF treatment would be less likely to result in a miscarriage because the embryo was screened.

One participant described her optimism during the pregnancy after embryo transplantation, stating:

My uterine environment was favorable, and I had never experienced a miscarriage. My endometrium was also in good condition. After pregnancy, I was in a good mood and did not expect that what might happen next would be so difficult. (P1)

However, despite the thorough screening process, she later realized that a pregnancy achieved through IVF treatment could easily end in a miscarriage.

Another participant had a similar misconception that embryos produced through IVF treatment would be different from naturally conceived ones. After experiencing failure, she expressed disappointment and acknowledged the difficulty of IVF treatment, saying:

I used to think that the embryos screened during the treatment would be different from naturally conceived ones. I never imagined that IVF treatment would be so challenging. Once you begin the treatment, you may suffer a lot. (P10)

### 3.2. Psychological experience of repeated treatment failures

#### 3.2.1. Psychological suffering caused by concerns about future challenges

Participants who have experienced miscarriage after IVF treatment often grapple with heightened uncertainty about the future. They describe the journey as breaking through barriers, with the possibility of encountering problems at each step, filled with psychological suffering due to concerns about future challenges. It encompasses three themes as follows:

*3*.*2*.*1*.*1*. *Problems may occur at each step*. One of the participants, who experienced the treatment for five cycles, reported the following:

We were filled with hope when we initially retrieved eggs and obtained three excellent embryos for the IVF treatment. However, both treatment attempts failed. Subsequently, in the next egg retrieval, only three eggs were retrieved, and among them, only one was deemed suitable for transplantation. Throughout the treatment, various problems and challenges emerged, making the psychological journey profoundly challenging. (P3)

Another participant who underwent IVF treatment four times described the process as being increasingly difficult:

It’s really not that simple. Problems may occur at every step. I thought if I followed the prescribed steps, I would succeed. However, it kept getting increasingly difficult. (P11)

*3*.*2*.*1*.*2 Cautious optimism and muted excitement*. For women undergoing IVF treatment, achieving a successful pregnancy following treatment can be a significant milestone. However, the process of attaining pregnancy is often prolonged and challenging. Participants described their emotions upon becoming pregnant as cautious optimism and muted excitement due to the potential for complications and disappointment.

For example, one participant said the following when she got pregnant again:

Anyway, I told my mother there are so many people who have passed the first ultrasound but failed the second, and those who have passed the second may not go through nuchal translucency scans … I do not have any special feeling that I will be very pleased to be pregnant. (P6)

However, at the end of our interview, she added:

I still have expectations, but I do not dare to be too excited. Sometimes, I feel conflicted about engaging in activities such as browsing for baby products online. I am afraid that something bad will happen later. The feelings are quite contradictory, and one has expectations, but one does not dare to expect too much. (P6)

Similarly, another participant mentioned:

Since achieving pregnancy, my husband and I had not been excited. It felt like a very long process. We were still concerned about the potential for complications and whether I would be able to give birth after such a long period. (P3)

*3*.*2*.*1*.*3*. *Dare not share the joy of pregnancy*. This feeling arises specifically from the apprehension about potential complications during pregnancy. Women undergoing IVF treatment are often hesitant to inform those around them about the process—even if they become pregnant—due to the fear that others will also be disappointed if the treatment fails. Here are statements from two participants reflecting this predicament:

I did not tell many people about my pregnancy. My mother knew, but we did not talk about it with our relatives because I had gone through a failed treatment before and I was afraid that something might go wrong again. (P6)

Actually, this time I went through IVF, I did not tell many people around me, even after I conceived. I did not tell anyone except my boss, who had to know because of work. I try not to say anything because I’m afraid of getting excited for nothing. (P10)

#### 3.2.2. Living in the shadow of repeated treatment failures

Women who have experienced repeated treatment failures often cannot understand why they are always unsuccessful. Their lives are consumed by infertility and treatment, leaving them perplexed, in a state of persistent anxiety, and feeling deeply that they have encountered an insurmountable obstacle.

*3*.*2*.*2*.*1*. *Confusion and perplexity*. Many women feel a deep sense of confusion and perplexity. They struggle to comprehend why they are repeatedly unsuccessful despite their best efforts. This ongoing confusion compounds their emotional distress, making it difficult to understand and accept their experiences.

For example, one participant noted:

Sometimes, it makes me truly perplexed. Apart from having a thin endometrium, I don’t have any other issues. Why have I attempted it numerous times without success? Some individuals achieve success in their fertility journey even at the age of 50. I am less than 40, I always told myself that you can always do it if you work hard considering the advancements in technology. However, the reality is disheartening—people succeed at 50, but here I am at 38, facing failure with seemingly no solution. (P3)

Another participant expressed her confusion and frustration with repeated failures:

After so much hard work and effort, why is it always me? Other people have an easy time getting pregnant, why it is so difficult for me? Failure after failure, I really cannot figure out whether it’s a problem with my body, or if the embryo is just not strong enough. It’s a mystery to me. (P10)

*3*.*2*.*2*.*2*. *Anxiety from repeated failures*. This refers to the persistent state of anxiety that participants feel due to recurrent failure. One participant stated:

I cannot really describe the feeling in words, but it’s like a lot of anxiety. My mindset was not good. Every day, I was constantly worried. Even when I got pregnant after a successful transplant, I still felt very anxious because of the previous failures. It was like there was a shadow on my psyche, and I wonder if it will be the same as before. (P11)

The constant disappointment and state of hopelessness almost caused her to have a mental breakdown:

The first time, it was fine, and I did not feel much when the treatment failed. However, the second and third times were different—it’s a cycle of hope and disappointment every time. Your mindset gets worsens until you finally break down. It’s a complicated feeling of anxiety. (P11)

Similarly, another participant described the intense anxiety from repeated failures:

Indeed, when discussing IVF treatment, I can endure the physical discomfort, including taking medications and injections. However, facing failures again and again can cause significant psychological suffering, resulting in a lower quality of life and heightened anxiety. (P3)

*3*.*2*.*2*.*3*. *An insurmountable obstacle*. Participants reported that repeated failures made them feel as though they were facing an insurmountable obstacle. They want to solve the problem but cannot. However, they are reluctant to give up, and unless they go through menopause, there is still a chance for them to conceive. Therefore, they continue to try to achieve fertility, often to the extent that other life goals are forgotten and their lives revolve around seeing doctors and treatments.

One participant described as follows:

I’m in a very confused state right now. I find that my life has no goals anymore. Since I started doing this, I no longer care about anything. I feel like nothing is related to me. Every day, I’m just focused on this. I am just one step away from perfection in my life, yet I find myself unable to overcome this obstacle despite expending considerable effort. I have not done anything else in the past few years. It’s really a waste of time, but now I dare not divert my energy to other things because I am no longer young and I cannot delay it anymore. (P11)

Another participant, who does not consider it necessary to have children, said the unresolved fertility issue has had a significant impact on her because of repeated treatment failures:

So, it has become a problem. It is not that I’m looking forward to having a child and then being very happy and blissful. It’s that after all these years, there are no results. I’m really agitated: when will this problem be solved? It’s like an insurmountable obstacle. I just want a result now. I don’t care whether I will have a child or not. I just want to put an end to this problem. I do not want to spend my entire life pursuing this. (P4)

Another participant stated that for her, this issue had become a mental disease:

After all, it’s like a mental disease. Unless one day you give birth to a healthy child, the disease will always exist. It is a big obstacle that’s hard to overcome. (P10)

### 3.3. Interpersonal experiences and challenges

Women undergoing IVF treatment often experience discomfort and difficulty in their social circles. They may feel guilty about their infertility, leading to a sense of isolation, especially among relatives and friends with children. These women find it challenging to share their experiences but can empathize with others facing similar issues.

#### 3.3.1. Guilt toward family

Guilt refers to feelings of shame or regret. Women who have undergone fertility treatment and failed often feel responsible for not fulfilling their perceived duty to their family. One participant expressed:

My husband was an only child, and I always feel guilty that after so many years of marriage, I have not given him a baby. It seems I am the one with the infertility issue, and there are always problems when I get pregnant. (P11)

Another participant shared:

To be honest, I feel a little guilty toward my husband. Although he always says it’s fine not to have a baby, I feel a strong need to have one. This might be influenced by traditional beliefs or the notion of what a ’normal’ woman should have. I believe I should have a child for him. (P10)

She also felt guilty toward her elderly parents-in-law:

In fact, I understand my parents-in-law. They have only one son, and the desire for a descendant is natural, especially considering their age. At times, I feel a slight sense of remorse towards them, even though they don’t express it verbally. (P10)

A participant, who received care from her during her pregnancy after IVF treatment said:

Parents are very selfless toward their children, and I am their only child. They are very concerned about me, and I feel quite guilty about them. After taking care of me for so long, their hopes were dashed. They are even sadder than I am. (P5)

#### 3.3.2. Social isolation and embarrassment

Social isolation refers to detachment from one’s social circle, while embarrassment refers to the discomfort felt in social settings. one participant who had been infertile for many years dreaded holidays:

Especially during the Spring Festival, when we meet with relatives and friends, their children come along, whereas our family has no children. It’s extremely uncomfortable. After all, we have been married for a long time. Even if they say nothing, they must gossip behind our backs, which is really embarrassing. (P10)

Another participant felt embarrassed by colleagues’ comments after returning to work:

People in the company may ask, “How come you had another miscarriage?” and there’s a little bit of teasing in their speech. (P5)

As infertile women age, social isolation increases. one participant said:

All my friends have children, while socializing, each talk about their children, so I don’t know what to do. I feel very lost, a feeling I didn’t have before. Now, everyone around you has children except you! You feel lost, as if you have been cut off from their circle. (P11)

#### 3.3.3. Something you cannot discuss everywhere

Most participants chose to conceal their treatment from friends and family, feeling that their infertility is a deeply private matter.

I don’t like to let my friends know too much about it. I went for IVF treatment and did not tell them. I always feel that this is not something that can be discussed everywhere. It’s enough for my husband and me to know about it. (P11)

This concealment led to feelings of isolation and difficulty in expressing themselves when asked about their situation.

As one participant noted,

When we meet at gatherings, they always ask me how I’m going. It’s difficult to explain, and I do not want to say too much. There is no point in talking about it.” (P10)

#### 3.3.4. Lack of understanding from others

Participants felt that others, including family members, did not understand their experience. One participant felt misunderstood by her mother-in-law when treatment failed:

She was not with me during infertility treatment, did not see me getting injections or rushing to hospitals. So, every time I failed, she seemed more uncomfortable than me. I’m already unhappy, and she sometimes has a sour face, making me feel even worse.” (P3)

Another participant noted:

Those who have given birth to children find it hard to understand my situation. It’s useless to talk to them about it. They cannot know what I’m going through unless they experience it themselves. (P11)

#### 3.3.5. Mutual sympathy and support among people with similar experiences

Despite social awkwardness, women undergoing IVF treatment find mutual supports and understanding among fellow patients. This shared experience provides them with comfort and strength, making their journey less lonely.

One participant who has experienced multiple treatment failures and made friends with similar experiences said:

We all have the same experiences, we can talk and understand each other’s feelings. We can discuss anything openly. People who have not experienced IVF may not understand these feelings. However, in the company of friends with similar experiences, they feel comfortable discussing anything, providing a valuable outlet for stress relief. (P3)

Another participant noted:

We share many common experiences and similar emotions when talking together. Some experiences can serve as mutual references, such as which hospitals to visit or which doctors are recommended. Talking to such people makes me feel better when I’m feeling down. (P10)

## 4. Discussion

In the present study, a number of infertile Chinese women did not have an adequate understanding before undergoing IVF treatment. They often enter treatment with high expectations, finding the process harder than imagined, not only because of the challenges involved but also due to the outcomes. The gap between psychological expectations and outcomes results in disappointment and misery. Similar findings, such as inadequate assessment of the risk of treatment failure leading to additional emotional pain, were reported in previous research [19, 29–31]. Women’s expectations are typically based on their knowledge and confidence in IVF. Advancements in medical technology and the influence of the cultural spirit of “hard work, harvest” have caused many infertile Chinese women to have high expectations when starting IVF treatment cycles [32]. Additionally, some have little understanding of IVF treatment due to minimal communication with their healthcare providers, often because there are far more patients than doctors, especially in large medical centers where numerous patients flock to a few specialists [33].

Therefore, it is necessary to provide comprehensive and realistic information about IVF procedures, risks, and success rates. Educating patients about the potential emotional and physical challenges. This approach will lower women’s psychological expectations and help alleviate their sense of loss of control over the outcome. In our study, two participants who were familiar with treatment procedures adjusted better after treatment failure than those who lacked understanding. Moreover, fostering better communication between healthcare providers and patients is essential. Ensuring that patients have ample opportunities to ask questions and discuss their concerns is crucial. Implementing training programs for healthcare providers on empathetic communication can be beneficial. Additionally, relevant policymakers should consider reforms to ensure more balanced access to medical resources.

Consistent related studies [19, 34, 35], many participants experienced social awkwardness and discomfort, finding it difficult to share their fertility experiences and gain others’ understanding. They encountered awkward situations when competitive colleagues made malicious jokes and others expressed concern about their fertility. This phenomenon may be related to the “stigma” attached to infertility. The concept of “Stigma”, introduced by Goffman [36] and used in the medical field to describe characteristics that individuals feel ashamed of due to abnormalities like physical disabilities or personality defects, reflects society’s negative attitudes toward certain groups. Infertile women are often considered “abnormal” and “defective” because of their impaired fertility, leading to stigma, shame, and even domestic violence [37, 38].

Stigma can be classified into perceived, enacted, and self-stigma [39]. In our study, five participants reported feeling embarrassed to share their infertility with others, fearing the risk of negative judgment from the outside world. Only one participant experienced stigma due to comments made by colleagues after a failed IVF transfer. This aligns with studies showing that the stigma felt by infertile Chinese women mainly comes from internal factors [40–42]. This may be due to the traditional value of carrying on the family line is deeply embedded in Chinese women’s minds, leading them to view childbearing as a family obligation [22, 43]. Consequently, it is essential that public awareness campaigns be developed to reduce the stigma associated with infertility. Healthcare providers should assist infertile women in understanding the various factors that cause infertility to alleviate feelings of awkwardness and guilt. Additionally, educating the public about infertility can foster a more supportive and understanding environment. Establishing support networks where women can share their experiences and receive empathy from others facing similar challenges can provide emotional support and reduce feelings of social isolation.

As we have found, accessing IVF treatment not only affects couples financially but also impacts their physical and emotional well-being [6, 44, 45]. Treatment failure can lead to various adverse psychological effects [8, 9, 19]. After each unsuccessful treatment cycle, women often need time to come to terms with what happened, during which they may cry and struggle with their emotions [18, 46]. They then use coping strategies such as distracting their attention, comparing downwards and seeking spiritual support to cope with the failures [46–48]. Repeated failures and adjustments may cause them to become exhausted [49]. As discovered in the present study, prolonged and repeated failures can result in anxiety and depression, making women feel like their entire life revolves around infertility and treatment, causing them to lack interest in anything else. They become exhausted from this way of life, which is time-consuming and seemingly endless. It is as if they have come up against an insurmountable obstacle in life and are unable to move forward, causing them immense pain.

Studies have found that infertile women who discontinue treatment often adjust to their new lives by developing acceptance, finding meaning, and pursuing new life goals [50, 51]. However, unlike in Western countries, where couples may stop IVF treatment after two or three failed cycles [48], Chinese couples typically insist on having a biological child to carry on the family bloodline and reject the idea of adoption or living child-free [52]. Consequently, most infertile women in China persist with infertility treatments even when doctors have told them their chances of success are slim. Therefore, it is crucial to find ways to improve the psychological state and quality of life of infertile women during treatment. As described in previous literature [53], acceptance and finding alternative identities or goals can help infertile women feel less distressed. This is also consistent with the view that individuals who work outside have better psychological conditions than homemakers [54, 55]. In the present study, one participant demonstrated acceptance of childlessness and planned to give up treatment if it was unsuccessful after they crossed the age of 40 years. She exhibited optimism and calmness during the treatment process.

Therefore, healthcare providers should encourage infertile women to look for a good quality of life beyond focusing solely on treatment. This includes promoting the acceptance of adoption and child-free living as valid and fulfilling options. Additionally, encouraging infertile women to remain engaged in work and social activities can improve their psychological well-being. Employers can be educated on the importance of supporting employees undergoing infertility treatments and creating a more accommodating work environment. Healthcare providers can also develop psychological support programs that help infertile women cope with treatment stress and develop acceptance of different life paths. These programs could include individual counseling, support groups, and workshops that teach effective coping strategies and resilience.

This study has some limitations. First, all the participants come from Zhejiang Province, which is located in the southeast and is considered a more developed region of China; therefore, the results may not apply to the whole country, especially remote areas in the west. Second, the principle of maximum variation in the sample is not fully reflected in the study population in terms of occupation, education level, IVF treatment cycles, and time of the last failed treatment due to study constraints. Finally, the study considered a relatively small sample size. Although data saturation was reached, future research studies should have larger sample sizes so that other themes might be identified through infertile women’s experiences and stronger conclusions can be reached. Future studies should focus on longitudinal research to track the psychological impact of IVF treatment over time, and psychological interventions should be implemented as well.

## 5. Conclusion

This study explored the experiences of infertile Chinese women undergoing IVF treatment and the psychological challenges they face after treatment failure. The results indicate that these women considered IVF as an insurmountable challenge, experiencing social awkwardness, unexpected difficulty, and psychological duress. Repeated failures lead to trauma, internal conflict, and psychological disorders.

The findings offer insight into the suffering of infertile women and highlight the need for support to help them navigate the challenges. The study provides a specific view of the IVF process for infertile women, suggesting that policymakers should design tailored interventions for those undergoing IVF treatment.

## Supporting information

S1 Data (ZIP)

## References

[1] Zegers-Hochschild F, Adamson GD, Dyer S, Racowsky C, de Mouzon J, Sokol R, et al. The international glossary on infertility and fertility care, 2017. Fertil Steril. 2017 Sep; 108(3): 393–406. doi: 10.1016/j.fertnstert.2017.06.005 28760517

[2] Vander Borght M, Wyns C. Fertility and infertility: Definition and epidemiology. Clin Biochem. 2018 Dec; 62: 2–10. doi: 10.1016/j.clinbiochem.2018.03.012 29555319

[3] Jain M, Singh M. Assisted Reproductive Technology (ART) Techniques. 2023 Jun 7. In: StatPearls [Internet]. Treasure Island (FL): StatPearls Publishing; 2024 Jan–. 35015434

[4] Chambers GM, Dyer S, Zegers-Hochschild F, de Mouzon J, Ishihara O, Banker M, et al. International Committee for Monitoring Assisted Reproductive Technologies world report: assisted reproductive technology, 2014†. Hum Reprod. 2021 Oct 18;36(11):2921–2934. doi: 10.1093/humrep/deab198 . 34601605

[5] Boivin J, Griffiths E, Venetis CA. Emotional distress in infertile women and failure of assisted reproductive technologies: meta-analysis of prospective psychosocial studies. BMJ. 2011 Feb 23; 342: d223. doi: 10.1136/bmj.d223 21345903 PMC3043530

[6] Ni Y, Tong C, Huang L, Zhou W, Zhang A. The analysis of fertility quality of life and the influencing factors of patients with repeated implantation failure. Health Qual Life Outcomes. 2021 Jan 25;19(1):32. doi: 10.1186/s12955-021-01666-3 33494768 PMC7831164

[7] Stanhiser J, Steiner AZ. Psychosocial aspects of fertility and assisted reproductive technology. Obstet Gynecol Clin North Am. 2018 Sep; 45(3): 563–574. doi: 10.1016/j.ogc.2018.04.006 30092929

[8] Swift A, Reis P, Swanson M. Infertility-related stress and quality of life in women experiencing concurrent reproductive trauma. J Psychosom Obstet Gynaecol. 2022 Jun;43(2):171–176. doi: 10.1080/0167482X.2021.2008901 34907847

[9] Arhin SM, Mensah KB, Agbeno EK, Yirdong F, Opoku-Agyeman K, Ansah C. Psychological distress of Ghanaian couples after unsuccessful treatment for infertility. Ghana Med J. 2023 Dec;57(4):275–283. doi: 10.4314/gmj.v57i4.4 . 38957853 PMC11215225

[10] Maroufizadeh S, Karimi E, Vesali S, Omani Samani R. Anxiety and depression after failure of assisted reproductive treatment among patients experiencing infertility. Int J Gynaecol Obstet. 2015 Sep; 130(3): 253–256. doi: 10.1016/j.ijgo.2015.03.044 26100348

[11] Milazzo A, Mnatzaganian G, Elshaug AG, Hemphill SA, Hiller JE; Astute Health Study Group. Depression and anxiety outcomes associated with failed assisted reproductive technologies: A systematic review and meta-analysis. PLoS One. 2016 Nov 11; 11(11): e0165805. doi: 10.1371/journal.pone.0165805 27835654 PMC5106043

[12] Su TJ, Tzeng YL, Kuo PC. The anxiety of Taiwanese women with or without continuity treatment after previous in vitro fertilization failure. J Clin Nurs. 2011; 20:2217–2223. 21672061 10.1111/j.1365-2702.2011.03730.x

[13] Vikström J, Josefsson A, Bladh M, Sydsjö G. Mental health in women 20-23 years after IVF treatment: A swedish cross-sectional study. BMJ Open. 2015 Oct 28; 5(10): e009426. doi: 10.1136/bmjopen-2015-009426 26510732 PMC4636640

[14] Gdańska P, Drozdowicz-Jastrzębska E, Grzechocińska B, Radziwon-Zaleska M, Węgrzyn P, Wielgoś M. Anxiety and depression in women undergoing infertility treatment. Ginekol Pol. 2017; 88(2): 109–112. doi: 10.5603/GP.a2017.0019 28326521

[15] Zeren F, Gürsoy E, Çolak E. The quality of life and dyadic adjustment of couples receiving infertility treatment. Afr J Reprod Health. 2019 Mar; 23(1): 117–127. doi: 10.29063/ajrh2019/v23i1.12 31034178

[16] Massarotti C, Gentile G, Ferreccio C, Scaruffi P, Remorgida V, Anserini P. Impact of infertility and infertility treatments on quality of life and levels of anxiety and depression in women undergoing in vitro fertilization. Gynecol Endocrinol. 2019 Jun; 35(6): 485–489. doi: 10.1080/09513590.2018.1540575 30612477

[17] de Castro MHM, Mendonça CR, Noll M, de Abreu Tacon FS, do Amaral WN. Psychosocial aspects of gestational grief in women undergoing infertility treatment: A systematic review of qualitative and quantitative evidence. Int J Environ Res Public Health. 2021 Dec 13; 18(24): 13143. doi: 10.3390/ijerph182413143 34948752 PMC8701103

[18] Fieldsend M, Smith JA. ’Either stay grieving, or deal with it’: the psychological impact of involuntary childlessness for women living in midlife. Hum Reprod. 2020 Apr 28; 35(4): 876–885. doi: 10.1093/humrep/deaa033 32268357

[19] Harris DL, Daniluk JC. The experience of spontaneous pregnancy loss for infertile women who have conceived through assisted reproduction technology. Hum Reprod. 2010 Mar; 25(3): 714–720. doi: 10.1093/humrep/dep445 20023296

[20] Li J, Luo H, Long L. A qualitative investigation of the experience of participation in mindfulness-based intervention for IVF-ET (MBII) with Chinese women undergoing first IVF-ET. Nurs Open. 2018 Dec 18; 6(2): 493–503. doi: 10.1002/nop2.232 30918700 PMC6419106

[21] Peddie VL, van Teijlingen E, Bhattacharya S. A qualitative study of women’s decision-making at the end of IVF treatment. Hum Reprod. 2005 Jul; 20(7): 1944–1951. doi: 10.1093/humrep/deh857 15802323

[22] Hwu LJ, Hsu MY, Chuang HL, Shih FF, Lu YA, Lee SH. Childbearing Perceptions Among Taiwanese Women Undergoing In Vitro Fertilization Treatment: A Qualitative Study. J Transcult Nurs. 2022 Sep;33(5):569–575. doi: 10.1177/10436596221103249 . 35684959

[23] Auerbach C, Silverstein L B. Qualitative data: An introduction to coding and analysis. New York: New York University Press; 2003.

[24] Lincoln Y S., Guba E. G. Naturalistic inquiry. Beverly Hills, CA: Sage; 1985.

[25] Sandelowski M. Whatever happened to qualitative description? Res Nurs Health. 2000; 23:334–340. doi: 10.1002/1098-240x(200008)23:4&lt;334::aid-nur9&gt;3.0.co;2-g 10940958

[26] Colorafi KJ, Evans B. Qualitative descriptive methods in health science research. HERD. 2016 Jul; 9(4): 16–25. doi: 10.1177/1937586715614171 26791375 PMC7586301

[27] Moser A, Korstjens I. Series: Practical guidance to qualitative research. Part 3: Sampling, data collection and analysis. Eur J Gen Pract. 2018 Dec;24(1):9–18. doi: 10.1080/13814788.2017.1375091 . 29199486 PMC5774281

[28] Roller MR. A quality approach to qualitative content analysis: similarities and differences compared to other qualitative methods. Forum Qual Soc Res. 2019; 20(3): 31.

[29] Ranjbar F, Behboodi-Moghadam Z, Borimnejad L, Ghaffari SR, Akhondi MM. Experiences of infertile women seeking assisted pregnancy in Iran: A Qualitative Study. J Reprod Infertil. 2015 Oct-Dec; 16(4): 221–228. 27110521 PMC4819212

[30] Devroe J, Peeraer K, D’Hooghe TM, Boivin J, Laenen A, Vriens J, et al. Great expectations of IVF patients: the role of gender, dispositional optimism and shared IVF prognoses. Hum Reprod. 2022 May 3; 37(5): 997–1006. doi: 10.1093/humrep/deac038 35213695

[31] Miron-Shatz T, Holzer H, Revel A, Weissman A, Tarashandegan D, Hurwitz A, et al. ’Luckily, I don’t believe in statistics’: survey of women’s understanding of chance of success with futile fertility treatments. Reprod Biomed Online. 2021 Feb;42(2):463–470. doi: 10.1016/j.rbmo.2020.09.026 . 33250411

[32] Cx Zhang, Dd Wang, Yang xn. The impact of psychoeducation on women undergoing IVF treatment. Maternal and Child Health Care of China. 2010; 25: 1181–1182. (in Chinese)

[33] Yuan L, Cao J, Wang D, Yu D, Liu G, Qian Z. Regional disparities and influencing factors of high quality medical resources distribution in China. Int J Equity Health. 2023 Jan 10; 22(1): 8. doi: 10.1186/s12939-023-01825-6 36627636 PMC9832614

[34] Janković I, Todorović J. Lived experiences of woman in relation to infertility–a review of the qualitative research[J]. Facta Universitatis, series: Philosophy, Sociology, Psychology and History, 2021: 137–148.

[35] Tome M, Zwahlen E. Lived experience of infertility and in vitro fertilisation treatment. Aust J Gen Pract. 2023 May;52(5):295–297. doi: 10.31128/AJGP-09-22-6566 . 37149768

[36] Goffman E. Stigma: Notes on the Management of a Spoiled Identity. New York: Simon and Schuster; 1968.

[37] Öztürk R, Bloom TL, Li Y, Bullock LFC. Stress, stigma, violence experiences and social support of US infertile women. J Reprod Infant Psychol. 2021 Apr; 39(2): 205–217. doi: 10.1080/02646838.2020.1754373 32338526

[38] Sharifi F, Jamali J, Larki M, Roudsari RL. Domestic violence against infertile women: A systematic review and meta-analysis. Sultan Qaboos Univ Med J. 2022 Feb; 22(1): 14–27. doi: 10.18295/squmj.5.2021.075 35299802 PMC8904118

[39] Pescosolido BA, Martin JK. The Stigma Complex. Annu Rev Sociol. 2015 Aug; 41: 87–116. pimd: doi: 10.1146/annurev-soc-071312-145702 . 26855471 PMC4737963

[40] Qian N, Zhai J, Li L, Li Y, influencing factors of stigma in female patients with infertility. Journal Of Nursing Science. 2021; 36:69–72. (in Chinese)

[41] Luo D, Zhouchen YB, Li L, Jiang YL, Liu Y, Redding SR, et al. The Stigma and Infertility-Related Stress of Chinese Infertile Women: A Cross-S. doi: 10.3390/healthcare12111053 . 38891128 PMC11171736

[42] Lin YT, Wang AW, Wei S, Hong JS, Hsu WY. The relationship between infertility family stigma, self-stigma and psychological well-being in female first-visit patients at a reproductive medicine center in Taiwan. Health Psychol Rep. 2021 Jun 28;10(2):122–128. doi: 10.5114/hpr.2021.107335 . 38084326 PMC10681834

[43] Tiu MM, Hong JY, Cheng VS, Kam CY, Ng BT. Lived experience of infertility among Hong Kong Chinese women. Int J Qual Stud Health Well-being. 2018 Dec; 13(1): 1554023. doi: 10.1080/17482631.2018.1554023 30704372 PMC6319451

[44] Öztürk R, Herbell K, Morton J, Bloom T. "The worst time of my life": Treatment-related stress and unmet needs of women living with infertility. J Community Psychol. 2021 Jul; 49(5): 1121–1133. doi: 10.1002/jcop.22527 33616236 PMC8324009

[45] Iordachescu DA, Gica C, Vladislav EO, Panaitescu AM, Peltecu G, Furtuna ME, et al. Emotional disorders, marital adaptation and the moderating role of social support for couples under treatment for infertility. Ginekol Pol. 2021;92(2):98–104. doi: 10.5603/GP.a2020.0173 . 33448003

[46] Kissiwaa AVM, Fouché N. Ghanaian women’s experiences of unsuccessful in-vitro fertilisation treatment, unravelling their meanings: a Heideggerian hermeneutic phenomenological study. BMC Pregnancy Childbirth. 2024 Mar 20;24(1):212. doi: 10.1186/s12884-024-06365-7 . 38509466 PMC10956244

[47] Ying XX, Xu XL, Jin Y, Zhou YX. A qualitative research on the experience of women who miscarriage after conceived through IVF-ET treatment. Clinical Education of General Practice. 2022; 03: 281–283. (in Chinese)

[48] Mesquita da Silva S, Place JM, Boivin J, Gameiro S. Failure after fertility treatment: regulation strategies when facing a blocked parenthood goal. Hum Fertil (Camb). 2020 Sep;23(3):179–185. doi: 10.1080/14647273.2018.1510186 . 30253679

[49] Patel A, Sharma PSVN, Kumar P. "In Cycles of Dreams, Despair, and Desperation:" Research Perspectives on Infertility Specific Distress in Patients Undergoing Fertility Treatments. J Hum Reprod Sci. 2018 Oct-Dec;11(4):320–328. doi: 10.4103/jhrs.JHRS_42_18 . 30787515 PMC6333040

[50] Su TJ, Chen YC. Transforming hope: the lived experience of infertile women who terminated treatment after in vitro fertilization failure. J Nurs Res. 2006 Mar; 14(1): 46–54. doi: 10.1097/01.jnr.0000387561.03823.8e 16547905

[51] Copp T, Kvesic D, Lieberman D, Bateson D, McCaffery KJ. ’Your hopes can run away with your realistic expectations’: a qualitative study of women and men’s decision-making when undergoing multiple cycles of IVF. Hum Reprod Open. 2020 Dec 23;2020(4): hoaa059. doi: 10.1093/hropen/hoaa059 . 33392395 PMC7757429

[52] Lee GL, Hui Choi WH, Chan CH, Chan CL, Ng EH. Life after unsuccessful IVF treatment in an assisted reproduction unit: a qualitative analysis of gains through loss among Chinese persons in Hong Kong. Hum Reprod. 2009 Aug; 24(8): 1920–1929. doi: 10.1093/humrep/dep091 19372145

[53] Gameiro S, Finnigan A. Long-term adjustment to unmet parenthood goals following ART: a systematic review and meta-analysis. Hum Reprod Update. 2017 May 1; 23(3): 322–337. doi: 10.1093/humupd/dmx001 28164236

[54] Zurlo MC, Cattaneo Della Volta MF, Vallone F. Infertility-Related Stress and Psychological Health Outcomes in Infertile Couples Undergoing Medical Treatments: Testing a Multi-dimensional Model. J Clin Psychol Med Settings. 2020 Dec;27(4):662–676. doi: 10.1007/s10880-019-09653-z . 31471847

[55] Ikemoto Y, Kuroda K, Endo M, Tanaka A, Sugiyama R, Nakagawa K, et al. Analysis of severe psychological stressors in women during fertility treatment: Japan-Female employment and mental health in assisted reproductive technology (J-FEMA) study. Arch Gynecol Obstet. 2021 Jan 1; 304: 253–261. doi: 10.1007/s00404-020-05923-6 33386414 PMC7775729

