# Supplementary material for: An insurmountable obstacle: Experiences of Chinese women undergoing in vitro fertilization
Source: PLoS One. 2024 Oct 7;19(10):e0311660. doi: 10.1371/journal.pone.0311660 (PMC11458033; doi:10.1371/journal.pone.0311660)
Supplement: S1 Data — (ZIP) [file pone.0311660.s001.zip › data/P6.docx]

R：我看了一下你的病史，包括你们老公也经常在跟我讲的，我觉得你们可能一直也好多年了，一步步走过来，就是谈谈你的一些感受，好吗？

P：哦行，其实刚开始我们做这个，本来不是自己，不是说一开始就是要做这个的，我有个朋友，她去上海仁济那边做试管，然后我们刚开始也没有想着生孩子什么的，后来他看我们也很挺多年了，那时候三年多了，然后她说要不要一起去检查，然后去检查一起去的，一起去，然后医生就说卵巢功能他说很差了，他说你还是赶紧做试管吧，那时候觉得做试管这种东西想都没想过，后来，但是也还是——那时候刚开始没做，就做了宫腔镜，其实想着还要不自己再试试，然后但是后来没怀嘛就想着要不去做做看。然后，然后反正人家都说，然后我家里我也没跟我家里人说，后来我妈知道了，然后我妈就觉得做这种东西可能有点太辛——因为大家的心里就觉得很辛苦，就是说很痛很什么啊，但是然后我说我决定了，先试试看，然后我觉得其实开始了也就那样，也没有说很痛什么，因为我一开始就是短方案，短方案很快的嘛，就是月经第二天去，开始就打针什么到排卵也就十几二十天就不是可以取卵了嘛，那时候上海取卵不是全麻的嘛，所以他们说出来很痛的，我也没觉得，反而我觉得做宫腔镜很痛的。那后来反正，反正就觉得那时候觉得可能心里想着做试管了好像就能成功，就感觉一次就能成功的那种，后来第一次，第一次也没，反正移植了以后也没成功。第一次是真的没成功不是说很难，我觉得第二次没成功是最难受的时候，第一次我觉得也还好，反正也没成功就想着大不了下次再开始呗，然后后来第二次去，反正我觉得也没有说像大家想的那种做试管就是会很痛什么，我觉得可能最辛苦的就是心理上的那个累，就是反正到取卵的步骤，我就觉得还行，因为打的针什么，我反正没碰到过很痛的针，不是都是那种笔嘛很小的针孔。然后我都觉得还好，反正我觉得移植后心里真的会很难受。第一次因为我爸妈，我妈是知道我们要做，但是不知道具体的时间，所以没成功就我们两个人知道，所以也就那样，反正就当天过了就过了，然后第二次是我妈也知道的，然后感觉家里人知道了以后就会压力更大，他就是一直要问你的，就是说为什么不成功，怎么为什么什么的，然后第二次就觉得心里压力，那时候第二次也是上海做的，然后那次反正打击挺大（笑），就想要不去了。

R：为什么？

P：就觉得做了两次都没成功，而且因为取的也少嘛，每次移好以后不成功又要重新再取，然后想着要么不去了，去上海又也挺麻烦的，也挺远的。主要是来回麻烦，因为隔一两天就要去。

R：为什么第二次打击最大？

P：第二次，我说不出来那种感觉，可能就是第一次嘛不成功，就觉得第二次肯定总能成功什么，因为觉得人家第一次成功的很多，然后第一次就算不成功，好像第二次——因为心里想着就算一次不成功，第二次总是能成功的，结果第二次还是没有成功，就感觉，就是觉得好像没有什么希望，反正那时候就觉得唉要不算了。后来正好我们那边有个朋友，他在杭州的，他说要不去邵逸夫看看，然后反正也正好是朋友带着过来的，反正来医院了就是很快的就又可以重新开始了。然后反正，也反正我每次都是短方案，就是很快的，就是月经第二天来就开始打针什么。然后第三次是着床的，着床以后反正没翻倍。正好翻了1倍的那种。那时候，那时候不知道要保胎什么，因为取卵的时候他们那边西医就说不要喝中药什么的，就取卵的时候嘛，那就想着，那就觉得移植后可能也不能喝什么的，就也没有想着去要保胎，反正邵逸夫那边他们就是翻倍看个两次，我看了两次。然后他说就等移植后二十几天做B超，然后反正二十几天去的时候，本来应该二十几天就是看宫内宫外的，然后反正看了一下，就说三十几天再来。

R：你当时宫腔也是看到的？

P：对，然后35天去的时候，B超查出来胎停了，然后医生就直接说你要药流还是人流，我当时就直接懵掉了，因为那时候完全没有想过会这样的。然后我就问我老公，我老公也懵了，他说要不先回去再说，然后就先回去的，回去以后，然后我妈就说要不要去喝喝中药。然后后来，那时候喝中药其实已经没什么用了，因为已经太晚了嘛。但是喝还是喝的，喝了两三天，就自己出血了，也没有去做人流。就反正自己流掉的。

R：当时的感受是怎么样的？——那个你就看情况，你如果觉得回忆什么影响你——

P：那没有那没有，我们心态还是很好的。

R：是啊我看你们也是心态不错。起码就不能影响到现在——累了和我说

P：哦没有不会的。我们心态还是很好的，失败了就过了。就是，在医院那天，就是医生说不好的，就说要药流人流，那时候就是反正，反正我觉得，我不知道，反正就是整个人都懵了，然后我就我妈那时候，我妈是知道的，就那时候都是我妈只有我妈知道的，然后她就一直打我电话，她可能就想问一下怎么样，然后我就懒，什么都不想回，我就蹲在那边，然后我跟我老公就在那里发呆，真的不知道，其实不知道什么感受，就是整个人就是——

R：不知所措？

P：对，真的是这样的，也不是说，反正当时的感受就是整个人都不知道在干嘛，反正在那里，哭也没有哭，就是很懵，然后回到家倒是哭了（笑）。当时是没有的，就是那个，当天，到第二天，反正就还好了（笑）。后来因为也没办法嘛，就这样嘛，那就只能这样了，而且反正已经做了那么多次了，想想，想想唉反正那么多次了，然后，然后就想着我老公就是说，大不了下次再来说！我说那也只能这样了，不然怎么办？

R：后来你是觉得就这个过程你自己感觉是怎么调整过来的？

P：我没有调整，我觉得我们——（笑）

R：也就这样想开了？

P：对，真的我觉得我想，也没有说怎么想不开，反正就在邵逸夫那天医院出来那天会很难受，觉得特别特别失望，觉得。就是对自己很失望，就是这样。但是回去第二天我就觉得，反正我觉得到，起码是到第三天没事就还好了。

R：想法是怎么过渡过来的，一般人比如说碰到这种情况可能会比较失落或什么的，你能想这么开你是怎么想的呢？

P：我，反正我想着，我是想着再，大不了就再做一次，如果下一次再不成的话就算了。就不做了，本来主要是因为，我们两个，我不知道我老公可能还好，就是我对小孩子其实没有特别，就不是说很喜欢，不是说一定要的那种。其实本来也是觉得年纪也大了。没有，不生孩子以后肯定会后悔的，因为现在就算不想要，以后估计也是要的，所以还不如就是趁现在还是要生的，所以那时候反正没有了。后来也，真的也还好，反正我是想着大不了再去做一次，做一次以后如果不成就不做了，要不就自己自然怀孕再试试。要不就不做了，就反正是就抱着这种心态的。

R：但据我所知，你后来又做了很多次，是不是？

P：没有，就这次嘛，一共就四次。

R：好像听你老公说有八次嘛？

P：她乱说（笑），他自己都搞不清楚，就上海做了两次，这里做了两次，是这样子。

R：（笑）我是看看你病史上好像没几次。

P：对，他没有，他自己估计搞不清楚了，他这个过程可能——

R：他会不会把取卵也上去了。

P：对，可能是这样。就上海做了两次，这里做了两次。

R：那你当时流产后后，有没有感受到周围一些包括人际关系家里的变化这种？

P：也，因为没，我们家里只有我妈知道。我们没有告诉周围的亲戚他们，因为之前失败过嘛所以就怕最后结果还是会不好。刚刚开始呢，就是我妈以前还是会催我们的，就是她不知道我们做试管前面就催我们要生孩子什么的。然后我公婆反正在我们面前是不怎么说的，因为我老公有时候还挺凶的，就是很那个的，所以他们在我们面前有时候我也会，如果他们那个的话，我有时候也会挺那个的。他们在我们面前是不敢说的，背后肯定也是希望生的，对吧？但是在我面前没说的，然后只有我妈知道的，我妈知道我们做试管开始呢就是也不是催我们生了，就是问我们怎么样啦 ，或者什么时候去医院或者怎么样。然后就那次，那次就上一次失败以后，然后她就不问我了，也不催我们，也不问我们，就是有时候她可能想问又觉得可能怕我有压力，后来就不问我们了。因为我就是我不太喜欢跟人家跟我爸妈沟通这种事情的，如果我想跟你说我会跟你说的，如果我不想说，你问我我也不会说的那种，所以我妈知道她如果我想跟她说我也会说的，就像我这次我们又来杭州，我会跟他说，我说我们明天要去杭州，那她肯定就知道我们去干嘛的，然后反正她后来就不会给我给我们压力了，刚开始啦她就是，觉得我们好像没做努力什么的，还是会催的。有时候当着那种亲戚的面什么，就觉得我就会发火。然后知道我们做试管开始她就也不怎么说了，但是因为我们身边知道的人也不多嘛。

R：就你妈知道？

P：这次是都知道了。以前是只有我妈知道，他们也，我公婆，我爸可能也知道我们在看，但是可能也不知道我们在做试管这种，这次是因为反正也想开了，当时刚开始觉得做试管还算有点好意思，就是不太想跟人家说。

R：为什么？

P：就觉得，我不知道，就觉得可能农村里的那种思想，自己可能也有，也不是说怪人家有这种思想，刚刚开始自己也有的，就感觉没怀孕，然后又要去做试管。反正就是农村里那种很传统的思想，就也不想跟人家说。这次我觉得反正，反正就这样的，就这么多年没生了，大家肯定心里也是有想法的，不管是那种亲戚朋友，还是公婆的那种朋友，肯定心里有想法的，不管反正就算知道了也没事，而且毕竟现在做试管的也很多了，我们那边亲戚啊什么也有，就亲戚的那种亲戚啊或者那种农村里边的隔壁啊邻居啊也还是也有这种，大家都可能对这方面也看开了，就觉得你没怀孕就应该去看，就是有时候有人家他们也会，就是我婆婆的朋友或者是那时候，我婆婆有一帮念经的那种朋友，就大妈妈那种，人还挺好的，就会说你们要不要去做试管什么的，那我们也没说我们在做，就说我们在看，就是会，在考虑。他说对反正他们就会说谁谁谁的女儿也是做试管的，现在孩子很可爱的啊什么的，那我觉得他们也不是恶意也是为你好的，就觉得我们会去看的什么的。

R：那有没有受到一些传统的那种，比如说类似有一种看低就是会让你有点不太好意思啊这种思想的影响，就是说有没有受到别人的这种不一样的眼光这种？

P：那到那我觉得倒没有，因为现在目前，因为也就这次大家都知道，可能大家有些亲戚朋友都知道的，有时候有些他们也来这里看过，看我们的，那就是就觉得反正从他们来看看我们的那些人，你就觉得他们应该还是挺替我们开心的，对，毕竟你说这么多年了，不管是试管做的还是怎么样的。。。。。（会议室有人敲门,同事找我有点事。）

2分钟后 ——

R：我们刚刚谈到哪了——谈到你家里人，谈到你们家那边有没有这种思想的束缚什么的？

P：对，反正现在，反正就是我觉得，反正在通过来看我们的那些亲戚朋友就觉得还好，就是他们反正也挺替我们开心的反正。其他人反正也管不了。

R：所以一开始还是你们自己心里反而有这种顾虑。

P：反正我心里还是有这种顾虑的，然后我老公我就不知道了（笑），他反正应该还好吧。

R：他我看还好的（笑）。

P：对啊，这个男的跟女的可能想法还是有点不一样的。

R：对对，那么这次怀孕后是什么感觉？

P：这次我反正，到现在我都没有跟很多人说，就跟，我自己反正就跟，我妈反正知道的嘛，我们自己那边亲戚我们也没怎么讲，因为以前生化过就很怕那时候，很怕又不好又怎么样，后面反正我每次跟我妈说，人家一超没过，一超过了，二超没过的也很多，二超过了IT没过的也很多，反正就什么都要想的，就是万一现在高兴死了以后又有什么问题啦什么的，反正现在就也没有特别感觉自己说是怀孕什么的，不知道是自己一直在暗示自己还是，反正也没有说特别的明显地感觉说自己怀孕了很开心啊什么，反而我老公我觉得他好像比较明显（笑），他就每天就说我女儿什么什么，我说现在才那么一点点。

R：他也不是的，他在我们面前其实也都说的。

P：他没有，他可能就是想让我放松一点，因为毕竟来看，有时候以前没事干嘛，就逛逛那种论坛什么的，人家不是很多的，就那种怎么没过啊四维又不好，什么又不好，以后就是什么畸形的啊，唐筛没过的啊，反正不是很多的嘛，然后我就反正想着也不，也不知道，反正到三个月以后可能会，会心里会踏实一点，现在也不敢怎么样。。。

R：感觉还在闯关。

P：对，真的是这样的。

R： 那你觉得你家里人的一些态度啊，包括他们对你的一些，一些想法什么有没有影响你的地方？

P：我觉得应该没有的。我家里人因为我们两个，我跟我老公两个就是从小自我意识就很强，我们家里人都知道，不能干涉我们太多，也不能在我面前说，说太多东西，反正他们说了，我们要觉得烦了，就会，就是怼他们什么的，就是脾气就会变很差，然后所以我们家里人应该不太会在我们面前表达太多，但是我妈那次来我就觉得也挺烦的，就是他们有一种怎么说呢，得寸进尺的那种感觉，就是你，没有的时候，他们觉得有一个也好的，有了吧就想着为什么不是两个，她会有这种，我觉得她没有说，但是我觉得从她的想法里，而且也不单单是她了，就是我婆婆就是会给她打电话，因为我婆婆不敢给我们打电话因为我老公要骂的。所以他就是老是给她，一天到晚给我妈打电话，就说，说，她说我婆婆就是挺迷信的嘛，就说拜菩萨去，就是说这次肯定是两个儿子，做梦也做到两个儿子什么，那我觉得从她这种说法里，肯定是觉得，可能希望是两个或者是儿子什么的。所以我就觉得他们这方面就是挺那个的，没有的时候吧肯定觉得不管男女你生一个也好的，现在反正做试管了，他们就觉得人家好像都是双胞胎。然后你一个他们就会觉得怎么，为什么是一个啊。我跟我老公说你，我说你就说我们就放了一个就好了，不想跟他，因为这种解释不清楚的是吧？他们就觉得，他们特别是我婆，因为可能，可能因为我不知道我婆婆她知不知道有，我们就是邻居那种，他说可能我婆自己也觉得可能我们前面也在做试管只是没问我们，但是我们刚跟他说我们在做试管就这一次嘛，因为在很多我们农村那种就觉得试管就一次能成功的那种，就是不会说，人家医院里真的人家四五次五六次，真的好多的，看到真的很多的，但是很多人都，反正我们那边农村里都觉得做一次可能就能成，而且有双胞胎的必须是双胞胎的那种，这方面我就觉得挺烦的。他们就觉得人家，因为生双胞胎的也多，我们那边现在生双胞胎的还挺多的就是做试管，应该是大多数的都试管的吧，我觉得现在哪那么多对吧？因为我老公他们老家隔壁也是好几年没生，然后后来做试管，然后也是双胞胎儿子嘛，然后还有一户也是双胞胎，然后觉得好像做试管必须是双胞胎。然后就跟老公说，你就跟他们说，就放了一个就好。然后我跟我妈是说，我说我让他暂时不用跟亲戚说我们怀上什么的。但是我婆好像是，可能还挺开心的，反正亲戚朋友那边都打电话说，关键，关键她就，因为我有个很好的朋友，正好是我老公表哥的老婆，他们正好来看我们，就说她婆婆，在我舅舅家的时候，我婆婆打电话过去说，说是双胞胎就是成功什么的。然后我就崩溃，然后就跟我老公说，然后老公打电话过去，把她又骂了一顿了，然后我说你现在打电话有什么用啊，她都已经说过了，我说那算了，反正不管你。

P：你觉得这么多人知道对你有没有什么影响呢？比如说压力这种。

R：我觉得应该没有的，因为我们，就可能还是从小的性格关系，还挺我行我素，就是不会受家里亲戚那种，而且我老公他们那边其实亲戚走动的，也不多的，因为反正就各方面的原因，就是我婆也不太喜欢跟他们走动的，反正这我觉得也还好，就过年吃饭或者有事情吃饭才会碰到，平时不怎么会去走动的，所以我觉得也还好。

R：那你觉得你老公有没有对你产生一些影响啊什么的？不光是不好的影响，好的影响也可以。

P：他倒是应该算比较好的影响，因为他自己心里怎么想我也没问过他，因为我这个人就不太喜欢跟人家谈心什么，但是反正每次失败他还是都会安慰我的，就跟我说大不了下次再来过什么的，那反正鼓励的话都是他说的，反正都是，我有时候挺消极的时候，都是他鼓励我的。这方面还是好的多，他基本上可能自己消化一下负面情绪，喝喝酒什么的。

R：那么这么多年治疗过来什么的，就这种经历，有没有比较深切的这种感悟或者体会什么的？

P：就觉得中国人口太多了（笑），而且我觉得现在真的不孕不育的人好多，真的好多，我跟你说，每天在医院里排队，我觉得别的没什么，就是等和排队真的很烦的。好多好多人，每天你看每天早上做B超就有一百多个人，反正邵逸夫那边就是那一个区域就找不到位子，坐的就是人挤人挤死人的那种，就生殖中心门口就一块区域在里面，反正就是超多人的。

R：对你自己这方面的有没有那个？就自身的这种。

P：我也说不出来，我就觉得，自己——自己，因为当时我们不是结婚，应该是刚结婚那会吧，我那时候怀过一个的，然后因为我不想要然后就把他流掉了（笑着，很轻声的），后来想想还是很后悔的，就想着，以后要是生个女儿的话，就一定要告诉她，不要轻易的，要不就做好避孕措施，要不就不要轻易的打胎什么的，对身体确实还是有伤害的。其它的也没什么了。

P：你觉得关于你自己的这种，比如说失败了能很快调整过来的这种调整方式，你觉得有哪些因素会影响你的这种调整吗？

R：因素啊，我觉得大部分还是性格，然后，我觉得，那我觉得我老公还是很有功劳的（笑）。那起码失败了就，我记得上次失败也是夏天吧，然后失败了就想，就第二天我就约我最好的——就我最好的朋友还是知道的，然后就约着去吃小龙虾了，唉好久没吃了，一直憋着就好好的吃一顿。然后反正也就这么过去了。我觉得自己性格还是挺，挺大一方面的。

R：就找事情转移一下。

P：对对对。就从别的方面安慰一下自己，能吃，能喝点小啤酒，吃点小龙虾也挺好的。就这样，但是可能我觉得还是有些人可能比较喜欢孩子的，那就会更，更难过一点。像我这种，我就觉得我可能对孩子的感情没有说特别特别，我如果人家就亲戚朋友抱着小孩子在家里，就是过年吃饭如果碰到，我也不会想着要去逗一下或者抱一下，我就觉得挺烦的（笑）。但是要是自己的孩子肯定是不一样的感觉，对吧？但是看到别人就是有时候出去超市就看到人家孩子很吵的什么，我就觉得好烦啊，然后我老公就在说阿钗发火了阿钗发火了（笑）。很搞笑的是我就看人家很烦的孩子，我就觉得。。。（病友手机响了 ，没有接）。我觉得这方面也还是有关系，像人家就真的很想要一个孩子，但是又失败了的话就会挺难受的，对吧？我觉得我们可能这方面就好一点。然后而且我觉得我是觉得自己心理年龄还就是挺小的，还有我妈一直说我挺幼稚的，就是觉得自己还小，所以也没有说要到生孩子，虽然年龄已经到了，身体，而且已经身体的年龄已经到了，已经身体机能也已经下降了，但是心理年龄就是还没有到要孩子的那种地步。我觉得也有关系吧，就心理年龄还是比较，比较幼稚的。他们都说我们两个自己还是个孩子，就各方面就是很幼稚，就还喜欢玩啊什么，因为我们有时候，后来反正失败了以后觉得也挺，因为我们两个反正每天，就吃好晚饭，就出去哪里星巴克坐一会儿或者哪里玩一会儿，就没有孩子的束缚啊，不用说我什么时候要回家，孩子几点要睡觉什么，就想看个电影，随时都可以去看。反正我觉得挺好的，然后有时候想到就有孩子了就，以前就想到有孩子就这点会有束缚自己的时间又不太有了，然后就觉得也挺烦的。

R :所以就第一个不要了

P：对，到后来就那开始做试管失败了什么的，有时候也会有这种想法的，就是觉得失败了也还好吧，反正也不是说特别想要，反正自己，就有自己的时间也挺好的。就反正可能就这么调解吧我觉得。像他们有孩子的，想出去吃个夜宵又吃不了，对吧？让他们出去看个电影，还要凑大人的时间把孩子安顿好啊什么的。我觉得我们两个就比较自由，然后我朋友他们还说，唉还是享受现在没有孩子生活（笑）。

R：这次怀孕以后有没有感受到有哪些变化什么的吗？

P：生理上的还是？

R：心理上的。

P：心理上的——我觉得我还是很开心的，还是有期待的，但是目前还不敢太开心，就是有时候像逛淘宝什么，人家说买婴儿床什么，然后看到我就觉得要不先看看，然后又觉得先不看了，有点矛盾，还是很怕后期会发生什么不好的东西什么的，还是很那个的。但是还是会忍不住想看一点，就是关于小孩子的衣服或者自己那种，就那种反正关于怀孕的那种东西，还是会在网上看一下，但是就挺矛盾的，我觉得就又有期待，又不敢太期待，就这样。

R：你自己还有没有什么要补充的？

P：应该没有了，应该差不多都在了吧。所以我觉得其实做试管没有别人想的这么恐怖，就是以前觉得做试管像我妈刚开始也觉得做试管就是挺恐怖的，很可怕的一个事情。对我来说我觉得还好，因为我忍痛能力也挺强的。我觉得对我来说，因为邵逸夫那边也，刚开始我们那时候取卵，还是半，局麻的，不是全麻的，我也都没有觉得什么，反正我觉得身体上的痛苦真的还挺。反正对我来说，在我这方面我觉得还挺少的。但是打针带来的影响这种那就不知道了。这种影响

肯定很多，打了那么多这肯定有影响的。就是我觉得反而还是心理上的吧。但是这次移植后住在这边，我觉得反而挺轻松的，因为真的很比在家里轻松一点，我觉得。可能比较安心一点，反正前几次在家里就是老是，就老是想着会不会成功什么的，反正这次我觉得可能自己心态也放平了，就想着反正不成功——因为我不是在看付主任的，因为第一次去看的时候，他就说你这种情况他自己怀应该也可以。然后我就想这次不成功，我就自己试试，对，我就这么想的，我觉得可能心态放平了就会好一点。也没有说必须要怎么样。

R：是的，我看你。你看打针我们平时打针就看得出来。有些人打针一点点都会叫痛的。然后我看给你打针，你从来没有叫过丝毫的痛什么的。

P：是的我忍痛能力是真的还挺强的。

R：说明你心里还是蛮强大的。

P：对。我跟我弟弟就是两个极端，我就是偏离男孩子性格的，我弟弟就是跟我爸爸一样，我跟我妈妈性格一样，妈妈都就很强，就不说性格强势，就是家里各个方面的事情她都可以处理的那种，像我爸爸就是身体上一有个小痛小病就娇，我弟弟也是的，我跟我妈如果说我们想去医院了，就说明已经严重了，已经很痛了，或者很怎么样才会，一般如果小痛什么就不会想着去医院的。所以我觉得心理还是要，可能心态放平一点，可能真的会好一点，就太紧张了，真的也不好。住在家里我觉得，我刚开始住到这里，要住这里来的时候其实我也很矛盾的，因为想着你住个十几天不成功，好像也很那个的，因为我们一起移植的他们觉得就着床了再过来，也来得及，也还好。然后我老公就想着一定要住到这里来，因为很怕住在这里，回去了又失败了。但是后来住着嘛我觉得也还好，就是聊聊天啊大家都挺好的，而且住在家里其实压力也挺大的，就家里人。

R：有家里人，有可能会受家里人影响的

P：对对对，我妈来了几次，我就差点跟她吵起来（笑）。什么都不行，看会手机，还在看手机什么的很烦的，还是在这里舒服。

R：好吧，那其他的我也没什么好问的了，有问题再问你。

P：好的

R：你有什么有新的想法什么的再跟我聊好吧？

P：好的好的。
