# Supplementary material for: An insurmountable obstacle: Experiences of Chinese women undergoing in vitro fertilization
Source: PLoS One. 2024 Oct 7;19(10):e0311660. doi: 10.1371/journal.pone.0311660 (PMC11458033; doi:10.1371/journal.pone.0311660)
Supplement: S1 Data — (ZIP) [file pone.0311660.s001.zip › data/P10.docx]

R:感谢的话我就不说了，就是想首先先了解一下移植这一块的感受。

P:感受我其实从试管开始到包括一直到放进去，都挺顺利的，而且我一直是在一个比较——我自己感觉哦，就是很懵的，因为什么都不懂，然后反正就医生怎么说怎么做怎么做，包括到我移植进周我都是很懵的，因为正常的话不是说去了之后来过第二次月经才可以放吗？然后我是来过一次月经，然后就很突然的跟我说可以准备移植进周了，然后那反正就直接走流水线了。所以整个过程都是很懵的。然后心态也就是很平的哦，那反正说该怎么做就怎么做，可能要么我想想可能是不懂的关系吧，如果懂你像他们有些——我们会有很多群的，然后他们会在群里问来问去问东问西，我不问，我也没问过，因为我觉得既然XXX我也觉得没必要去问来问去。然后反正就按流水线走的，就没有什么特别的好像说，其实比较顺吧我感觉，反正就是反而到怀孕成功之后吧就觉得这样那样问题多起来了。之前真的是没什么感觉

R：有哪些问题多起来呢？

P：有可能是因为之前自己怀孕就状况比较多，然后一旦说怀孕之后，又可能担心会出现之前自己怀孕那种这样那样的问题，然后心里可能会有一种顾虑在，但是刚好又是在这个时间段，五六十天这样，刚刚又出现问题，就是说每次都这样，然后心里就可能会提前会有这种感觉在吧。就不像比如说有些做试管的可能就是怀不上，是吧？跟他们那种成功之后的这种心情肯定是不一样的，他们肯定是很欢喜啊，很喜悦，而且就会也不会说有像我们这种这么多的担心跟顾虑。然后在他们的理解应该是比较——怀孕了嘛反正就是该干嘛干嘛是吧？像我们这种就会顾虑会很多。都工作也不上班也不上是吧？就躺在医院这样，就这样子想着（苦笑）。

R:你觉得你有大概具体点都是哪些顾虑呢？

P:就害怕——就是血指不好啊，就像我开始吃——我HCG翻倍就一直刚刚好刚好的。其实我包括每次我跟这边的医生我都在说，其实很担心的，因为我有时候在会开会再看别人，他们群里会发他们就是那段时间的HCG的一个翻倍指标。那我会对比的呀，虽然说每个人的医生会说每个人的情况不一样的是吧，我只是参考一下他们，我就觉得我相比他们肯定是偏低的，比如说在20天30天这样，那我可以低一点，但是我觉得比别人低这么多肯定是不正常的，就心里开始在——唉就会想这个问题，然后就想着医生给我加药加药这样子。因为以前也是这样啊，HCG涨涨涨到后面就下降，然后胚胎检查不发育了，胎停了，就这种问题会出来。就是最害怕的事情，但这次不幸又撞上了。

R:你觉得是因为以前的经历给你带来的顾虑多一点，还是移植带来顾虑多？

P:以前吧，还是因为像我现在这次失败已经第四次，真的是每一次怀孕对我来说——唉挺煎熬的，不光是对我，对家里人也很煎熬。就像我老公说的，在我没怀孕的时候，他上班很轻松的，反正两个人也很潇洒。该玩玩该吃吃是吧！好，等到——不像人家说怀孕了可兴奋了，他没，反而更焦虑。因为时间长啊很漫长，然后特别——要到这个五六十天的时候，（苦笑 ）可能是，也有可能是前面事情出的多了，然后心里会有阴影再吧我感觉。然后会特别紧张，但是前面几次我确实我觉得自己的心态有问题，但是这次我觉得我心态没问题。我自己这么觉得的，我觉得我已经挺放松了，但是最后还是——不想要的结果，但是现在也不知道什么原因。搞不懂。

R：所以也是蛮折腾的。

P：对啊，你说虽然说也就做第一次试管，有些人可能说起来他们更惨两次三次，然后或者多次促排这样子，比起他们可能会好一点。但是想想嘛也是遭了这么多罪。有时候我甚至在想，还不如一开始不要成功，不成功，我说不定还可以有别的重新开始或者怎么样是吧？你这样这么折腾一下，等于说——因为我年纪不小了，明年就35了。我又得——等于说接下来半年，我除了做检查，别的事情又做不了。又耽误时间，耽误不起了，我觉得感觉是。如果说之前能说说自己怀，也不至于想着做试管，就是因为一年拖一年一年拖一年，感觉年龄越来越上去了，想想还是尝试一次试管，谁知道第一次就失败。（笑笑）

R:那你现在想的最多的是什么呢？

P:其实前两天很伤心，很难过。这两天——也没有理很好的思绪，然后就刚刚我在病房里跟隔壁床的一个在聊天，因为他做的是三代，他其实之前的经历跟我之前的经历有点类似。然后我就跟他聊了一下，我现在不知道我自己接下来应该怎么办？重新做检查呢还是说那天姜医生跟我说，他说建议我自己怀，他说不要去做试管，他说做试管的话就建议我做三代。所以我现在不知道自己怎么办，因为可能事情也在刚刚这两天，思绪有点乱。所以我也想跟他们聊聊看看，因为我就是想找这种经历差不多的人聊，看看他们是怎么在——比如说做哪些检查之类的。大家相互借鉴一下，然后再到时候再想想商量，商量接下来应该怎么弄，或者说比如说杭州有些检查做不了，要去上海或者怎么样，只能这样子。刚刚那个时候知道说说姜医生说不用保了什么，那个时候就感觉看不到希望，一而再再而三，心很累，真的很累。这么多年，其实在生孩子这条路上花的钱也就不说了，时间精力身体全垮了。也挺无奈。所以说那天姜医生跟我说，我就特别伤心，辛苦了这么久，到最后还是这样。差不多那天我就说，还不如一开始就不要成功就算了。还可以后面想想别的办法。

R:但你不尝试你也不知道。

P:对。这都是经历吧，也有可能就是每个人的命吧，你一次

R：那怎么安慰自己呢？

P：只能自己调节自己呗，你如果说自己再这样，再看看周边，说像我婆婆照顾我两个月都辛辛苦苦在这边，是吧？然后老公他们都挺了这么大的压力，自己再不调整好，再看看家里人，那就更加过意不去了。只能自己调节，先调节自己。

R:能跟我说说，你现在调整好了吗？

P:还没有这么快，我觉得还需要时间。

R:有没有给你带来一些稍微就偏积极一点的感受。

P:没有暂时。

R:对你的人际关系，包括家人同事，这些人际关系有没有什么影响？

P:做试管吗？

R：嗯，试管

P：人际关系，反正就是认识了一群就差不多经历中的姐妹啊，有些都反正聊得很好，就认识了这一群人。

R:那我觉得这也是积极的一方面。

P:对，其实有些因为大家聊起来了，其实有很多就是类似这种经历的，然后说起来就比较会有共同语言，因为经历差不多，对，然后大家的年龄阶段也差不多，然后会有很多的共同语言。还有一些经验，也值得大家相互参考参考，比如说去哪个医院检查检查，或者说有什么好的医生，比如做哪方面的检查，可以借鉴一下，参考一下这样。偶尔有时候觉得心情不好的时候，反而跟他们去交流，会觉得心情会豁然一点，不知道为什么，可能是病友的关系，

R:这方面有没有再稍微具体一点的？

P:暂时也没有，我觉得经历了一次，而且我觉得就像我开始说的比较顺。因为开始我们有八个人，就有一个小群，就很好的一起开始促排，经常返院聊天，经常在一起，到后面的话，因为大家每个身体状况不一样，都分开了，然后我呢又不喜欢去跟人家讲很多这方面的，如果人家主动要跟我聊，我就跟他说，不主动我就不太会去愿意多说，每个人经历也不一样，而且有些人未必会跟你说。所以后面就是，他们几个我是前面阶段接触的，所以说一直到现在他们有些在二促啊或者说准备第二次移植啊，然后我们都会聊得很多。然后后面其实接触的人也就是这段时间在医院住院，那其实很多是邵逸夫做试管的，然后大家会有一些共同语言会聊聊。但是相对而言还是跟那几个会聊的更多一点吧，因为这边都是时间很短的嘛，就病友吧，就几天出院了就这样。

R：那么前两天刚发现的时候，医生告诉你胎停不要保了的时候，你当时的心理过程能不能跟我说说？

P：很乱，我在姜医生面前哭了，当时我心很乱，真的很乱。其实，在我第二次B超就是星期天那天，B超出来结果我其实自己也知道了，然后我自己就给自己停药了，但是我婆婆他们的意思就是明天医生看了再说，因为那天是值班医生嘛，然后我自己给自己停药了，他们嘛还说第二天医生看了再说，那时候内心是崩溃的，真的崩溃的。

R：前一天就崩溃了？

P：对，前一天我就崩溃了，我其实前一天已经大哭过了。第二天姜医生，反正也就是很直接的跟我说了。反正其实虽然说前一天已经伤心过了，但是那天医生一说反正还是会崩溃一下，我觉得对我来说就是一个噩耗，挺难受。

R:想的最多的是什么呢？

P:辛苦了这么多，付出了这么多，为什么每次都是我？人家都是很顺利的，到了我这边就是怀个孕，怎么就这么难呢，一而再再而三的。可能经历的也太多了吧，就这么有点——在那一瞬间有点想不通，想不通，也不知道到底是——是我的身体出现了什么问题，还是到底是这个胚胎真的就是这么不坚强，百思不得其解。然后觉得有点说实话，有点对不起我老公。虽然说我老公老是在劝他说没孩子就没孩子就算了，但是不一样。有时候他说大不了领养一个或者怎么样，我说不要，我说自己能生一定要自己生。可以说传统观念吧，也可以说作为一个女人应该要有的，正常女人应该要有的，也有可能是为我老公考虑吧，我觉得他们家就一个儿子，我觉得我应该要为他生一个孩子。但是等到一次又一次的失败，真的是想想，这是为什么呢？R:确实有些东西科学都解释不了，自己怎么能想得通呢？

P:一次两次我也就认了，一而再再二三。

R:有没有想过以后的打算什么的？

P:以后打算嘛——唉还是得继续这条路啊，不然就只有不生的路！如果想生孩子，只能再是重新开始啊，该检查检查，吃药吃药！或者说其实我现在是想——想不好了已经。

R:有信心吗？

P:不大有信心，真的是不大有信心。

R:你觉得有哪些方面可以让你自己更有信心一点？

P:暂时，至少在最近这段时间我估计都不太会让——有什么事情会让我比较有信心吧。让我我看不到什么，就是等我怀孕哪次顺顺利利的这种希望我看不到，因为我不知道到底问题出在哪里，所以我现在还得等我胚胎染色体的报告，到底是我母体的关系还是胚胎的问题。

R：你前面没有做过

P：以前没有。那个时候我其实之前有看过何嘉玲，对，看了差不多两年吧。那次在他的调理之下怀了，但是也是没保住。也是50多天，然后他说建议我不要保了，因为HCG下降了。然后但是那次不巧，刚好他是小年夜那天告诉我，然后刚好夹在医院放假，所以没办法没得做。他本来说因为我是那个时候在三盛泰里那边看大的，然后他本来说让我来这边，然后小年夜这边也放假了。然后但是我的胚胎已经不行了，因为时间长不是要掉出来了嘛，我们绍兴本地又做不了检查，所以就没做。然后现在这次刚好在这边住院，我肯定是要做的。只不过我不知道就是试管的胚胎跟自己怀孕的胚胎是不是会有什么区别。被他筛选过的，跟自然怀的是不是会不会有不一样的，但是我们这种二代的应该也不太会怎么筛选，最多就是一个——

R：相对好一点。

P：所以到时候会不会有什么问题。所以我也不清楚，所以我等那边的报告出了，我才能决定我下一步应该怎么走。现在我真的是没想好。

R：反正，继续还是要继续走。

P：对，所以还是要继续走。具体怎么走呢现在还没有想好。不管说是试管呢，或者说——因为我是因为输卵管一只通而欠畅，所以导致自己难怀孕，就是把我的输卵管治疗好呢，还是说继续试管，所以我现在没想好。

R:能不能跟我说说移植就你的生育历程对你的生活都造成了哪些影响？能不能具体一点？

P:对生活？那除了工作——工作经济咯，就这两点咯，别的其实还好。其实因为我的收入本身就不高，但是我的收入——因为我们家房贷车贷都是我老公管的，我不管的。其实我自己的收入这几年基本上全是花在这个上面了。像我之前就是没怀孕，光光中药调理一年就要好几万，因为何嘉玲那里确实也贵（笑）。两年时间，然后每一次你说我这种每次怀孕都是保胎，住院，各种检查。包括就是那三次，前面自己怀孕的三次没成功，到每一次怀孕之前就是各种检查，基本上我现在这种常规的免疫啊就是能做的项目，基本上每年做一次。那经济肯定确实也是那个的，然后工作嘛——因为生孩子也是个大事情，这个事情一直没完成，工作肯定也是没有很好的心思放在工作上面，就是无心，一天到晚就想着自己这个事情，会分心。所以我现在找的工作都是很轻松的，文职类的。这样可能会——就一个压力会小一点，然后大部分的重心还是在自己这边，只能就收入低一点也没办法。这点么就是生育路的无奈——无奈也没办法。

R:对社交有影响吗？

P:社交其实我觉得还好吧，因为我，像这种事情也不太会去跟别人说，但是到了这个年龄吧，人家肯定会猜忌，这个年纪了，结婚这么多年了，为什么没孩子？唉，但是也不管他了，随便了。

R：你会在意吗？

P：会，但是——

R:会在人家说吗？

P:会啊，会在意人家说啊，但是现在也没办法，嘴长在人家身上（笑）。

R：人家会说你吗？

R:总归会有吧，我自己想想的，但是我也不确定。

P:有听到吗？

R:我自己没有亲耳听到，但是——因为我们现在跟婆婆是分开住的，但是我想想，因为没住在农村嘛，但是我想想他们会猜忌吧，因为他们，如果比如说我婆婆一段时间不在家里会呆我们这边来，然后他们村上的人会在他面前说你儿媳妇怀孕了还是怎么样，是会各种猜忌嘛。别的反正当面肯定不会在我面前说啊，这是一个很尴尬的话题啊。背后嘛我估计肯定会在说啊，只是没有亲耳听到过而已。

R:有别的压力吗？

P:别的压力就是来自父母，父母的压力。比如说——公公婆婆的压力会更大一点吧，他们之前，有时候婆婆来，比如说来我们家玩，我说你去小区楼下逛逛玩玩什么，然后他就会说人家——意思是人家就有小孩子的，他没有小孩子带着的，然后跟人家就没有共同话题。然后有时候会逼着我们去——赶紧去医院做检查干嘛干嘛的。然后——自己父母那边呢，可能比较会——不过这也是事实，会更加现实一点吧，会更加心疼一点我的身体。然后也可能会更加理解一点我，反正能怀孕能生是最好的。那公公婆婆嘛其实也能理解，就一个儿子，肯定是越早有后代，对他们来说是更好。然后虽然说他们有时候不说话吧，但是看着她们年纪也不轻了，都60多了，心里也挺——挺酸的。但是也是没有办法，我已经很尽力了（笑笑）。（半晌）很无奈。好在我老公还是很理解我。

R:老公——能说的具体一点吗？

P:他不会给我在生孩子这方面可以说是一点点压力吧！他甚至有时候经常会劝我算了，不要生了，两个人也挺好的。他说他从来不会说，把以后什么养老之类的会放在小孩子身上他说，他说趁着年轻自己多赚点钱，也有可能现在可能——现在也不是很年轻吧，这个年龄可能会没有想到很多，说不定等到哪一天老了会后悔吧，至少现在他觉得——因为本身他就不是很喜欢小孩子的那一类人，他觉得有个小孩子反而会觉得生活变得更乱，所以他觉得压力也会更大，他觉得没小孩反而好，潇洒现在这样。所以他有时候会劝我，他说没有就没有就算了他说，他有时候迫于父母的压力呢，他也不会来说我，但我自己听到我又不傻是吧，我只能去做，没有办法。所以我觉得这点我老公还算好吧！

R:那你自己的想法呢？

P:我自己肯定是想生啊。因为我说实话，我也不是很喜欢孩子，但是——自己的孩子一个我必须得要。也不是说——可以说传统观念吧，也可以说作为一个女人应该要有的，正常女人应该要有的，我是这么觉得。别的倒也没想这么多。也有可能是为我老公考虑吧，我觉得他们家就一个儿子，我觉得我应该要为他生一个孩子。所以反而是他劝我多吧。对，所以我有时候就是很极端，我前两天就在说——我说要不离婚吧，我说你再找一个吧，然后他就骂我，我说我再这样折腾下去，我说我说不定哪天就想放弃了，不想再去生孩子，真的很累，心很累。然后他反而会说我。但是有时候就是看着老人嘛特别心寒。老人嘛也能理解他们，都这把年纪了。

R:你自己有没有什么想对我说的？有没有什么补充？

P:自己也没有，我以为做试管毕竟胚胎是筛选过的，会和自然受孕的不一样（没那么容易流产），现在觉得试管的路也不是这么好走。真是不到万不得已还是不要走试管这条路吧，不是想象中这样的，吃尽苦头，用这么多激素。不是他们在说嘛什么掉头发什么，发胖什么的，虽然说我现在还没有这种症状，但是以后估计年纪大起来肯定是对身体是会有很大影响的。包括他这种促排针打起来这种卵巢变大什么的，虽然说一段时间就恢复了，但是我觉得以后可能是会有影响的。所以我挺佩服那种，我上次听说有一个她是第七次移植了，前面六次，对，然后我那时候我就在想，是什么让他有这种毅力，七次想都不敢想我。

R:我之前碰到一个八次的，而且都失败了。

P:他们靠的是什么信念在这样坚持的，我就有点想不通。可能他们内心比我们要强大很多吧！

R:你的内心也挺强大的。

P:我觉得我不够。在出事的那一瞬间是不够。（半晌）但是没办法，必须得坚强起来啊。但是有时候吧想的话肯定也是会想这个事情，不可能不想。毕竟是一个心病一样的这个东西，没有——比如就是一天没有生下一个健康的孩子，就这个坎永远都是存在的，永远就是一个很大的心病，很难剔除。特别到了过年的时候，回到农村或者亲戚朋友吃饭，人家带着孩子，然后或者亲戚家的孩子来玩，然后我们家没孩子，然后那种心情真的是挺难受的，说实话。真的很难受。毕竟结婚时间也长了，人家虽然说当面不说嘛，背后肯定在议论，现在挺没面子，所以我现在有时候——很多时候过年我挺害怕过年过节，逃避这种节假日，就是大家亲朋好友相聚的日子，因为他们会问，他说你现在什么情况啦，他们可能会知道，包括其实我现在这次是试管，我身边很多人我都没说，包括成功之后，我都没有告诉任何人，只有上班没办法，老板必须得说。然后很多人我都是不说，能不说我都尽量不说，因为我就是害怕出现像现在这样情况，到时就是空欢喜一场。不然如果说平时聚会什么碰到他们就会说你现在什么情况啦，像我这种情况是一言难尽，因为没什么好说的，也不愿意去跟人家多说，没意思，没什么好说的。

R:为什么？

P:跟他们说了又没有用，无济于事，反而能得到他们一句哦你好可怜，就这么一句话，反而觉得我心里会更难受吧，有时候不说吧他们不知道这个事情，反而也就这样子了。说出去无非就是得到人家那一点点的怜悯跟同情，没必要我觉得。我也不需要。我其实不是——我不需要的是这种怜悯同情。不是我想要那种，所以我不愿意去说这种东西。除非哪天怀着健康的孩子，我才会把这种事情跟他们说是吧，大家还能分享一点喜悦。报喜不报忧吧。因为说出去没什么用啊，他们也帮助不了你什么东西。

R：自己好朋友之间呢？

P：我这次只说了一个，但是到现在出现了这个情况，我也没有告诉他，我因为我不想去说了。我觉得也没什么好说。因为就像前面说的，跟他们讲了之后就是一丝丝的怜悯和同情，对我来说没必要，我也不需要。所以还不如不说。

R:别的都没有什么感受有没有没讲到的？

P:我只想了解一下，但这可能观点跟这个没有关系。
