# Supplementary material for: An insurmountable obstacle: Experiences of Chinese women undergoing in vitro fertilization
Source: PLoS One. 2024 Oct 7;19(10):e0311660. doi: 10.1371/journal.pone.0311660 (PMC11458033; doi:10.1371/journal.pone.0311660)
Supplement: S1 Data — (ZIP) [file pone.0311660.s001.zip › data/P11.docx]

R：可以说一说关于移植的心路历程吗？

P：心路历程——那我觉得真的是唉一说就感觉就是心酸史了，以前都没有觉得，因为以前没有移植前听别人说是做试管怎么怎么样，就是就看别人很辛苦的时候，那时候感受不到，然后这几年自己经历了以后，就觉得唉呀真的是太辛酸了，就是第一次还好你移植失败的时候还没什么感觉，第二次第三次你反复失败的时候，就是那种每一次看到希望，然后每一次又失望那种。你的心态就越来越不好越来越不好，到最后就是那种崩溃，焦虑，很复杂的那种心情，我觉得。

R：能不能说得详细一点，怎么复杂?

P：反正是从我第二次移植失败开始吧，就开始心理上就开始恐惧这件事情了。

R：恐惧什么呢，恐惧哪些方面？

P：比如说是再次移植会不会再次失败，就会每天就是在空闲的时候都会去想这些问题，反复的去想，就是会不会再失败，然后本身做试管，身体上也不是那么舒服的，因为你打针呀什么的移植呀这个过程其实也并不是那么舒服的，这过程就很煎熬，等待的过程也很煎熬。

R：等待结果?

P：对，就是每一次，比如说促排的时候，你要看你的卵泡够不够多，你能不能成功进入下一步，然后移植的时候你又在担心能不能移植成功，然后等成功呢你又在担心他能不能正常地成长啊那样子，反正就是每一步都在担心，就是这个过程很煎熬的，所以说你要是反复失败，你那个心理就——我都说不出来那种感受，反正就是，就是很焦虑那种，每天都在担心，包括我后面就是心态就不好了，就怀上了我的心态都不好，应该说怀上是一件开心的事情，移植成功，但是我移植成功后我还是很焦虑，就是因为前面几次失败的原因吧，就导致心理应该是有阴影的，就成功了，你也会觉得会不会还跟前面几次一样。

R：就怕失败是吧？

P：对对对，就是再失败那种，出现问题。所以说在我这次怀孕的时候，其实我的那个身体状况和心理状况其实就不是很好。就是这次移植成功以后就是心态不是很好，我每天在家就是——人家说不是怀孕以后要心态放好，要开心一点，想一点美好的事情啊。就根本没有办法做到那方面，就是每天在家里还是很焦虑，就是总是在我每天睡醒的第一件事情就是看一下床单有没有血，就那种生怕会出现意外那种，有时候上厕所的时候总是看一下马桶会不会有什么血啊那种，就很——就出现那种焦虑。

R：就时时刻刻都在焦虑，

P：对，就没法做到说是怀孕了还能放松心情。

R：就感觉一关一关的。

P：嗯，就是这样子。

R：那自己怎么调节呢？

P：调节不好，有时候你看为了这个事情我还去学心理学，我去学心理学，因为我知道我心态现在不好了，之前还去报了班学的心理学，我想呢这样子我能自己调节自己的心情嘛，说是那么说我也有去改变，就是有时候特别焦虑的时候就看看书呀追追剧啊那样子的，就是把那个时间排的满一点，就是不要让自己太空嘛太闲去想这个事情。

R：还上班吗？

P：现在不上班，就为了这个事情，已经不上班两年了，因为没法上班，你要是做试管经常跑医院，然后单位里肯定是不可以这样子的，就干脆辞职了。然后就辞职了，就干脆就是什么事都不干，就干这一件事情。

R：那做的牺牲也挺大的。

P:对就干这一件事情，结果这件事情还是反反复复干不好。

R：那是不是压力挺大的？

P:对，就是前几年的时候，整个人呢就是上班那个时候没有把这个事情当成一回事的时候，人整个状态还是比较好的，比较年轻的，然后就自从开始搞试管这件事这两年左右，首先心态也变了，然后身体也反复流产啊这样子，再加上人的焦虑啊什么的各个方面，然后我现在我的朋友见我就说，就感觉我整个人老了好多岁，就一下子变化很大。

R：我今天看你也挺憔悴的，

P：对就很憔悴，因为就每天那个身心疲惫你知道吗，就那种感觉。放松不下来，整个那个脑神经放松不下来的那种状态。

R：整天处于紧张的状态。

P：嗯。就是我朋友周边的朋友都每次在说，说感觉我这几年变化太大，就是没有像前几年看着那么有灵气了，我以前是很开朗的，然后这几年就感觉我心事重重的，就哪怕是朋友约我出去喝个茶，就坐在那里，他们看我的眼神都觉得——别人在跟我讲话，但是我的眼神是那种在想别的事儿，就是没有办法集中精神那种。

R：就心里想的都是这一块的事情。

P：对，就是这一块的事情。对，就因为这个事情导致你看，我跟我老公关系这几年也开始变得不好，主要是呃——好在是我老公他的性格比较好，他没有脾气，他还是比较包容我的，但是我因为这个事情这两年脾气就是变得特别暴躁那种，就很心烦，总想发火那种，就反正我觉得这件事情其实挺影响我的。

R：以前性格不会这样。

P:不是，我以前性格大大咧咧的，然后就是很开朗的那种，就是属于比较那种自由的那种，不会说是把一件事情太放在心上，可能也就是从我33岁开始吧，我准备计划要孩子开始，准备进入试管这条路的时候开始我整个人就变了。就第一次还好，也没太当回事也不是太懂可能。第二次开始我就有点变化了，一直到这一次我就更加那种，有点那种——就是有点那种绷不住了的那个心态。

R：这是第几次？

P: 是第三次了。第一次我是在泰国做的试管，嗯当时是不太懂，以为做完试管回来了以后，就跟正常的孕妇是一样的，当时也没有去做一些功课去了解试管是怎么样一个过程，然后你在国外做试管，它不像国内这个都是流水线的，国外的话就是那个啥移植完了你就可以走了，他后续不会交代你什么保胎啊用药，什么黄体酮，什么肝素，就没有这些了，然后所以我当时移植完以后，在泰国待了三天吧我就回来了，也没有用药，他那边不给你开后续的药，然后回来以后我就我也我也自己不知道，结果我当时其实还是挺幸运的，然后移了个龙凤胎，然后两个都着床了，其实发育的还挺好，我就那样没有用药的发育的还可以，前期我去那个社区的医院去做那个 B超呀抽血，看血的翻倍啊都是正常的，然后一直到7周的时候吧就胎停了。

R：前面激素翻倍都好的。

P:都好的。那有没有查过胚胎染色体？

P:做了三代。然后后来我去医院的时候就保胎来不及了，当时出血了，出血了以后就来不及了，就是去医院的时候有一个有一个胎停了，有一个就是胎心是微弱，后来医生就说可能不太能保得住了，到底什么原因，他说可能是因为缺黄体，也许是因为黄体。

R：你黄体前面很低吗？

P:我是按照正常孕妇的常规查的，之前是正常的呀，我在社区查正常的。

R：正常就不一定，医生也只是随便说了一个理由。

P:对啊我也不知道到底什么原因，反正中途也没有用过任何药，什么吃那些药啊什么都没有。但是医生的意思是说一般做试管的话都要用一些黄体支持啊什么的，我是什么都没有用，不知道也有没有关系，我也不清楚，反正我就胎停了。结果我又等了两年吧，两年又被疫情，我当时泰国去不了的话，其实那边还有一个胚胎的，去不了了然后我就想要在国内吧不是赶上疫情嘛出不去，然后我就在国内我就在邵逸夫做的试管。当时去做的时候，邵逸夫是给你做一代，因为你没有任何问题，我都——其实我跟我老公检查的那些都没有问题的。最后他们就说没有问题的话就是做一代。结果有2个鲜胚，2个那个囊胚吧，他当时第一次给我移了一个鲜的移了两个了，也是成功了，然后怀孕13周的时候，我去做nt的时候就发现有点那个不太好，然后当时他就说你再观察两周吧，两周以后差不多15周多了吧，然后去查就是那个多发畸形。其实全程都还发育的可以，翻倍啊什么都好的，结果是那个查出来是多发畸形，胎儿染色体有问题。

R：其实很多早期不好的大部分都是这个原因，尤其是你前面各种都长得挺好的，到后面突然间就不好。我觉得你不用去质疑这个用药那个用药。

P：对对对。第二次就是在邵逸夫16周的时候引产的。

R：那是蛮大的了

P：对对对就这次感觉就给我留下阴影了。就——因为16周时候我们那时候都有胎心啊什么的，都大了，就是感觉有感情了那种，不像是之前胚胎小流了也就流了，没什么感觉，反正大月份流产你就跟感觉跟胎儿已经建立感情了那种。然后那次流产其实——（被打扰了一下）

P：反正去年那次给我的那个啥，身体上伤害也挺大的，因为大月份流产嘛，心理上也是创伤挺大的，感觉一下子缓不过来了。

R：调整了多久？

P：一年吧

R：怎么调整呢？

P：就出去旅游呀，玩啊，就是去放松自己，我一般都是那样的，每次失败了，自己在家里啊就感觉这个坎过不去了，就得想想办法，然后我老公就带我出去旅游呀玩呀那样子，就尽量减少我一个人在家里的时间，因为我在家里老是胡思乱想，就避免不了这个问题。我是去年的5月份流产的，然后去年的11月份我又开始进入第二次，就是在邵逸夫的第二次试管，当时就是查染色体有问题，他就建议我做三代了。我从11月份就开始准备了。重新取卵，其实前面还有两个，我就暂时不用了，我害怕再出现问题，像染色体的问题。

R：噢他做了一代以后就不能做三代

P：对，一代转不了三代。最后就没办法，那两个就还存着的，然后就直接做三代了。做三代呢折腾来折腾去，从11月份折腾到今年3月底才移植的，本来我以为这次可能就不会再出现问题了吧，我想的是这一次我和我老公身体之前没查的能查的反正都查了，就挺全面的，免疫啊什么各个方面都查了。然后再加上这一次做的又是三代嘛，而且我这次移植的那个胚胎也还算是优胚，就是觉得应该是没有问题啊，不会再出现问题了，结果还是出现问题。结果是移植23天的时候就出血了，然后问医生，医生也给不了一个确切的原因。

R：现在呢好像就只能看到三代做了嘛总是，因为现在基因的水平目前只能到这，可能还有一些别的目前的技术查不出来的原因有可能也会。所以说起来嘛染色体这一块也筛查过了。

P：对，我就在想染色体也查过了，应该是胎儿是没有问题的。然后我这边身体也查过了，也没有什么大的问题，什么问题也没有，然后宫腔环境也查了，然后那个宫腔镜什么也做了，都是在达标的情况下移植的，为什么还会出现问题。

R：所以就比较不理解是吧？

P：对，我现在就是不敢，哎不敢再尝试了，我跟我老公就有点都计划不太想生了那种，要么就是看我们想，我们想要是实在不行的话，要么就代孕。

R：代孕中国合法吗？

P：是不合法的，但是没有办法呀

R：现在有资源可以代孕吗？

R：现在我是听好像社会上现在有很多代孕的，对，好像中国还挺多的。国外也有代孕的，但是我们也不可能跑那么远去，但是你说如果真的自己生不了，那你说怎么办？是不是。

R：无奈的时候也是多一个选择我觉得。

P：对，现在我就是年龄一天天这样子，我大了嘛，硬是从三十三四岁搞这个事情，搞到现在都37了，这个年龄越大，然后卵巢功能不就越来越不好了嘛。

R：现在卵巢功能查出来还好咯？

P：好着呢。很奇怪的，我以前做试管的时候，我的卵巢功能查是AMH是1.6，然后去年我做那个试管的时候，我吃那个 DHA，还有辅酶q10，还有那个就是怀孕的综合维生素啊什么的，就一起吃了三四个月吧，然后12月份查AMH值的时候，我竟然到2.4，它这个不是不可逆的吗？

R：噢那误差也是有的。一来嘛误差也是有的。二来嘛我们机体的有一些功能也是在处于一个波动的状态，不能说它是一成不变的嘛。

P：对我之前查他们说这个卵巢功能是不可逆的，后来我想到怎么是因为我吃药的原因吗还是怎么回事，怎么会变到2.4。

R：吃药这种不知道，说不清，按理说这些维生素吃吃影响也不大的。

P：对啊我之前查的时候医生说让我赶紧要吧1.6哦也不是说是卵巢特别好的那种，有一点下降了。然后这次查2.4，医生说你卵巢功能挺好呀，就让我很那个什么

R：那总归是好，你有可能之前那一次查不准，对吧？

R：躯体上有没有什么压力，就身体，关于你自己的身体。

P：有啊就感觉你心理状态不好的时候，身体也会受到连累的，就是你心态不好的时候，你就觉得什么事都不想干，你周围的任何事情对你来说都没有意义，你也不想去干也不感兴趣，然后身体就更加一样，就是每天都是能躺着绝对不做什么，就感觉整个人状态就是很懒散那种，提不起那个劲，就是那种。

R：我的意思是有没有身体方面的压力，好比说去做移植对你的身体的伤害方面的，疼痛方面的。

P：有，我第一次做的时候没什么感觉，可能我心态好吧，开开心心的，也没把这个当回事儿，就觉得没什么压力，等我第二次的时候我就感觉有一点了就觉得打针也疼，什么都觉得挺煎熬的吧，第三次的时候就更加有那种感觉，可能心态发生变化了，身体上的疼痛也更明显了，就包括我这次移植完不是要打那个肝素吗，我觉得好煎熬呀就是那种。

R：打肝素就觉得很煎熬。

P：对，去年移植的时候怀孕不是前期比较顺利嘛，所以我打肝素呀什么的，我都觉得时间过得很快，然后那时候我打了三个月嘛，我都觉得时间过得很快啊，就没有把这个事情太放在心里，也没觉得特别疼痛，然后这次移植的时候我就感觉——我才打了20多天肝素，就每一天都很煎熬，就觉得打针是一件特别痛苦的事情。

R：那有没有本身就是你的肚皮的状态也越来越差？

P：这些因素也是有也有，就是去年打的时候打了一个月吧我可能肚皮才发生那种淤青，很严重的一个淤青，这次感觉移植了打肝素，没几天我的肚皮就开始出现各种淤青，就感觉没有像以前那么耐受一样的，就是身体上也会觉得很那样，就承受不住那种疼痛了，很煎熬。

R：别的方面呢？

P：别的方面，那倒没什么感觉，因为都是在麻药的情况下也没什么感觉。

R：麻药对你有影响吗？

P：麻药倒也没有什么大影响，但是我现在就是感觉记忆力特别差，不知道是不是因为我现在因为焦虑睡眠不好，可能也有影响，导致我记忆力不好。其它好像也没有别的了。这几年总是失眠嘛，就是老是想这个事就会睡眠不好。

R：你可能就是压力大嘛，觉得主要有哪些方面的压力？

P：社会的压力啊，感觉你到这个年龄了还没生孩子，就总觉得别人会用异样的眼光。

R：那是你自己觉得?

P：可能吧，也可能因为周围有朋友嘛，朋友们都有孩子了，你出去聚会什么人家都在聊孩子，你一个人在那就很——就不知道该干点啥，就很失落，以前是没有这种感觉的。到了这个年龄，因为周围的都有孩子了，但你没有的时候，你就会觉得很失落，感觉干任何事情就像脱离了他们那个圈子一样。

R：就有那种社交隔离感，是不是？

P：对对对，你不管走到哪里，人家都问你孩子多大了，人家孩子都上学了，你就不知道该说什么，就这种我心中的压力，其实别人也没说你什么，但是无形中你就是心里有呀。

R：其实是自己给自己的压力。

P：对对对，还有就是比如说亲戚朋友啊什么的，家人啊总是催你，赶紧啊该要孩子了，年龄越来越大了，嘴上说说好像也无所谓，但是其实心理上已经有压力。

R：就是他们会把——就是有蛮多压力也是来自家庭的吗？

P：对也蛮多的，再加上我老公也是独生子，再加上我总觉得唉我们结婚这么多年了，还没给他生个孩子嗯，我觉得在这方面也好像觉得对不起我老公，也是一种压力。就总是我这边老出问题嘛，怀上就出问题好像就出问题，总觉得这方面也觉得有压力。

R：不一定啊但是你这边你不是也能怀的吗？他自己不好啊，为什么要把责任归结到你自己去。

P：总觉得要是他换个女人，可能就更——可能人家就很顺利吧，就偏偏到我这肯定不顺利，

R：那可能你换个男人也会顺利的。（哈哈）

P：反正就是来自自各方面的压力吧，可能是周围给我的压力比较大，因为我周围的所有朋友都有孩子了，人家都二胎三胎都开始了，像我这个年龄啊那孩子都挺大的了。

R：那你没有一个就是稍微跟你自己比较接近的这样的一个圈子吗？

P：有，我还有两个朋友也是跟我——比我小两岁吧，也是要不上孩子，结婚这么久，不过他们是有原因的，就比如说有一个是子宫内膜太厚了，还有一个是卵巢功能太低了，零点几的样子，就是卵巢储备功能不好，排卵还是咋回事，他们都是有这个原因，所以说怀不上一直在看，也在看医生。关于我这个问题，我就觉得就查了这么多年，也没有查出具体原因，每次怀就出问题这种。反正很焦虑。但跟他们有时候聊天的时候也会聊孩子这个方面的，总觉得要不上孩子是一个圈子里的，但是唉我感觉我经历的比他们太多了，感觉还是不是一个圈子的。

R：那你们也不聊平时？

P：呃——很少，因为我总觉得这个事情吧毕竟是私事，我不太想喜欢跟那个就是让周围的朋友知道得太多，因为我做试管没有告诉他们。因为我总觉得这个事情吧还不是那种能就是到处说的那种，总觉得这事情还是放在自己家里就是和自己老公知道就行了。就是包括父母我有时候都不想让他们知道，因为他们不太懂这方面。他们就会觉得——哎呀反正老人家嘛就想法特别多，我就不太愿意讲。就是我给我老公也说，不要给周围朋友到处乱说，也不要给家里父母说，因为他们不太懂这方面，我怕他们有什么别的想法，再加上他们老问你老问你，你也会有压力的对不对，就尽量就是这个事情我们俩知道就行。所以这几次都是我跟我老公知道。

R：那你这样的话，其实相当于你所有东西都自己去承担，自己都没有发泄的地方。

P：对对对——也没办法给别人去讲。

R：为什么

P：没法讲，他们也不理解的，你像生了孩子的那些人，你去跟她们聊这个她们更不能理解。就像她没有经历过你试管这个过程，你给她讲这个过程她体会不到的。

R：那你平时有抒发自己这一些情绪的地方吗？

P：没有，就是脾气变差了，总想发火，对我老公发火，因为我跟我老公我们俩在杭州生活，我们的父母都在老家呀，就平时我面对最多的就是他，然后总是把一些情绪上的那种就想发泄给他那种，所以这两年我们俩老吵架，好在他还不跟我计较，他也知道可能这几年因为这个事情我承受了很多压力。包括对朋友我现在也发生变化，因为我总觉得好像不自信了，以前我是在我们朋友中我是最自信的那个人，就因为这两年这个事情吧，没孩子这事感觉我在我朋友面前也不是特别自信。就有时候总是回避她们，有时候他们就要出去啊约出去吃饭啥，我有时候就是不太想去，因为我现在觉得一出去大家都在聊孩子，自己感觉参与不进去，就是慢慢慢慢就感觉不太想融到那种圈子里。

R：就是觉得自己跟他们不一样是不是？

P：对对对对就是那种，越来越不自信那种。就是以前我出去的时候就是能正视别人的眼光，就是感觉很自信的，现在有时候总是——就是逃避别人的那种眼光，总感觉不敢去正视别人，不知道自己哪里好像总觉得就是欠缺点啥那种感觉。——就越来越那样子。

R：这可能就是你自己觉得怀孕生孩子都是一个人比较正常的过程，一个完整的女人应该有的一个过程，然后就觉得自己好像不够完整一样。

P：对的，是的。

R：你人际关系上也就是这一点？

P：对我们现在没有别的事情，真的我家里其实就是我跟我老公吧我们其实过得还好，也没有什么别的压力。唯独就是在孩子这方面就是比较坎坷。

R：父母这方面的压力大吗？

P：也还好，不是特别大，他们越是不给我们压力我觉得我们压力——。

R：你自己不是独生子女？

P：我自己不是，我有弟弟，我弟弟他也生孩子了。

P：所以我更着急了。

R：更着急了？

R：对更着急，就是别人的事情会让我觉得有压力，就是他没生孩子之前我还觉得没什么，孩子生了以后我就压力更大。有时候看到别人就是朋友带出来的孩子什么的啊很可爱什么的，自己心理上总觉得就是——就很不舒服，有时候在家里就会反省，就总觉得——哎呀我这个年龄应该是有孩子的，就为什么人家有我就没有。

R：那跟平时跟病友也不交流吗？

P：不交流，包括在邵逸夫移植的时候不是有那个群嘛，他们说要拉那个群，我就不太想进去。就是唉我现在不善于交际可能。

R：感觉看你的性格音还是比较善于交际的。

P：我以前是很善于交际，我现在很不太愿意就是进什么群啊什么的那种。

R：就是有点逃避。

P：对很排斥，也不想听他们说那些，就很烦。

R：其实你们是有共同语言的。

P：呃——可能是，但是我现在就是很逃避这个问题，就不愿意听那些——就是群里面说这个问题，那个问题，就是我一看到那些我更焦虑了，所以我干脆不要看。因为我平时在家里的时候也是没事干，就查一些资料，下载那个什么试管婴儿呀，包括一些什么妈妈帮呀那些啊，就看一些人家什么试管移植后出现的一些各种问题怎么怎么样，我就越看越焦虑就是，总觉得那些事都会发生在我身上就那种感觉。

R：一开始就是刚刚开始移植的时候，跟现在的感觉比起来有没有什么一些变化？或者说你开始就是说你移植之前觉得移植应该是怎么样的一个过程或者什么样？

P:我没有想过，我现在就是每一次做试管，我就是抱着一副任人宰割的那种状态，那就随便吧。反正为了生孩子你想怎么折磨我就怎么折磨我的那种感觉。

R：我不是问这个，可能我没表达清楚，就是你前面对移植的理解，跟现在对移植理解是不是有不一样。

P：噢！就是在没做这个试管之前，我是把这个事情想得很简单。我总觉得——也没想那么多脑子很空的，也没有做什么准备，然后也就也就那么做了，现在就是做了几次试管之后，我就发现，其实——哎真的是没有那么简单，真的是没有那么简单，也不是你每一次都那么幸运，就这个过程可能每一步都会出现问题，可能我以前就是想的太简单了，总觉得按照他的步骤走，最后就会成功，但是后来才发现越做问题越多越做问题越多（苦笑），就是就像闯关一样的，可能你这一关过去，你下一关不一定能过去那种。现在我对试管这件事情就是——唉，就是那种说不出来的感觉。我所以我现在就是有时候看到人家说是就是那种次数很多，取卵取了次数很多，移了很多次的那种人，我就感觉我特别佩服，我都不知道她是怎么坚持下来。就是每一次这个过程啊真的太煎熬。

R：主要是心理的煎熬。

P：对，心理上的煎熬大过于身体上的。

P:首先我是那种心思很重的人，就是不是那种心大的人，我是做任何事情都比较认真的，就是包括以前我上班的时候工作的时候做任何事情都是很很认真，包括我家里的那些琐事，我也是很认真那种。所以我这种人可能就不像有些人有些东西可以放下，其实我自己也知道这方面的问题，也跟自己说是哎呀放轻松，有些事情你就先放下，但是放不下，就是那样子不知道咋办。包括现在就像你说的，以前我在上班的时候，在我的领域里，我觉得就是我做的比较优秀，所以我以前是很自信的一个人。自从把那件事情放弃以后，就像你说的，我把所有的寄托都放在这个事情的时候，这件事情老是做不好，我越来越自卑。

R：但就这件事情不是你所能努力的，你知道吗？你已经很努力的，你遵医嘱就可以了，你去找医生做医嘱就可以了，你的努力已经到了，然后你要去自己把自己去努力别的你自己能改变的东西，有些时候就是这样，你改变能改变的接受不能改变的，你把这个事情交给医生，交给专家，交给医学专家就够了，你自己身体去配合就可以了，但这是最最理性的一种想法。

P：其实我有时候也会自己一个人就是偶尔会冒出一个想法就是说，如果我这辈子就是比如说试管不成功，我要是生不了孩子，我如果这辈子没孩子，最差的这个结局我自己到底能不能接受？我感觉我接受不了，有时候突然冒出这个想法的时候，我就有点逃避害怕。还有一方面，有一方面是心理上的暗示，就是前两年忽然间朋友介绍了一个算命的一个道士，我就无意当中就想算一下嘛，哎呀我真的现在特别后悔，我当时不算就好了，算了以后真的是跟我说了，真的是说我没孩子，他说你老公还是有孩子，意思说我这边是没孩子。这句话说的我就开始就是这两年我就一直就心里有根刺，这换做以前对这个算命我根本就不会相信的，我觉得他们就是为了骗钱瞎说说的。但是他当你把这个话说出来以后，再加上这两年，真的感觉就像是没有成功啊怎么样，他当时就我说你这两年别要孩子，两年前啊他说你这两年不要孩子，就算要也不也不会好，叫我算了，他说让我在三十七八岁才要，然后就这句话当时说完以后，我就心里面就像埋下了伏笔一样。然后再加上我这几次老是失败，我就总是会联想他说的这句话，就有种心理上的那根刺。

P:我现在就是处于一种特别迷茫的状态，就是发现我的人生没有目标了，自从做这件事情以后，我就人生没有目标，然后对所有事情都已经不再关心了，就是之前什么疫情啊什么这些乱七八糟，好像对我来说我一点也不在乎，一点也不在意，感觉任何事情都跟我没有关系，就是每天就是——就是全在这了，就那种。因为我觉得我的人生就差这一步就完美了，嗯所以我就觉得就这一步为什么别为什么别人都能很轻松的过去，我就过不去了呢？就一个劲就在这在这努力。就人越来越不像样子，然后心理状态也不行。这几年什么都没干，就在干这个事，真的是很浪费时间。但是我现在也不敢再去干，就是分散——为了分散这个事情去做别的事情，因为我总觉得我的年龄到这了，我不能再耽误了。

R：那问题是你可以同时进行，你找一个自由一点的，你找一个好比说自己去建一个，建一个公众号或者建一个微博，然后定期的分享一下自己觉得有价值的东西，你可以去尝试这方面的我是觉得也能找到一些价值的，对不对？你也不是说为了赚多少钱，你也不差这个钱，你就去找一下你自己你自己的价值，然后也让自己少想一点这些事情嘛对不对？

P:可能还是觉得在这个还是觉得现在这个状态，这几年没有找到一些有共同话题的人吧，没有人能理解我那种。

R：其实你们蛮多人，就是有不少病友跟我反映了，就你们很多病友之间就是别人不理解，但是你们自己其实内部是可以理解的，就在这种你需要就是不是很表面的跟他们去交流那种，也没多少意思，你总会碰到可能有时候大家一看词只是分享一些知识，分享一些相关的，哈比如说哪个医生好一点对吧？哪种治疗方式那个一点分享一下这些信息，那么到后面慢慢聊的多了，可能也会分享一些情感，甚至在这个过程中你们还会建立深厚的友谊，也很也很难说的，就是表面上你不需要去抗拒它，因为表面上你要接受自己这个情况，然后要融入到这样的一个集体当中，可能会有不一样的收获。

P:嗯，可能是我——可能我以前吧就比较清高一点吧，可能把自己看的太那啥了。所以可能我现在进入这个圈子以后，我之所以抗拒，我总觉得我跟他们不一样那种，我就不想进到那个圈子里去，可能不太愿意接受现实吧那种。

R：总是想自己努力一下就能成功了，成功好了也就过去了。

P:对，我觉得我不应该属于这个圈子里的人。

R：还是否认的状态，

P：对，我不想进去跟他们讨论那么久那么长时间，我总觉得我自己努力了，按照医生说的也不过就是一个月两个月，可能我就脱离这个圈子这个群体了，对不对？我不想跟他们一直因为这个事情去交流啊什么的，可能也有这方面的原因。

R：调整一下吧试一试觉得

P:我之前倒是有一次就是第一次移植失败的时候，住院的时候倒是认识一个女孩子，她也是因为流产了，我们俩就加了个加了好友，然后当时我们俩确实是交流的还挺多的，因为都是流产，但后来没几个月她就怀孕了，哎呦现在孩子都很大了，后来也就不再交流了，后面就不知道了。

R：对你们其实涉及到的是这个问题，可能你交流的比较好，然后对方突然间就怀孕了，她就离开了这个问题。

P：对对对，然后我会更失落。

P：就觉得人家怎么那么幸运啊就那种，自己就越来越失落。反正是前几年的时候，在没有打算要孩子的时候，我真的是没有想到自己要孩子会这么困难。

R：这个哪里想得到。

P:对，因为我以前也怀过孕，怀过很多次，年轻的时候总是不要，而且特别容易怀，我是那种。然后那个时候无所谓，把这个事情看得好像很无所谓，再说年轻也不懂得保护自己，就不要就流产那样子，等到我真正说是想要孩子的时候，就发现怎么要孩子这么难。

R：也是这个老公的？

P：噢之前不是，那时候年轻嘛谈男朋友，20来岁的时候。

R：有点遗憾，但是很难说，我是觉得你现在也不是说怀不上，你是怀上的呀，因为该查的也都查了，你也是怀上突然间就不好，这种原因，其实很多本身就胚胎有问题，然后现有的技术可能又查不出这个问题，这也是有可能的。你们老公染色体查过吗？

P：查过，都查过。我们俩都是没什么问题。

之后闲聊了一些时间，研究者并通过这次机会给予受访者一些心理疏通和安慰。
