# Supplementary material for: An insurmountable obstacle: Experiences of Chinese women undergoing in vitro fertilization
Source: PLoS One. 2024 Oct 7;19(10):e0311660. doi: 10.1371/journal.pone.0311660 (PMC11458033; doi:10.1371/journal.pone.0311660)
Supplement: S1 Data — (ZIP) [file pone.0311660.s001.zip › data/P7+P8.docx]

R：就是想知道你们这一路过来，就在生孩子这件事情上应该也是蛮吃力的，所以想知道你们这一路过来都是怎么样的一个感受心路历程。

P(7)：心路历程。。。

R：对对对

P(7)：太艰辛哦，金华那边第一次做试管，第一次做成功虽然是成功了，移植的两个都成了。但是在两个月的时候一个停胎了，另外一个到五个月左右的时候，羊水早破，但是当时他们也没有给我查具体的原因，只是说感染。但是感染他也没有去——找出具体的原因。

R：染色体也没查？

P(7)：染色体查了是好的，按道理五个月么应该染色体是没什么问题。

R：五个月那是挺大的了，那是挺伤心的，我觉得。

P(7)：因为那时候也是，就跟生小孩一样的，晚上也睡不好，白天也睡不好，就是那种。

R：自己生下来的？

P(7)：嗯，生下来我自己是没看，我老公是看了，因为不敢看（苦笑）。当时是先破水了嘛，医院意思是你如果羊水没了，你到时候就算保胎生下来他也怕你不是手指会粘连，他说，你到时候生下来你到时候更加痛苦一点。因为里面没有水容易说粘连，医生是这么讲的。那时候保胎也保了一个多月吧在医院里。还是没有保下来。

R：当时最深的感受是什么呢？

P(7)：压力，心理的压力应该是。对，生下来以后还——不是那时候，是因为黄体酮打多了还是什么，反正胎盘都是没生下来了。所以都是人工——靠人工这样剥下来的。所以我第一次做宫腔镜是，这边邵逸夫做的是重度的。

R：重度粘连？

P(7)：自己感觉——内裤上排出来的那种黏糊糊的。打了三个月的黄体酮，打了太久。

R：这次事件自己心里的感触是怎么样的？当时是怎么想的？

P(7)：想——好不容易才有，又——又失去了（苦笑）。所以有时候都不是太想去想那些事情（苦笑）。（停顿）

R：那当时后来是怎么一步一步调整过来的？

P(7)：家里人啊开导啊主要是，医生也讲，他说也有可能他自己自然就怀孕啦，因为说流产过以后，它就是什么激素高的话容易自然怀孕。但是像我这种粘连严重的也不太会怀。所以我一直拖。不是粘连吗？我宫腔镜就做了四次还是五次总共。6月份做的这一次是好的，好像4月份那一次做的还有一个角是粘连，今年6月份做那次是好的。所以过了这么久嘛也——粘连不处理好，我想再做可能也会影响这一次的试管。那时候我跟我老公两个人都——（苦笑），他吧就是不在我面前表现出来，（停顿）因为如果再过两个月的话，

可能保胎就早产的话，可能会有希望了。因为那时候五个月时候，大脑都没发育好，医生也不建议保胎。今天那个姜医生来查房她说我血里面还是有感染。她说如果不看好的话，还会跟前一次一样。血常规我自己看了，也没看出什么，今天姜主任看了，她说血里面白细胞偏高，尿是好的，因为我尿路也感染过了。明天说再复查一个，如果白细胞还是偏高的话，她说肯定要治疗。（停顿）

R：那么那次事件就是当时那个事情，给家里人，还有身边的朋友有没有带来了哪些影响，包括你自己？

P(7)：肯定都会有的。只是家里人也都不在我面前表现出来。

R：都不在你面前表现，那表面上他们也都是在安慰你的？

P(7)：对。毕竟自己想想年纪也有了。16年的话也有35虚岁了。主要以前年轻不想要，流产，自己去流掉。有一个是四个月的时候再去流掉，再后来一直自然就怀不上。自然怀孕很难。（停顿）

R：这一路过来有没有感觉到周围一些人际关系的一些变化什么的？这些方面有没有给你带来什么影响？

P(7)：这倒没有。主要是说工作方面啊，不过我工作方面还好，毕竟也还比较自由。

R：个体的是吧？

P(7)：对。工作上还没什么。

R：那么做移植这一路过来不也是蛮繁琐的吗？有没有对你日常生活造成一些影响什么的？

P(7)：主要是有时候赶早班车到这边来也挺累的，来来回回，但是嘛又不想住在外面一个人，感觉还是要想回家（笑）。住在外面那边也挺便宜的他们，一百多一天连吃饭都算的。但是我来回我车费就要150，还其他不算。

R：那你就每次都这样车来车回的自己开车？

P(7)：坐高铁。金华那边近，我十来分钟转个地铁。R：然后当天早上来这样子的？

P(7)：嗯，都赶最早那一趟6:42那一趟。家里六点钟出门，5点半起床，有时候，有时候处理结果等一下就要到下午。我特别害怕周五那一天，因为周五那天不是都要放假，所以回去很不方便。票有时候不知道买到几点钟。

R：那是挺不方便的哦。

P(7)：有时候在那里等，只能坐在那里等，等结果。唉其实做个试管的，压力也挺大的。

R：具体都有哪些呢？

P(7)：你促排的时候你又怕卵泡，不知道长不长得好。长起来了，又不知道他取能取几个。取出来你又想着配能配几个，配的他不是都移冻胚嘛，所以又怕他解冻把你解坏了，虽然说少，但是还是会想。因为我本来就卵泡少。这次就取了三个，配成两个。再配好么移植，移植进去嘛又担心他不着床。我移植在邵逸夫是第一次，之前是金华，金华还有两个胚胎，

没去移。

R：金华你那边就移了一次吗？

P(7)：嗯

R：那成功率还高的。

P(7)：那时候内膜都好的，什么都好的，就是感染他们没查。感染源没查。其实都有炎症，肯定体内，他们没给用药。

R：这种也很难说。

P(7)：所以现在宫腔镜做多了嘛，内膜变成不是那么光，宫腔镜查出来是凹凸不平，做B超也每次都讲凹凸不平。它不是那个多了嘛，粘连也多了（停顿），再我的宫角也比较小，只能怀一个。

R：还有别的什么感慨吗？

P(7)：一下子也想不出来

R：你能不能告诉我，在你上一次的流产后，到后来又重新决定再去做移植这一段时间，你都是一步一步怎么走过来的，然后你自己的内心是怎么样去调整过来的？就一开始是怎么想的，后面是怎么想的？

P(7)：主要是——我不是那时候住家里嘛，在义乌上班，所以每天来回啊，有时候就忘了这个事情。因为早上出去，下午又回来这样子。因为路上来回就要一个半小时两个小时慢的话。

R：那你路上总也会想的吧？

P(7)：还好吧，主要是刚开始那段时间，朋友来啦，亲戚来啦，都——不想去想那件事情，他们也不会说来问啊什么的。（停顿）

R：就是都不想说。

P(7)：嗯，感觉太难接受了，毕竟这么大。五个月，那五个月的话就是手啊脚长得差不多，就是器官还没发育好。所以当时流掉的时候我都不敢去看。（停顿）

R：那么你觉得你现在接受了吗？

P(7)：现在我不去想嘛还好，毕竟时间也有段时间了，你去想嘛想想还是很心疼的。那时候半夜经常在被窝里哭（苦笑）。想起来记忆也是很痛苦的。因为我们这个年纪了啦，还没生主要是，一胎都还没有。如果有个一胎啊，你又会感觉反正有一个也就算了。主要年纪在这里。

R：那你平时都是怎么安慰自己的呢？

P(7)：我都自己都忘了，这么久。那时候只能要么——也不能在家里人面前表现得太怎么样，这样大家都感觉痛苦。（停顿）主要是家里人都还好。

R：怎么还好？

P(7)：不会说呃——不会给压力这些都不会。（停顿）

R：就都不在你面前说的。

P(7)：嗯——（停顿）

R：那还挺好。

P(7)：所以家里人啊也很重要。（停顿）他们会说，肯定还会有的。（停顿）

R：那我们希望还是要有希望的。

P(7)：就是像我们比起有些人来说，还是算幸运的。像有些的真的，做试管的做不了那种真的才叫痛苦。我发现他们那种做三代试管的比我们这种还要复杂。取卵取出来嘞，胚好还要养囊，养囊也不知道几个能养成。养起来吧检测也不知道能不能检测通过。检测通过了他还是会有几率染色体异常的。像我们两个人染色体都好的，也是会说有出现有这样的概率和染色体异常。(停顿)

R：那么这次移植以后感受怎么样？

P(7)：心里也还挺紧张。明天又要抽血了，不知道情况怎么样？

R：今天晚上会特别紧张是吧？

P(8)：肯定好的，你放心好了。

P(7)：我（笑），我自己都不敢测。我听他们讲讲么，在这里的，他们基本上都是成功的！所以我都不敢回家，移植本来说住在那种民宿里面，我又担心怕他不着床啊。唉，因为他们说你们这里保胎还好。

R：压力还是大的哦。

P(7)：嗯，就感觉不敢面对现实一样，自己都不敢。。。。。

R：这都很正常！

P(7)：医生说头也不能洗，我还真的没洗，就今天跟那个跑下去洗了一下，我想想反正都八天了嘛也差不多了吧。（停顿）

R：那么这些经历有没有说给你带来一些正面一点的影响，比如说有没有让你的内心更加坚强一点，这一类的。

P(7)：嗯——差不多吧，反正很纠结很复杂，天天在群里吸取他们的成功经验（开心的笑着）。

R：群看的越多越会胡思乱想了吧！

P(7)：我这个大群也不太看。小群呢她们会讲怎么样。

R：那我觉得像你们这样移植的有一个小群体，大家相互关心也蛮好的。

P(7)：前面两个都是在你们这里保胎的。他们两个成功了出院了，一超都过了，韩丽芳，她说住在24床。再还有一个我前面，住在我这个床前面的，叫什么名字我都没问过。反正也还小吧，她比我们小，年纪比我们小。另外那个是二胎的，原来有个儿子天天担心，不是现在怀了双胞胎，担心是儿子还女儿，呵呵我们说肯定两个女儿，反正她儿子以前不孕吐，现在很会吐。

R：呵呵这种很难说的，我们之前有一个病人住在这里的，然后也是这样嘛前面一个儿子，后来又怀了个双胞胎，后来生出来两个儿子，变成三个儿子，要崩溃了。

P(7)：对，他们都在考虑说换房子换车子换成商务车。

R：是的，一家人旅游都出不去了

P：七座的刚好，一辆车都坐不下。她说她婆婆不来带，两个人。

R：哦那很吃力的。

P(7)：就是前两天在邵逸夫那边做B超了，医生问要不要减胎，她说不减，B超做起来那么好，她说为什么要减？

R：那是可惜的，肯定也不舍得。

P(7)：不是也会有影响，风险还是有的。减胎了以后还要来保胎，有很多人也放不住。（停顿）

（7给我的感觉始终没有很正面地谈论自己的内心感受，可能是太害怕想起而不愿意多说，也可能是碍于大家都不是太熟，加上边上还有8在。我见7又停顿半晌，时间也过去了大半，便转向问8病友）

R：那么XXX呢？

P(8)：我其实我真的心态还算好的。因为是这样子的，我妹妹她已经生了两个小孩子了，还有我周围的有的小孩子就生了嘛就不太听话，你知道吗？因为真的很生气很生气的，然后出去说我那些朋友就有点潜移默化的就说那算了，你看现在小孩子都靠不住的，反正都这样子说嘛，其实我知道也是安慰我嘛，因为小孩子大了她还是会听话，只是现在可能是在叛逆期，就是说现在只是刚好在叛逆期嘛。我本来刚开始第一次做的时候是这样子，自己不太懂，去也是邵逸夫移植到，然后邵逸夫移植第一次去14天的时候抽血值的时候，去的太晚了，那个时候确实知识，所以我说知识还是很重要，那时候真的试管婴儿的知识那时候我真的没怎么去看，天天都上班上班，还上班啊没休息。

P(7)：所以还是群里经验还是得吸取一下。

P(8)：嗯，还是有影响，因为啥，你说你上班再怎么没压力，你总要想着是上班的事情，然后吃也没有说你在家里天天吃的那么稍微好一点点，所以我第一次去14天才去抽血，抽完血值就很低，医生就说血值有点低，你要去中医院保胎。那我当时以为中医院保胎就是开中药了，就门诊开了中药就拿回去吃了，药也没有，反正就原来的药又没加又没减，反正就那些药就一直，该打黄体酮的打黄体酮，吃吃就是这些药，什么保胎丸呐这些都没有的。回来吃了结果就等那个是28天一超嘛，第一次去是没有——没有胎心胎芽的，然后就是血值也很低，那那个邵逸夫的医生就说了，他说你这个没看到胎心胎芽，他说可能胎停的几率比较大嘛，那我说那再等等可不可以我说我本来体质比较差。然后他说要么就再等一个星期，那我就再等一个星期。那一个星期真的是煎熬，然后说我真的，我妈上来，那我又不好意思就是说很难过啊表现出来。其实心里面真的挺煎熬的，那个星期就没上班了。就在那里等等等。再就是等到后面再去查的时候确实是没有胎心胎芽。后面就直接就——先吃的药，吃完药以后，后来

后面仍然没胎芽就流掉了就，就还是清宫了一下，没有流干净，对，清宫了一下。就这样子之后。。。

P(7)：这样子还算可以，不算很大。

P(8)：就是还能药流。然后没看到嘛也就算了。真的，那个时候真的不懂，你看这一次我真的不一样，这次我自己就很多东西我都知道，所以说反正该用的药就拼命的用，反正你前面都花这么多钱了，后面真的没关系的，就是该用的药就用上去，我其实有时候那些东西也看不懂，反正就是一出结果看，比如血没涨，就赶紧百度一下或者群里面问一下怎么血没涨，吃哪种药会让血值涨，因为前期血值还不错嘛，后面没涨就觉得不知道是哪里出问题。就是，反正就是该吃药就吃该加就加。第一次还是后面打掉了以后还是挺，挺折磨人的，后面就，想想就算了算了，就顺其自然吧，然后慢慢再来弄。然后那个时候真是又要买房子，所以说可能心思在买房子上面去了，真的，不然没有买房子的话，可能你一直那样子，心里也难过的。心思一直就是买房子买房子，然后又去上班去了，没钱啊，又去上班去了，休息没多久就去上班，上完班以后，唉后面就是房子也有了，然后一个也两个人过得也差不多，也就两个人过过这多舒服啊日子，真是后面想想看的还是说要不就再去试一下。你能有就有嘛，实在是没有，那也就没关系了啦。

R：那你也是这种心态。

P(8)：对，反正我想想，就是你肯定要努力一次，肯定要努力，你不要说一点都没有去努力，然后就说算了算了你一个人过得挺好，你们两个过得好，就不管什么事情了。因为我心里面就一直想的是反正就是过呗，如果说实在是不行的话，我妹妹她们两个小孩子反正到时候她不可能不养你吧，她不可能，你有钱的话，她还是会养你的，然后他那两个稍微还还，目前看起来比较懂事一点，好像不会说不养吧，就是这种心态。反正就是如果没小孩嘛肯定说心里话，内心是有很大的一个遗憾。但是如果说确实是真的没有嘛，主要就是说缘分的。。。

P(7)：像我们其实做试管还是能做成的，你一定要努力。像他们你说做试管都做不成了，那样子真的是没办法靠自己努力，想想自己还算是幸运的。

P(8): 有时候我其实我跟你说说心里话也在想。你看一下，你有时候幸好我们还这里住院几百块钱一天，你看他们外卖小哥，有的你说都是残疾人的，都还是在挣那个钱几块几块的挣。不能就是说老是跟好的比，他们儿女双全的，家里有车有房跟那样子的比。那样子是他们一开始就是说像我的话，就是说确实是牺牲了就是说生小孩子的时间，然后去工作啊挣钱去。我这个确实是我自己最早的时候把好的时间就是说。。。

P(7)：对我那时候也是，没有早点去做试管，从来没想过，

只想着天天上班挣钱那时候。

P(8)：真的有时候就感触这方面感触会特别大。

P(7)：那时候最早的一次感触就是，我那时真的还很小，十几岁，住在别人家里，那个小孩子半夜嗷嗷在哪里哭，哭得我们，对，所以从那时开始我对小孩子有点反感，所以一直我们到30岁才结婚。

P(8)：我觉得我也比较晚，27岁才结婚。实在是家人催的没办法才结婚。

P(7)：催催才催婚的，我老公嘛也喜欢玩那种，所以两个人都没想到结婚，一直等到两个人30岁，谈了12年的恋爱了时候才想到结婚。

R：哇谈了12年啦！所以现在这方面感触是不是特别深？

P(7)：那时候年轻的时候根本就不想要小孩，就觉得小孩子好烦。不自由啊，你一个小孩子走到哪里都感觉不方便，所以那时候也根本没想过要小孩子。

P(8)：我其实是想到要小孩子，我就是觉得没钱什么的。没钱没房子，然后就觉得哎呀生下来我怎么养，租的房子什么的，怕给到他的那个条件不是太好。就这种想法，想东想西的。不过我是这样的，我是第一次第一次很小了，然后就去——就，谈了第一个朋友，高中的时候就谈的第一个朋友，胎就打了，然后打了呢然后后面可能就是没养好嘛，然后第二次就跟我老公在一起嘛，我后面我也谈过朋友的就是没有怀孕。然后可能就是，好像后面就是说我子宫有点靠后吧还是怎么样？反正就一直都没怀孕了，没避孕然后也没怀孕。

P(8)：子宫后位好像是不是说不大会容易怀孕的？

R：这个倒没说，只是说子宫后位可能有些时候是因为盆腔的炎症引起的韧带后拉啦什么的，所以一般的话前位肯定好一点的。

P(8):然后后面那个就是跟我这个老公在一起的时候啦，然后后来那次刚好两个人回去结婚，两个人回去结婚，然后那个我月经本来就不准的，然后那天还带他爸爸去我们那边玩嘛山上玩，就来了一点血，我就以为是身上来了，推迟了好几天了嘛，那我就以为是身上来了，一丁点丁点的，我想刚好前期的那种症状就一点点。

P(7)：流产的征兆了

P(8)：不是，宫外孕，大出血，然后很吓人的，因为不知道当时那天就痛得真的是不知道，因为我自己不知道是怀孕的，如果知道的话那肯定就是有那种想法。我就觉得是胃痛，其实根本就不是胃痛。然后血已经崩了很多了，我好像输了有六千，六千毫升血，反正是这样的他们，对，然后。。

R：那是相当厉害了，体内的血都换了。

P(8)：对，反正就相当于换了以后，然后我就那个，就去医院，然后所以说我的那个时候是在县城里面的，然后那个时候没有那种，那只能开刀了，然后县城里面医院的医生都技术比较差了，就不是那种像我小姊妹她们是生小孩子开刀是这样横切的，然后后面就是一条印嘛就像胖了一样，你看我是很黑黑的一条那种原来老的那种，就是说必须要开刀了，然后你再不这样人都保不住了，然后我妈吓死了，赶紧的就开刀。

P(7)：还管他怎么切嘞

P(8)：对啊那个时候就说保命要紧，对，那这输卵管一侧右边就已经没用了，然后后面就是说一直再怀，都一直再没怀上。

P(7)：那你单侧也不容易怀

P(8)：怀不了，那边已经积水了。不是，左边也积水了，然后根本就怀不了，其实我如果左边到时候你怀上了的话又是宫外孕是吧？后面就去想，然后就说去做试管吧，做试管医生还叫我那个邵逸夫医生还说，他说要不那个积水给你抽抽，你再试半年，然后怀上了然后就不做试管了。诶我说那不用了，我说我积水我自己知道，这么多年下来都没怀，哪里再过半年能怀吗是不是？

R：不一定的他如果说手术有做掉那也难说。

P(8)：后面我就直接说做试管了

P(7)：主要是想想感觉怀还要再等半年，半年没怀你还是要走这一条路。

P(8)：对，所以我是这样想的，我说还是做试管，做试管当时所以说还是说不懂这一点，我左边积水就是直接用管抽掉了，然后就没有去结扎，也没有切除掉，没结扎主要是。那我也没有做宫腹腔镜没做的，就直接就是移植了，移植了然后后面不是就胎停了，胎停后面查到原因就是说我左边的输卵管积水又倒回去了，然后所以说胚胎停育了。他说是这样说，我不知道是不是这样。

P(7)：他们说会把他淹死。

P(8)：对，左边的积水又回到宫腔里了，然后就不行了，然后就这样子，后面就去邵逸夫再做那个宫腹腔镜，他说做一下吧，宫腹腔镜做了嘛就是说把里面的脏的，反正你也刚打完小孩，里面肯定很多粘连肯定有的。然后就做了个宫腔镜，然后就又休息了一下。然后再又开始又做。就宫腹腔镜做了后面就是，在那之前那个张嵩英他又说，他说要不，就是我移植前吧，就1月份，相当于又叫我做了一个的，相当于做了两个（宫腔镜）。他说你再做一个吧，他说宫腔镜做一下的话，就是说给你把那些脏的给你拿拿出来，然后给你里面好像就是会，或者是土地会松一点吧，好一点。

P(7):主要是我只有两个胚胎，移了就没有了。

R：就是这次这两个是吧？

P(7):对，不成又要重新取卵，重新开始——其实也挺那个的。

R：那么后面是怎么调整过来的？

P(8):后面我吃中药啊

R:我的意思是心理过程，其实因为我做的课题是关于心理这块，所以我就特别关心这点

P：心理过程我就是全部都寄托于工作，所以说这一点我觉得我们女的就真的是这样子的，一定要寄托于工作。（比较大声）

R：就是把注意力转移掉了是吧

P(8)：跟你讲，你不然的话你对公公婆婆对爸爸妈妈，你爸爸妈妈嘛他不来说你，但是看着你，她们总担心你了，都是都快40了你还一个小孩子都没有，你怎么怎么样，他嘴巴是不说的，但是心里——

P(7)：这样子是算好的

P(8)：所以一定一定就碰到这种事情的时候一定要投入到工作里面去，绝对不能在家里玩或者怎么样子

P(7)：越在家里也越胡思乱想。

P(8):一定要自己找事情做，然后你越是在家里你想一下钱也没有，你说拿你老公一份钱，一天到晚的，你问他要也不对是不是？然后你自己然后朋友圈也没有，你小姐妹到大家都忙，你一个月能休息几天，还有小孩子要照顾，像我们外地没几个朋友。所以说一定要工作，这一点我觉得挺重要。我真的就是如果说真的有不好的，马上就投入工作，但是一边工作一边要调理身体，身体是怎么样的调理，中药要吃的，健身，一定要健身，不健身的话就跑步。这些一定要做。

R：都有感悟了

P(7)：从那以后我就感觉我整个人都很虚，身上的斑斑点点都长出来。

R：这样子啊，以前没有的啊

P(7)：没有的。就那种什么釉一样的那种

R：疣，免疫力下来了。

P(8)：我是可能雌激素偏低的这些。这个就是后面出来的。

R：相关的药用得多了可能多多少少也会有点副作用的。

P(7)：促排的针打多了这些

R：那么还有没有给家人朋友带来什么影响？

P(8)：我跟我同事他们是没有说

R：为什么不说？

P(8)：诶我觉得就是说，本来自己压力也是大的，在做试管嘛。。。

P(7)：主要是你也不太愿意去想起这事什么的

P(8)：然后同事嘛又不比家里人是真的关心你对吧，同事的

话，有时候说你没成功啊他会说，诶你看年轻的时候玩吧，这时候怀不上来（笑了）。就是会有这样的担心人家说，可能其实现在有时候人家也没那么多时间来说你的，就自己会这样子去想

R：就是你自己的想法对吗？有没有说听到人家这么说了？

P(8)：我——因为我没说嘛，我没跟他们说，像我这次我就是说我回老家了

R：那朋友呢

P(8)：朋友说的，好的那几个肯定跟他们讲，那他们也知道就是说也都会叫我怎么样的调理啊什么的。

R：那家里人呢。

P(8):我觉得我们家里人还挺好的，其实像我们真的如果说你做试管，就家里人再不支持的话很难坚持得下去，就是像现在我第一次做就是说确实是没钱，然后还真的挺累，然后我也考虑钱，我老公也考虑钱。现在嘛满足的就是说，就是能够承受了。就觉得会轻松一点，就首先不会去考虑钱，其次的话就是年龄在这里，心态也变好了。有就有吧，没有就命里注定的就是这么想。你只能这样子想，因为，只能接受嘛，这种事情就只能接受。看高兴接受还是不高兴接受，我就是上次我宫外孕真的是差点没命的，第一次输了3000血，然后三天没醒过来，然后医生——要不是他们现在你也不知道你到底怎么没醒过来什么的，后面又输了3000血，然后我就第二天就醒过来了。还是血流的太多了，你想输6000啊，我说吓都吓死了。

R：捡回来一条命。

P(8)：真的，我说那个时候真的是我妈她是吓死了，因为那个时候刚好在家里，我妈是吓都吓死了，怎么会就是说手术做完了三天没醒过来，就是迷迷糊糊的，就是叫我是睁得开眼睛，我是全身无力。

R：比起那样，总归是现在怎么样都好。

P(7)：我对那个麻药很敏感

R：为什么对麻药很敏感？身体蛮敏感，还是心理敏感？

P(7)：身体，就是头晕，想吐。就是躺在床上，一动不动没事。你要起来转个身，反正他就要吐药，恶心。主要是取卵的事没关系，取卵因为时间短可能就几分钟。做宫腹腔镜难受。我看他们有些人一点事都没有，嘻嘻哈哈，我都睡在那里，醒来我就叫医生那个袋子拿来，因为突然恶心反胃。头痛，头晕。所以我想到宫腔镜我都很害怕。

R：这些经历有没有——有没有让你感觉到周围人际关系的一些改变什么的？

P(8)：这些——我觉得应该影响不是太大，我觉得可能大部分影响都不是很大，因为毕竟你生活的话是在杭州这样子的大城市里面，没有几个人就是说会去关注你有没有小孩啊，两口子怎么过，我觉得没有的。

R：那对你的日常生活呢？

P(8):日常生活——也还好可能工作太忙了没有时间去想到这些东西，有时候路上看到小孩子也觉得挺可爱，但也没有说很迫切的就是赶紧去弄一个什么的。要是肯定是想要的，就是到这个年纪了，就觉得顺其自然吧。因为看的太多，看太多了。像小孩子像家里面经常都是两口子离婚了，小孩子就放老家没人带的，然后一天到晚的看着也挺可怜的，所以有时候就会有这种一定要就是说父母双全的啊反正就是好好过啊，然后基本上最起码给他最基本的生活要好嘛对吧。是这样子，对周围的人际关系，我个人觉得就是应该没什么影响。

R：你这边有影响吗？

P(7)：没有

R：也没有的

P(8):可能也比较坚强，像亲戚朋友有时候说的话真的是关他什么事哦？

R：哈哈心态能这样挺好的。

P(8)：是啊，你有亲戚朋友说的话，你就脸马上拉下来就不高兴，你这个人就不要问了，就不会说了，也不会问了。

P(7)：但好像很少会说在很多人面前说，因为毕竟这不是说一件很好的事情。

R：大家基本上也都还那个的

P(7)：基本上不大会在你面前来讲。

R：那有没有说听到一些闲言碎语这种？

P(7)：因为我们没有生活在农村里嘛所以也听不太到，因为城市里嘛反正一关门谁都不认识谁。

R：有没有什么要补充的？

P(8)：你们这里的护士都挺好的。

R：那有些好也都是相互的，你们也好。

P(8)：护士也比较专业。打针啊什么的都挺好的，经常过来的喔，吃饭没呀，今天怎么样啊。

P(7):但是我感觉，查房的话姜主任她特别的仔细，因为她看了我以往的病历因为查不出——她意思是如果查不出感染源的话，你到时候就像这次成功的到时候也有可能是跟前面那个一样，她还是挺负责的。主要是我第一次来，上个礼拜来，入院的时候没看没碰到他查房过了，她是周二查的吧。

R：嗯周二在，礼拜四也在。吴医师也好的。

P(7)：但是那天本来因为其实那天血也出来好几天了，等于17号我就出来了白细胞偏高。但是他们也没说什么

P(8)：主要是这样子的因为你还没确定怀没怀孕所以他们都是不加药的很保守的，像我上次没确定宫内宫外他们也不加8C7的。

R：光看一个白细胞指标确实不一定用药，用药有用药的指

征的。

P(7)：用药是不一定要用药呢，至少她应该——应该就是说——

P(8)：多高？

P(7)：十四点多

R：那你自己有没有别的什么不舒服的？

P(7)：尿频啊，尿不尽啊

R：那可能还是稍微有点泌尿系感染的吧

P(7)：尿常规是好的，原来是感染过的，但我吃了很多消炎药才吃好。吃那一种，苹果什么什么的，新出来的220一盒，我吃了5盒才吃好。好的消炎药吃了我差的没效果。

R：还有补充的吗？关于心理这一块

P(8)：我俩是心态比较好。年龄大一点——（可能没有注意，无意间将患者打断）

R：你的心态比他的要好一点，要好好地自己开导，我觉得你们经常这样聊聊，挺好的。相互诉说，也分享一下经历。

P(7)：想的——再怎么心里压力还是有的。

R：像你们这样，我觉得有这样一个群体，其实也在这方面至少有那么一群同病相怜的人也会格外的珍惜，应该现在这种朋友应该也会蛮多的，

P(8):这种要交那种正能量的。

R：对，那倒是就正能量对自己影响也会大一点，往好的方面影响。好吧，那你们先睡觉，挺晚了，快九点了！
