# Supplementary material for: An insurmountable obstacle: Experiences of Chinese women undergoing in vitro fertilization
Source: PLoS One. 2024 Oct 7;19(10):e0311660. doi: 10.1371/journal.pone.0311660 (PMC11458033; doi:10.1371/journal.pone.0311660)
Supplement: S1 Data — (ZIP) [file pone.0311660.s001.zip › data/P1.docx]

R：首先非常高兴你能来参加我的课题，非常感谢你能告诉我你的经历。我能先问一下，就是说你为什么会选择移植，包括您当时选择移植以后，比如说你移植成功，心里的感受这一类，就从移植这一块，你一路过来的感受，能先跟我说一下吗？

P：成功？一开始觉得成功了就...

R：你可以先跟我说说为什么会选择移植？

P：因为我之前做过卵巢囊肿手术，然后一边是卵巢囊肿，然后一边是宫外孕，所以一边是囊肿不好，一边是输卵管不好，所以一下子怀孕不了。然后就准备去做试管。然后这试管一开始觉得很轻松，因为觉得每个人都想着好像做试管把就是把卵取出来，精子取出来体外受精，然后就可以放进去了，显得都是好像很简单的这几个步骤。但是走进去 了才知道没有这么简单，从第一次检查到第一次取卵都相差4个月，一次又一次跑医院什么的，然后等到第一次取卵以后又没有配好，所以又要取一次。一共取了两次，最后才配了三个胚胎，太繁琐了。然后又要考虑要不要去上班，因为上班了就没那么方便了。

R：那是很繁琐

P：是啊，在医院里的话，每个人第一次去的人都会说，啊~知道来了才知道啊怎么这么繁琐的，然后又会考虑要不要上班啊，因为这个原因很大的费用又很多，对吧？然后每个人都会考虑费用的这块事情。像很多有些都是一边上班，一边在那跑医院，请假啊各方面都不方便呀。取卵的话，一步一步都是从不知道到知道，一开始做的别人会说怎么怎么样，都不懂。但是取卵，又会担心取几个泡泡，取的时候又会担心取出来是不是空泡啊，卵泡质量好不好啊，然后取了之后，又担心三天之后看胚胎情况嘛，所以又会担心后面的情况。我是第一次一共取了3颗，但是后来知道只配了一个，医生说太少了要再取，所以后来又取了一次。

R：为什么呢？

P：因为只有一颗不给我们移，除非我们自己去强调，我要先移，然后他们的话是建议，因为我们年龄也比较大了，然后卵巢功能也是越来越不好，然后建议他会建议你胚胎多存几个，然后再移植，这样成功率会高一点，所以又安排第二次取卵。然后第一次用一代的，第二次用二代就是是结合率会高一点嘛，然后就是取了三颗。取了三颗，配了两个，然后开始移植。

R：就你的感受是怎么样的？就心里面的感受。

P：觉得也还行，就是太繁琐了。

R：什么叫还行？

P：心情的话就这样一步一步走来，也还行吧，因为你说我比别人取出来一个泡都没有比那种好一点，最起码我每次卵泡都是三个取出来都是三个，我没有空泡，也没有什么就是配，配的话，就是两个人的事情，所以我觉得两次也差不多吧，比别人那种取了几个都是空泡啊，或者没有配好，比那种稍微好一点。所以也还行。

R：而且你是一次就成功了？

P：对，这个的话我一开始我也是这么觉得，因为我子宫环境是好的，我没有流产过。没有怎么过，然后内膜都还好。然后，移植之后成功了，当时心情，对心情就是挺好的，但是没有想到，比如说没有想到后面的事情这么难，就感觉成功了，好了就好像好了，因为他们邵逸夫的话也是着床了，就是成功了，后续的事情也好像管不了那么多的。

R：医院管的比较少。

P：他们医院里就像今天16床，她也是移植的嘛，她今天跟我说，他是35天就停药了嘛，因为邵逸夫那边的话，我们35天回院的话就各项指标都好的话黄体酮就给你停掉了，是就针不打了，然后她是停掉...也就十来天吧就出血了。然后我也是停掉一个星期。

R：药停掉了？

P：对，黄体酮药停掉了，

R：停掉是那边叫你停掉的？

P：对，所以那边就是各项指标好的话，他就把药停掉了。停掉之后就不好。他们，后来听他们说停药停得太快了。

R：你停掉以后，你是来我们这里住院的？

P：没有。

R：没有是吧？是直接不好过来的是吧？

P：上次我没有来。我不死心嘛，所以手术我就回我们那边去做的。然后，我..我也没想到会胎停，就一开始就..双胎嘛，双胎的话就有一个孕囊比较小就是，然后我一直觉得去做B超啊，医生都会说你这个可能到时候羊水不够啊，他说可能有点危险，他说怎么样。所以我一直觉得会觉得小的不好。然后。。然后没想到两个都不好。反正...

R：当时知道不是很好的时候，心里是怎么样一种感觉？心里的感受是怎么样的？

P：怎么说啊，心里当时就觉得不可能的事情。因为我从来没有知道说是做试管也会胎停，因为只听说过自然怀孕会胎停，但是做试管怎么也会胎停，因为各项好像都筛查过的东西怎么也会胎停呢。当时做B超的时候，医生说，没见到胎心了。我就说,”一个吗？”然后他说两个都没有。然后他就叫我取了白带，他说去做个彩超，然后然后去楼上做了个彩超，然后我就问医生,那个做彩超的医生，我说，我说那是不是要流产流掉啦？他说哎呀你还是去问医生吧，他说太可惜了，他说。那既然这样子了，我也还好，也还挺淡定的。就觉得想不通怎么会发生这样的事情。然后跟我老公说，我老公后来他跟我说，唉眼泪都要出来了，他说，你一出来就跟我说没胎心了他说。然后，我是还好，然后我老公就问，就问流产的事情嘛，就..我是还好。就是后来一起做试管的一起的这几个人，他们就，有一个也是在你们市中保胎的，她问我，我不敢跟她说，后来她说怎么啦？“哎呀”，我说，“我不想影响你们的心情”，因为我们都是那几天移植的嘛，然后他说怎么会这样在的啊，她说，“好吧，我不会受你的影响的，我现在心态很好了“。就，当时他们问我的时候就，感觉很伤心。要不然的话，也还好，就感觉，就是...

R：怎么伤心？

P：就很想不通，怎么会这样子的呢，就在想觉得怎么会这样的呢！我无法理解，然后心里一直在想，我孕吐也比较反应比较厉害嘛，然后我觉得他们说12周了就会缓一下了嘛，然后我说，再熬一下，再熬个三四周就好了。但是突然那天说胎停了，怎么会突然间变成这样？

R：也没有什么症状的？

P：就流咖过，流咖，然后说，有积液嘛，我邵逸夫那边我也问了，然后医生也说你开点中药吃吃，因为血指啊各方面都好的，他说B超就有点积液，你开点中药吃吃就好了。所以我还去杭州邵逸夫的时候之前那天我还又去开了中药，所以我觉得，脑子里面就是一个问号，难道我们那边的医院也不知道吗？我还是刚刚那天去医院的那天才胎停的吗？因为之前他们说胎停的话也会孕反啊，各方面都会不一样的嘛，但是我之前还是好好的，我来杭州的那一天，我还准备，我说要吐嘛，吐很严重的嘛，然后我还准备了塑料袋，但是奇怪那天没吐。然后就，我们做B超是下午嘛，然后上午在那里，就中午的时候在那里等，肚子有点痛，然后吐的话也还好，难受是有一点难受，但是没有之前那么明显。但是不说胎停的话，我们也不会想到，说是有问题，对，所以当时医生说胎停了，我就感觉，呃，感觉就很不好意思一样的。

R：不好意思？

P：对，因为我们那天做B超的话是这么长一排，就是那天去做B超的人，全部都是35天啊50天返院的那些人嘛，然后都是排队排在那里的，然后就觉得，后面的人都听到的嘛，就感觉，比如说我们听到别人不好，我们也会说哎呀她不好啦怎么样啊，那就感觉哎呀很不好意思，感觉我怎么会这样子的呢，然后之前都在那里等的时候，认识的人啊都有的，然后她是35天的时候说胚胎不好弄掉了，然后他们都问我你多少天了，我说今天49天，她说哎呀，恭喜你啦，今天过完就毕业啦，就这样。觉得挺好的。但是结果去做B超了说不好。所以感觉，怎么说呢？好像说不，说不出来，还碰到后面那个人，我们同一天移植的嘛，她说你今天来了，你怎么样啦？因为大家认识了都会这样子问的嘛，然后我说哎呀没胎心了，就，我就走了嘛，就感觉，呃，心情的话就感觉，唉怎么会这样呢，这么多人弄在那里,我怎么会不好呢？就，好像，呵，很没面子一样的（笑）。因为这么多人都听到啊，背后肯定会说的嘛，像我们也一样的，唉她怎么不好啦怎么样啊？所以，当时心情就觉得，唉很没面子一样的。凭什么我…我…我双胎我胎停啦，就这样的想法。然后就去做B超，然后，确定了没胎心啦。那又怎么办只能流产，然后又去问医生，医生说，那就胚胎染色体不好嘛，其它又没有什么好检查的。所以他就说，要么你现在就约流产掉，要么就你自己回去你们那边做。你自己选择，反正胚胎死在里面了，时间待长了，对你也不好。我们想想，也很犹豫，又不死心。然后，我说那回来吧，到我们这边再做B超，会不会好一点？但是，第二天我弟订婚嘛，桌子上面一桌的那个菜我都会吃了，以前的话看到那种菜，闻到那个味就吃不下的嘛，也奇怪，第二天就没有孕反了，所以，然后下午去医院，然后医生也就说，他说既然这样子，也不用，不用再做B超了。所以我同学就帮我安排住院。

R：当天就做掉了？

P：没有，那是。住了一个星期，然后一开始就吃药。我觉得，叫我吃药的时候就觉得我还在想要不要吃这个药，还不死心，但是觉得这些反应呢真的是没有了，应该死心了，但是那颗药吃下去，不是每天要吃几颗药的的啊，那颗药吃下去的时候，我觉得，唉会不会还好的呀，就还心里还在这样子想的。但是想想，这些反应呢一点都没有了，应该是确定是没有了。然后医生也说他帮我摸了一下嘛，他说子宫是有点下来了，他说太可惜了，他说，宝宝还有点偏大的，他说大的那个。他说那就尽早做掉吧。然后就觉得，有时候就觉得总是不死心。吃最后那一颗药的时候还是不死心，就这样没有了。所以，唉，从那一次之后，我觉得去做试管，我去医院也好，听到他们官方的时候，他们说啊，血指多少啊，我就觉得，没什么好高兴的。真的，因为后面的事情怎么样发生，都，我们预料不到的，谁都预料不到的，所以我觉得，你说他们都，哈我90多，我着床嘞，怎么怎么样，他们都很兴奋，我觉得，我觉得没这个必要。没什么好兴奋的。就顺其自然好了，像~你强求也强求不来。像他们说的你宝宝发育好么怎么样都会好，不好么怎么样都不好。

R：后来药吃了以后就做掉了是吗？

P：呃——先药流。

R：没流干净吗？

P：我也不知道，流的话，那个，不是流下来给那个医生看的嘛，然后医生说胚胎，那个胎盘，是叫胎盘吧，他说是胎盘都下来了。那胎盘都下来了是不是已经干净了？

R：那你后来有做手术吗？

P：有。手术我是做了，但是那个药吃下去的时候肚子是有点阵痛，但是那个掉下来了之后肚子就不怎么痛啦，然后做完手术的话，一点都不痛。做无痛的我也不知道有没有刮过，我也不知道应该刮应该也刮过。

R：那你能跟我说说刚做完手术那一会的感受吗？

P：刚做完手术，我做无痛的嘛，然后麻药一过...然后...

R：主要是心理这一块。

P：就...推出手术室，然后我睁开眼的时候就看到我表妹跟我老公在手术室外面嘛，然后就控制不住，看到他们两个人就哭了，哭了然后我表妹她就说哭什么啦，她说，会有的呀，她说没都没了，你还哭什么。然后就哭了那几下，然后就控制住了也就没怎么哭了。就那几下，我也不知道怎么。就...眼泪止不住的就哭，好像发泄一样。

R：可能是个脆弱点一样的是吧？

P：是的。后面的话也还行，因为我有什么事我也不喜欢跟别人去说。怎么怎么样啊，反正跟我家里人啊，我都是这样子的。也不去跟他们怎么怎么说，不好啊怎么样，我也不会到家里人前面去哭啊怎么样。

R：还是蛮坚强的，也蛮独立。

P：就像做试管的，很多人问我，有没有哭啊，我说我没有，我当时没有。但是回来了之后就一个人的时候想想就，眼泪会流下来（有点哽咽）。但是我说在医院的话，我是...我不去哭。然后他们很多人他说那...有一个人她说，她那次生化了，“我都哭了，我生化了都哭，你胎停了，你还不哭”。当时就是一个问号，为什么会这样子？然后医生也没给我们答案，就一个问号，一个疑问，然后心情的话就一个人的时候会稍微好一点。在别人面前我还是要装坚强一点。然后，我婆婆嘛一直在说，又要去相信迷信，是不是移坟啊移不好啊怎么样。

R：那你们家里人在这一块，包括你之前没有怀上，再到移植，然后再到移植以后受孕，最后再流产。就在这一个过程当中，我想知道你家里人的看法都有哪些变化，就对你有没有哪些影响？

P：我觉得家里人还好吧。我妈妈就是说我说小孩子不好嘛，就觉得哎怎么这么烦啊，就这样子嘛，老人家嘛，肯定想早一点呢。然后你说一开始好嘛，他们都也都高兴，对吧，最终还是了了一件事情。那后面又说不好嘛，我妈说哎怎么这么烦的啦，就这样子。其他也没怎么说。然后婆婆他们也还好，也没怎么说。我婆婆反正她就会说，哎呀是不是我老公的问题啊就是这样子，因为我老公比较胖嘛，她说肯定是他的问题，她说，这样子，然后她，因为现在村里也很多是这种胎停的嘛，然后她就说哎别人那种自然怀孕的也有胎停的，他说现在胎停的这么多她说。就慢慢的好像适应了嘛很正常一样的，就是。然后一开始他会说你们吃的东西啊有没有注意啊，就是说吃的东西有没有引起胎停啊这样子，那我说吃的话也是天天在家里吃的，也没怎么到外面去吃。所以家里人的话也还好。我老公的话也还行。

R：什么是还行？

P：也不会来说我怎么怎么样，他就说哎呀没了就没了呀他说，下次再重新来过嘛，也没有怎么说。就是，可能我不理解他，可能他比我还伤心也不一定。

R：你前面说你老公也眼泪都流出来了...

P：他说，我跟他说，我出来就跟他说，我说没胎心啦，然后他就说啊眼泪都流出来了，他说你一出来就跟我说没胎心来，他说，而且是两个都没胎心啦，他说呃...我真的他说眼泪都快流出来（有点哽咽）。可能当时还是他心疼一点。

R：心疼两个人肯定都心疼的。

P：其他的话他...他也他也不会在我面前表现的很那个啊，如果表现出来不好的话它也会影响我的啊。然后他也一直会说哎呀做试管啊，精神压力很大嘛，他就说哎呀花了这么多的心思他说还是不好。你说经济上面还不是个问题，最主要是花了心思，三天两头跑医院，还要担心小孩子好不好？像一开始的话觉得着床了，一步一步走来都好的，觉得很好。但是突然一下子这样子了，到后面就后期有点怕了。像他那天说，他说别人有些人保胎保到生啊他说。（过了一会儿）所以，可能他比我还心里压力大，看上去好像没事可能他比我还要大。

R：你们有没有在沟通过这些方面的问题？

P：我们很少提起。（停了一会儿）他就...他就会想着他就后悔。他说一开始的时候他说，早知道就到这里来看一下，她说最起码有一个能保住他说，但是如果真的是宝宝不好的话，那保了也没用。虽然，所以说这一次的话就早一点来。那宝宝不好，我们尽力了就好了，省得像上次一样说，哎呀后悔。

R：现在你回过头来想之前的这些情况，是怎么样的一种感觉？

P：哎呀经历过了才知道。对吧？不去经历谁都不知道。但是回过头来想一想，什么事都不要想的太简单。每一个人都是一样，做试管的，第一次是什么都不知道，到一次失败了，两次失败了，她就会更加的注重保胎这一块。上次我认识的那个在你们这里保胎，她移过鲜胚，好像一次还是两次都没有成功。所以他那天九天了她就着床了，她就到你们这里来保胎。所以，你说我第一次的话，我也，因为我很自信的，我着床肯定会着床的，但是没有想过胚胎会不好，所以我也没有想过说要保胎啊怎么样。然后我们那边的话说，中医院也是说可以保胎这样子，所以我说省得跑杭州来也不方便，反正就一点流咖，没有想到会胎停这些。然后血脂又是好的，然后，因为我们毕竟做试管的比任何人会比较紧张一点，有一点什么啊都会手机上问医生的，然后医生说问题不大怎么样，然后我们也会放心一点嘛。所以那边有中药嘛就中药开点来吃吃，反正也觉得他们会保胎。但是没想到会胎停啊这样子，回想起来嘛，有些事情也没得后悔的，对吧？

R：你还有没有，主要是关于你感受这一块，就对这件事情你还有没有哪些方面需要补充说的？

P：也没什么补充说的，哎呀，感受嘛就是，心痛肯定是心痛的。但是为什么我有时候在想为什么，医疗，做试管为什么不全方面完善一点呢？应该对于我们大龄的大龄的年龄来说的话，最起码你要一个稍微再完善一点。跟年轻人没得比的，各方面因素也一样。但是做试管人真的，太痛苦了。

R：你比如说怎么样完善一点？

P：比如说像我们胎停的这种几率啊，你们都要，比如说医生，你现在有第三代可以，染色体都可以查。那比如说像我们年纪大一点的，你可不可以就建议我们做三代？费用的话，你反正去做了一代二代三代，还不如直接做三代。对吧？比如说年纪轻的人，可以建议给他们做一代二代，毕竟他们体质啊各方面比我们年纪大的人要好嘛。所以我觉得年纪大了，比如说到几岁以上的，最好是直接建议我们做三代。这样子的话不是少走很多路嘛，要不然像，像我们这种年龄大的胎停率不是最高嘛？所以我觉得我只能在想我说，哎我老公都说了这次不好的话，等个两年去国外做他说，因为大家都听起来觉得国外好一点，对，成功率都好一点。

R：他们是一开始就给你做三代的吗？

P：对，应该是这样子的。所以他们一直说在国外的话，你想生女儿生儿子啊，他们都会给你做的。所以在我们这边的话，我觉得不走这一条路，是不知道这条路这么辛苦。每一个人都是一样的。包括我去上海看那个免疫啊，自然怀孕的人很少，都是做试管的人在那里看，都是一次不成功啊，说卵泡不好啊，都在那里调都在那里弄。反正去的都是做试管的人，自然怀孕的人说没有一个在那里看的，反正坐起来聊都是做试管的。

R：所以你们试管打的针也很多。

P：人真的是多。现在邵逸夫的话生殖科人是最多的，我在想想，这些人不孕不育普通的，200来个号最起码。早上都一百四五十。然后还有专家，然后还有妇二，取卵的这些人，二三十个，还有妇三。然后还有这些官方的呀。一开始我觉得我朋友她也是做试管的一次就行了。觉得挺好的呀，没有像她说的那么繁琐，没有像她说的这么烦。

R：比较少这种。

P：只听到他们说成功与不成功，但是没有听过胎停。但是我之前那个，我们也加微信了，她是官方了之后就说胚胎不好，然后就让她弄掉了也是，很早就弄掉了。想想，就自己安慰自己，哎呀比我还大的人也胎停的人也有，不好的人也有，对吧？就自己这样安慰自己，比她们好一点。（笑了下）

R：心态要好。

P：没办法，不想通也要想通，但是，害怕也害怕。（笑了下）

R：好的，非常感谢。
