# Supplementary material for: An insurmountable obstacle: Experiences of Chinese women undergoing in vitro fertilization
Source: PLoS One. 2024 Oct 7;19(10):e0311660. doi: 10.1371/journal.pone.0311660 (PMC11458033; doi:10.1371/journal.pone.0311660)
Supplement: S1 Data — (ZIP) [file pone.0311660.s001.zip › data/P2.docx]

R:怎么会想到去移植的?

P:其实我做试管也是当时机缘巧合，因为我之前是输卵管也做过手术嘛，那么我觉得做试管的可能相对来说，然后再加上我本身多囊容易引起宫外孕，我很怕这一点。然后我就抱着试一试的心态去找了一个我认识的医生，然后他就是在妇保的生殖内分泌科的，他说你过来吧，然后做了检查以后，就医生说你符合条件可以做试管了，那我就毫不犹豫我就签字了，我就做试管，因为我当时说实话，我就是奔着试管去的。我其实这个包括很多人不能理解我，包括我家里人，就是我身边的哥哥姐姐，他们特别不能理解，就觉得为什么要去做吃这么多苦，又不是没有孩子，在有孩子的前提下，你还要再去吃着这个苦，为什么呢？第一个确实从我自己内心来说，我觉得跟我爸妈有很大关系。他们非常希望我有二胎，那我老公是长期跟我们一起生活的跟我爸妈，跟他爸爸妈妈基本上是不太生活，因为他部队嘛，在杭州驻地在杭州。那么我们有时候就基本上都跟我爸妈住在一起，那我爸妈非常非常希望我生二胎。然后我觉得看到我舅舅生病的时候，我姐姐一个人东奔西跑，毕竟是女孩子，我就想到我如果以后生病了，然后我老公以后生病了，然后我不能拿主意的时候，我要靠女儿的时候，我觉得我女儿也需要一个支柱，也需要一个别人，有一些东西是老公不能替你做决定的。因为毕竟是你的父母，但是你的兄弟姐妹可以为你做决定，可以陪着你一起做决定，这样你的压力会分担的小一点。我站在我的角度是这样子，我觉得我有一个哥哥，其实我上次也跟你说过，我哥还是非常疼我的，其实一直以来对我也都挺好的，所以我觉得有一个兄弟姐妹在身边挺好的。

R：除了这方面的，我更多知道的，比如说你移植之前对试管了不了解。。。

P：完全不了解，对，其实很多人觉得做试管很苦很痛，因为我也不了解，我也抱着第一次去尝试的心态，包括我去打促排以后进入周期，移植之前进入周期，我从来没有问过医生我的治疗方案，我就是蒙着去蒙着回的，就他说你到什么阶段该干嘛，该干嘛就干嘛，他跟我说什么我就“哦好可以”！就这样子。其实之前我没有多少了解过试管，我只知道，我甚至分不清楚试管和人授。医生跟我说，他问我，他说你做试管还是做人授，因为要签字嘛不是要建档。我一脸懵的看着她，我说我不知道，然后我老公也说，我也不知道，然后我们两个就进去，然后他看我们的单子，他说你做试管，我说是试管，然后我后来我经过这个事情，我才去查了一下试管和人授的区别是什么，以前我就觉得试管就是人授吧，差不多。

R：那你自己这样子一系列经历下来，对于这个过程有什么感想呢？

P：我觉得没有大家说的这么恐怖吧，因为我身边跟我一起移植，包括十楼有个病友，他跟我同一天移植我们在一起移植的。就觉得这个过程除了取卵的时候，就是打针嘛麻烦一点，因为经常跑医院要抽血要打针。其实你说多少痛苦真的好像还好吧。

R：觉得还好。。。

P：我个人觉得，然后我那天跟他聊，我也觉得我说我也。。。

R：你是第一次就成功了？

P：嗯，第一次我就怀孕了，但是后来不是胎停了吗，所以还是挺可惜的。

R：就除了可惜有没有别的？

P：那肯定很难受的。

R：能不能跟我说的具体一点？

P：因为其实我上一次移植我的心态本身也不是特别好，我觉得我还没调整好，包括我的身体，我觉得我也没有调整好。那个状态，因为三个月嘛就是我取卵以后的三个月进入移植的周期，我当时也没多想，然后别人都去配中药干嘛，然后我是到了移植那天，我才知道这么多人喝中药或者调理干嘛，完全没有。完全没有，然后我就去移植了，移植完了以后我就在家躺了大概躺了六天，第六天开始我测试纸已经有淡淡的痕迹出来，那时候很开心的，但是每天测，然后颜色也在每天加深，然后我一直以为我的指标会非常好，那时候抱的期望是很大的，因为我们当时有进一个群，然后后来我才觉得这个群真的不能进不能加，因为大家都会去套感觉，然后都会都会胡思乱想，你知道吗？就想的越多，你期望就越大。然后后来就就去抽血，抽血指标出来很差。很低，然后因为我是第12天去抽的，HCG142，第12天142，然后我一开始我还觉得反正怀上了，然后有一个比我晚查出来怀孕的人，他都超过我了。我当时心里就很失落，中午都吃不下饭，那个心理，真的就是然后别人跟我说什么话我都听不进去，然后我很想发脾气。然后我姐姐那个时候她很关心我，就是我这个大姐姐他真的很关心我，把我当女儿一样看待，她说没事你就早点回去，我当时就对着他发脾气，我说你们不要给我这么大压力。当时心情那样就是那个点。然后后来第二次测HCG的时候，我已经住在医院里了，测出来我当时在红会保胎。是300多，我觉得翻倍，反正。差不多吧，300多也翻上去了。我觉得应该还好，然后第三次抽血的时候，第二次我还没住，第二次抽完血我住到红会去的。然后在红会第一次抽血直接翻到了一千多，那个时候，超级开心。心里很激动的，然后从那天晚上开始我就一直失眠，我也不知道为什么，就是一直失眠，然后到下一次抽血的时候，我就心里感觉非常不好，就好像女人的第六感一样，就感觉非常差，因为我已经连续两晚上失眠了。然后抽血那天我就特别着急的看，翻结果翻结果不停的在翻结果，然后出来，HCG没有翻倍。然后我就去护士那里，然后因为我姐姐是红会的护士长嘛，有一个，然后他就联系了科室的护士长，然后就说重新做方案或者干嘛，他也说了，说你反正我们尽人事，然后就打了一次HCG针，然后加了药，但是还是不行。然后后来就哭啊那没办法，然后哭，哭完，出院呗就觉得待在医院也没有用了，然后过了两天去了妇保挂了号，然后说一开始以为它自己会生化掉，然后医生跟我说不行，然后我再我再做清宫，然后这个过程也很那个，他让我去计划生育科，他说你先把药都停了，然后去计划生育科挂号，然后准备住院。然后我其实住院以前我已经有绒毛组织掉下来了，我自己也不知道，觉得肚子很痛，然后我就觉得肚子很痛，有一段因为我那天刚好去扬州了，就回我老公老家带我女儿过去看他爷爷奶奶了，然后肚子很疼很疼那天，然后我第二天去医院就是住院的，然后医生就跟我说，好像有东西已经掉出来了，就还是有那个宫口，因为流血比较多，他说有东西夹出来，然后她就帮我取出来，然后就第三天马上就安排我做手术。然后他们那个手术是不打麻药的。他们人流不打麻药的。然后其实我进去很快，因为好像，掉的就一点点了，然后让我去送检，然后那个邵主任说这个太少了，不一定能检的出来，对，查染色体。查出来胚胎是好的。还是我自身的问题，所以还是我前期没有调理。然后我病友，所以我每次运气还挺好的，遇到了个病友，他就跟我说，他们跟我说何嘉玲医生很好，然后我就去找，挂不到他的号啊，没办法找人啊，找人找熟人，他以前看过的一个病人，一个病人跟她关系特别好，大概是十几年前看的病人跟他关系特别好，刚好是我们领导他帮我去加的号，然后我直接就挂进了，所以我说，然后我们领导还说他说佳佳我早就跟你说过了，让你去看中医，看中医，没有人，你每次都不听我的，我说我现在想看中医挂不到号。他说你想挂谁？我说何嘉玲，他说我一直叫你去看何嘉玲啊，她说你都不去，然后我也觉得是蛮巧的。

R：你刚才跟我说你第一后面测有一次，HCG前面感觉也没有太大的感觉，我看你就是到后面有一次测出，HCG没有翻倍，你说突然就失落了。

P：那肯定的，是很慌了，当时心就很慌了，因为你我在住院的时候，我老公在外训，就没有人陪在我身边的，就我一个人，我不知道跟谁说，然后家里人不停的在问我今天情况怎么样啊，数字好不好啊？其实我真的不知道该怎么说。你说，你说现在家庭谁都不缺吃不缺穿，但是你说做一次试管一次金额也是要小几万块钱，那钱也不是天上掉下来的，对吧？而且因为我妈知道我要做试管，他就觉得我已经很吃苦了，所以这些钱都是他出的，不管他现在不出得起这个钱，但是我觉得但是我心里那个点就很难受。

R：你觉得哪些原因引起的那个点难受？比如说压力大，哪些原因引起压力大？

P：肯定压力大的呀，因为你要想着你这一次不成功，你势必还要再来一次，对不对？而且所有人都跟我说一颗胚胎很难成。如果不成，因为我这个人就想的比较多，如果不成，我是不是还有勇气再走这么一遍，就要考虑很多东西的。然后我吃了这么多苦，别人都成了我为什么不成，我为什么不能成？从小我就很要强的，个性上，我特别要强，我就觉得别人能做到的我肯定能做到。所以包括那个时候我去当兵啊什么，我们班长都说，他都说，因为他们都知道我家条件还挺好的，他说你一点都不娇气，我就是那个时候打背包嘛，手全破了，因为冬天我们打背包手全破了都流血了，我自己一点都不知道还在那打打打。然后我班长说你手怎么了？我说哦可能太干了，那个时候就还是继续打。然后我们班长当时就说，别看，而且我当时是最小的一个，有些比我大四岁的也有，然后我们班长就说，别看你小，你还挺能吃苦。因为我觉得有些东西我觉得别人能做到的，我肯定能做到，别人做不到的，我也想努力去把它做到，所以有时候我对我女儿的要求也很高，我有时候觉得她也比较累。

R：可能自己给自己压力

P：对，我是很会自己给自己找压力，我如果明天开会要汇报材料，我今天晚上如果没写好，我做梦都在开会汇报材料，写材料，我就是心理压力特别重的人，所以有一段时间我也抑郁过。所以我就觉得然后那段时间太累了，因为G20那段时间，真的我觉得我这个工作我都感觉我。。第一个做好像。。因为G20那段时间工作就是重复反复，然后我就觉得我做这个工作有什么意义，我学不到任何东西，我得不到任何提升。我不是说职位上面提升，我说我个人，我好像。。就是得不到任何提升，我没有，我不能学到新的任何东西，我感觉我永远在这一个lever上我永远上不去了，我就感觉好像人生就有点怀疑自己了，我就特别难受那段时间，然后我老公我妈我爸一直陪在我身边，慢慢就好起来了。

R：那你后来就是手术后那时候觉得是怎么样，自己的心里是怎么样？

P：就我偷偷哭，那能怎么办？

R：我们今天的聊天还是主要以你说的状态为主，偷偷的哭，讲得具体一点，就比如说你哭的时候，你想到的是哪些方面？哪些点让你哭了，想到哪些方面你特别伤心？

P：作为一个妈妈来说，我觉得孩子没了难受肯定的。不管就是这个小孩在你肚子里多久，或者是感情有多淡，但是你吃了这份苦，你就会觉得一个孩子没有了。然后我可能。。唉比较幸福，我就觉得好像自己作孽一样，我觉得特别难受。因为我觉得我就觉得别人都可以，为什么就我不行呢，为什么就轮到我就变这样子了？是我哪里不对吗？我就会这样去反思。我觉得到底问题出在哪里，我就很奇怪。心里真的很难受，就想这些东西对。然后想也会想。

R：能具体点吗

P：让我再具体一点，反正我就觉得，然后那段时间我觉得我也挺对不起我爸妈的，虽然说他们可能不那么想，但是我自己的心里，（停顿）就是对我父母，我还觉得挺。。挺亏欠的，然后我哥哥姐姐都跑来跟我说，不要再做啦，不要再做啦。。其实，我当时我就发了条朋友圈，我印象还挺深刻。呃。。我就说，其实，走到今天，我自己也没想到我能坚持到今天，但是我觉得既然到了这一步，我就觉得，呃，我觉得还是，我希望更多的人能够支持我，不要去否定我。因为，我还有一次机会，我不想放弃，我就觉得家里人给我多一点鼓励，陪着我就好了，其他的就不要再说了。我的希望是这样子。然后包括我住院进来的第三天，我哥又给我打电话，他就说，呃怎么样，然后我就跟他说还好，然后她第一句话就。。“我跟你说不好就不要再。。。”其实我更多的是希望你们能站在我的角度来多鼓励我。因为我觉得我走到这步也不容易，我不想总是听到否定的声音，我知道你心疼我，但是，我觉得有时候还是就是家人，还是按照当事人的想法走比较重要一点。我觉得不能，因为，因为一些事情，比如说因为你觉得这样对我不好，用你的想法来加在我身上，但是，我希望是我能继续下去。我是这么想的。（哽咽）其实最难受的还是我觉得，然后我姐姐也过来跟我说，因为试管当时是我妈妈希望我做的，然后我姐就过来说，说如果是我女儿，我绝对不会让他做，做这么伤身体的事情，因为作为试管来说确实是非常伤身体。然后我就特别希望我不希望这件事因为这件事情去怪谁谁怪谁，所有的选择都是我自己做出来的，我长大了我应该去承担我自己做的选择，我应该去承受，不管结果是好和坏，但是这个都是我的选择，我希望更多的人能支持我，而不是一味的在否定我否定我，因为我做出了这么多努力，我付出了这么多努力，我吃了这么多苦，我知道你们也希望我成功，你们也怕我吃苦，但是既然到了这一步，我就希望你们能更多的站在我身边，支持我，鼓励我，而不是在你们觉得这样是为我好的前提下，然后就去这样说。然后有时候看着就是，因为我们有时候会加微信，看着我们就是一起移植的那个他们朋友，然后肚子一天天大起来，那种心态也很复杂，就是既羡慕又难过。因为你会想到说，如果当时小孩还好的话，也差不多，我预产期当时是4月17号嘛，差不多快生了，我也知道这个东西没有这么容易，但是我觉得为什么这么多人都能成，但是我不成。然后我本身就是一个比较喜欢钻牛角尖，又比较要强的一个人，所以我有时候我自己也知道我很容易走进死胡同。所以我都尽量，尽量的去调节一下（心情）。

R:那你调节心情通常会用哪种方式呢

P：有时候会跟我朋友出去聊聊天，然后呃有时候我特别特别累的时候，我就会自己，因为我也不想让我的父母也不想让我的孩子，或者是，因为我老公也常长期不待在我身边，我就会自己找个地方，然后大哭一场释放掉，我觉得就很好。然后回去以后就什么事情都没有，但是你整个心态都不一样了，你还是可以继续很努力的工作，可以调整好家庭和工作之间的关系，我觉得女性还是要，就是还是会有一点不像男性这么，这么大条神经，还是比较细腻的，所以有时候还是要找到适合自己的一个方式去调节。（停顿）

R：其实我特别理解，因为我也想要个二胎。（此处逃了会近乎）

P：我很多同学，他们那个时候妈妈生了一个女儿，然后就拼命的追儿子，拼命的追儿子，那话一分为二讲，既然想要了二胎嘛，你是第一个生了女儿。那第二个多多少，我也希望我第二胎是个儿子，毕竟我已经有个女儿了吗，凑成一个好字那是最好，对不对？那实在没有，我觉得女儿也是比两个儿子好。

R：对我也是这么想的，我觉得我的想法也是这样。

P：因为第一个生女儿，第二个我觉得就放心大胆的生。那至于你说这个东西，我只能说我尽力怀，对不对？那我不可能去决定他是男是女。所以有些人他说我就喜欢女儿，其实说实话人的内心都会，我总想一个。。我第一个生儿子的，谁不想第二个生女儿，对不对？那就像第一个生女儿的都想要生儿子。

R：你现在回想就先回过头来想当初的事情，是怎么样的感觉？

P：我现在不敢想。我就这个阶段我不太敢想，因为我怕我情绪会有变化，对我现在这个状态不是特别好。所以我，所以我尽量不去想之前，但是我这次移植以后，我的心态跟上一次完全不一样，上一次我整一个人处于神经紧绷到不行的状态，但这次我就很放松。

R：为什么？

P：我也不知道，可能我，呃，就觉得我自己上次的心态也没有调整好，所以这一次我尽量调节好心态。还有一点，我觉得这次——我也不知道为什么心态也比较好，感觉比较好。然后那个时候第六天，第六天我不是试纸没事出来嘛，就是白白的，然后我这次去了两次灵隐寺，就是移之前和移之后，然后第七天我就去了灵隐寺了，然后第六天下午我就沾了一卦，我就找那个占卜的师傅，因为我微信上有他嘛，我占了一卦，然后占完之后我又去了那一次，他说你要有寄托，你不要太绷紧神经，所以那时候我开始尽量的慢慢调节。所以我觉得这次移植的心态要比上次好多了。就是今天开始我感觉有点孕吐的反应，我也不知道不确定是不是。。

R：你现在移植第几天了？

P：第16天了，然后我刚问另外一个今天你们值班护士，然后我说是不是我胃难受导致的，然后他说给我看一下舌头。

我就给她看了一下舌头，她说，没有，就是孕吐反应，因为我刷牙干呕，我吃完饭也会干呕。

R：但实际上稍微早一点，有些人确实是稍微早一点。

P：但是我的数值（指HCG数值），因为我数值不高啊，所以我觉得我应该不是，因为我之前是吃阿司匹林有恶心的反应嘛，但是好像跟这个恶心有点不一样，对。那我当然也希望是孕吐。那不是嘛也没办法。对吧？

R：不是也正常。

P：对，反正就是药吃太多了，我胃也不舒服。反正这次我还挺平常心的，行就行，不行就不行。

R：第一次按理说像二胎的，但是我觉得我碰到的倒也是挺少，会这么的紧张，所以今天其实我也是挺意外的。

P：其实我第一次移植的时候，我们当时有三个二胎的一起移植的，但是我不知道他们后来成了没，因为我也对不清楚哪个是哪个了，后来在群里。然后第二次移植，就这次移植有另外一个二胎的，他第一个也是移植的。马上就成了，然后她小孩已经十岁了，七周岁了，上小学了嘛，然后他说他反正有胚胎冻着，他就说那我就移就移吧，那就移了一次。

R：那我补充问一点，我从前面人家反馈给我的信息，觉得移植他们觉得是非常繁琐，非常艰辛的。但是我看从你这边反应过来，好像也觉得也还好，关于移植的过程。

P：艰辛吗？我觉得是躺的难受，其他我都觉得还可以，都没有什么，这不就打打针嘛，你要想到你原来做这个事情的时候，这条路就是不好走的，所以我不喜欢。。。我就可真是我个性有关系，我不太喜欢，就是你明明要走这条路，你明明知道这条路走的很难，你既然选择了走这条路，你还要在这条路上唧唧歪歪唧唧歪歪。我觉得这个是我没有办法去理解的。我觉得你选择了你就坦然接受后面一切的困难，不管最后是结果是什么样的。

R：也就是说你当初选择移植的时候，其实你也是知道这么一个情况的。

P：那肯定是——就是，我不知道，其实我从来没了解过移植，但是我这样一路走过来，我觉得所有的苦我都还能承受啊，我觉得不就打个针吗，有什么可以承受不了的呢，那你生病不得不得挂水吗？不是一样吗？

R：但是要天天打呀。

P：那我第一个的时候我也天天打，因为我孕酮低嘛，我一直打到三个月，所以我。。。不是我就觉得打个针这个不算，我觉得这是大事吗，对啊我觉得这不是大事啊。其实我觉得在我看来这条路不管多难走，第一个我选了，第二个，如果结果是好的，我觉得所有吃的苦都是值得的，那如果说结果不好，我们换位，换句话来说，只能说是我缘分没到。那我不能说，我，对吧？我我能做的我都做了，该做的我也都做了。

R：这一点心态我觉得很不错。

P：那没办法，你就是，所以很多人那时候我去当兵的时候也是这样，很苦耶，但去了，你也就觉得不过如此，就是你可能心理上承受的压力，因为女人多的地方毕竟。。然后那个时候我们还要给班长洗衣服，你在家都不会给家长，给班长倒洗脚水，就你心态差一点就会崩塌，就觉得我在家我都没给我爸妈做过这类事情，我在这我凭什么要给你做。就这样，但是你想你来了，你就得适应，你让大环境适应你是不可能的，你只能自己去适应这个环境啊对不对。反正我一直一直以来都是这样想的。

R：挺好，我觉得。我也学习了。然后，胎停以后就是前面他们给我的信息，然后他们感到的最多的是意外，就会觉得，觉得为什么会胎停，感到很意外。

P：我的感觉我不是意外，就像我跟你说第六感很准，前两个晚上都没有睡好，所以住在这里第一个晚上没有睡好，我很担心，就是上一次我去套感觉就没睡好，我就特别难受，我就很担心。然后我觉得我更多的不是意外，我感觉到了，就是两天没睡好，然后我那天抽血，然后我就拼命去找化验单的时候就感觉到了，不太好。不是意外的感觉。

R：反而觉得不确定，没有把握。

P：就是你没有看到报告单的时候，那时候慌，就是心特别慌，但当你看到报告单以后你就觉得，果然没翻上去。就是，就是这种感觉，你的感觉还是准的，意外我倒觉得真的不是意外吧。只是觉得就像我刚刚说别人成，我没成。疑问，更多的是疑问。就觉得我哪里做的不对，我都跟他们一样的步骤，我都跟他们一样的，药，在用，对不对？而且我还生过孩子，所有人都跟我生过孩子很好做的啦，很容易就怀上的啦，对不对？都是这样说。

R：在社交上你觉得会影响吗？

P：还好吧，有什么影响？哪一类的影响？对身体吗？

R：不，社交方面，就是说朋友间啊，甚至是一同移植的这些人，一起交流，或平时你们之间的相处

P：我觉得这点我还是很坦然，没有任何的。然后他们当时来问我我就说，唉没就没了吧，也就没有缘分吧。

R：可能二胎跟一胎心态会不一样吧。

P：对，她们可能就是心态上面会更加的难受一点，因为他们做了试管之前可能也做过人授，这一点上他心里就更加那个，然后没有怀过孩子，就突然掉了一个孩子。你心态上会崩掉的。有一些真的会崩掉。尤其结婚好几年了，婆婆也比较催的比较着急。

R：那你会一个人偷偷的去想这个事情，偷偷的去伤心吗？

P：会的那肯定会的。我不会，我刚就说了我说我流产以后，可能有一个星期吧我就自己想着想着就会哭，然后家里人在给我这一类的意思就是说你不要再做啦，我就觉得，我觉得更多的是他们的不理解。我觉得他们是心疼我，这点我也知道，但是我觉得我已经到这一步了。然后再来说这样子的话，我觉得作为我来说，我肯定是以尊重你的意见为主（指家人应该尊重她），因为这件事情是我经历的，是我自己在经历在感受的。那么我决定我要走下去，你们，我希望你们更多给我的是支持和鼓励，而不是在打击我的信心，我知道你们是为我的身体考虑，但是我觉得呃家人之间不但，怎么说呢，各种相处的方式不一样吧，你们疼爱也是一种相处方式，但是我需要的是支持。包括我的很多领导都回来跟我说，因为我是单位里上班的，他们也都说不要做了，不要做了，其实我就笑笑，因为他不是我家人，他说我无所谓，我心里其实没什么感受的，反而是跟我走得比较近的人，他们就会说佳佳你一定能成的，肯定的。就说更多的是鼓励我。因为其实他们鼓不鼓励我反而觉得不是那么重要，但反而家里人的鼓励我觉得特别重要。

R：你可能把亲情看得特别重要。

P：我肯定，如果我不是把亲情看得这么重，我肯定不会生二胎。因为生二胎完全就是为了我爸妈。因为我女儿跟我姓的，然后我二胎生下来，我老公这点他也很好的，他说不要两个不一样，就两个一样，就信我。然后，所以他这么好，我坚定生二胎。然后我爸妈我爸退休了，我爸原来也是算是一个小领导吧，退下来以后他的心态也不是太好，然后我就一直想说，反正我也打算生二胎，那就让他有点事情做，分散一下那个注意力，所以我就一直特别，一七年开始我爸退休的。然后我就开始备孕二胎，一直到现在。

R：就是说备孕时间久了，你可能压力也会大一点。

P：那肯定会有的呀，你说你，尤其是第一次失败以后，我这次压力其实还挺大的。但是心态不像之前那么崩了，不像那个之前神经那么紧绷着，就会调节一点。还有一点就是这次我老公都在身边，那十天他都照顾的，那整个心态来说都还可以。

R：就是经历上次的事情以后，你会不会感到有没有一种害怕什么的这一类？

P：没有，就不是害怕，你知道吗？我觉得害怕这个词稍微深了一点，稍微重了一点，我觉得会担心。原来的那个重蹈覆辙这样。就突然间翻不上去了，所以我特别一直关注HCG的数据，对我特别关心，因为他们像我隔壁床，他说这个有什么好担心的，它更多担心的是雌二醇，因为他的雌二醇不好，但是我是上一次这样经历了以后，我就特别担心HCG的数值。不能说害怕，我觉得害怕，我觉得在我心里就觉得害怕有什么用呢。但是你多多少少，你说你完全不想着这个事情，是不可能的，肯定不可能，没有一个人说，如果他说了这句话，我觉得他完全是在骗人。对吧？但是我觉得会担心，会担心一些数据，因为从我每次去我第一次去，那个医生，妇保的给我移植的主任，他也跟我说，数值偏低，然后我这次去，他又是这么一句话。那我肯定很担心，我就想第一时间住到医院里来。嗯，所以我就，就在那天就住进来了，我觉得会担心，但是没有害怕那个程度。但是我觉得住到医院里来就特别想我女儿，真的就特别想我女儿。天天发视频，她来看我两次了，我来了一个星期嘛。其实我有时候，这次我就尽量让自己不去想这个事情，不过这次也还好，我没有怎么想，就抽血的那一天。可能会有点小担心这样子。其他的就还好吧，然后就手机看看啊，就当度假呗，忙了这么长时间，好几年都没怎么好好休息，就当时是给自己放个假呗！

R：我觉得你有时候给自己压力太大了。

P：对，所有身边的人都。因为我总想把一些事情做到好。我，我，那，做到100%满意是不可能的，但我至少希望我做的事情里面，有百分之六七十的人是觉得满意的，我就觉得挺好的。

R：还有一点关于——比如说你去移植嘛，我想以你这种性格身边的人什么肯定也都知道的，到后面小孩子没有成功，你又给自己那么大压力，你会不会觉得让你挺没面子什么的？

P：我觉得面子——好像在这个事情上不重要吧，我觉得这个重要吗？我觉得这个真的不重要。因为你很多人知道，他们也不会去说哎呦他失败了干嘛，我是光明正大的做，我跟我自己老公去想要一个孩子，我觉得这不是很正常的事情。所以我觉得这个不存在面子的，就是觉得挺可惜的。

R：比较坦荡。

P：我觉得我又不是跟别人生，我又不是偷偷摸摸的。所以我觉得，我跟我老公生啊这有什么啊，所以，但你不能说没面子，我就觉得挺可惜的。但是有一点，虽然我说我不介意别人知道这事，但我也不喜欢大家问我一些很细节的问题，尤其是在外面，因为毕竟这是个比较隐私的问题。然后有时候吧我不知道怎么去面对别人有时候，就是他们会，就是会关心，就你这个关心你知道，但是你不知道怎么去回应，因为没有人知道你受的苦，没有人知道你吃了多少苦，你经历了什么，这个只有你自己。所以有一些人的关心你——怎么说呢也不是说不好，但是我就觉得，如果是我作为我来说，今天换作你是我同事，你出了这个事情，然后大家都会觉得说，哎呦挺可惜的，下次就自己注意，但是如果是我的话，我就会觉得说，你来上班了就调整好心态，就是我也不会过多的去讨论这些事情，对，我觉得没必要啊，以为这个毕竟是别人的事，我觉得还是更多的让别人自己去——去吸收吧，去慢慢消化，我觉得这样比较好，因为提的多了，有时候女人就是那个点比较弱一点，你提的多了，他反而会觉得好尴尬，他不知道怎么去继续面对。

R：可能你也就存在着这样一种可能，觉得稍微有点感觉。

P：然后我还没我刚出院，我们同事就来家里看我，所以也没什么。就是说好好休息什么的。就我觉得这样就挺好的呀，就大家就跟聊天一样，不要去故意提这件事情。就好了呗。

R：你自己还有没有什么想补充的？

P：没有。差不多了吧，我就觉得没有——整个这条路走下来，没有想象中这么苦。我觉得他所有的一切都是在我的承受范围之内的。因为你就像我刚才说的，我没有了解过多少多少怎么怎么样。但是你当时选这条路，就知道他很难。你就要吃很多苦。

R：你没有了解过，你怎么知道它很难呢？

P：因为你，因为我身边有这样生不出孩子的人，我有个姐姐，我没有了解过整个过程，但是我知道他做了试管他也没成。我就知道这条路很难。然后很多人都说很痛什么的或者干嘛，我也会给自己，很痛那怎么办？很痛那只能承受，然后我取卵打了麻药，我就觉得醒来最多就吐了一下，就麻药反应嘛。所以不打麻药的话，我整个过程我都不知道我自己怎么坚持下来。现在换回来说。因为有打麻药，我就考虑到还是打麻药。我觉得整个最痛苦的过程不是打麻药，也不是取卵也不是移植也不是打针。

R：等HCG结果？

P：也不是，这个我觉得也还好。最痛苦的过程是灌肠。就是那个取卵之前喝的那一壶水，清肠道的，我觉得真的是太难受了。

R：那还好的呀，我以前做肠镜的时候也喝过，还好的呀。

P：但是我受不了，真的受不了，一千毫升的水，对。要在两个小时之内喝完，对！天哪那个水味道，我现在闻到那个味道，我还有点恶心，就是回想起那个我还觉得这个是我在整个路上面最痛苦，如果你真的打针都觉得受不了苦了，干嘛的。那你，那你当时就不要选择这条路，那你，其实我说不管你知不知道这条路是怎么走的，像我之前我也没有了解过，但是你肯定知道这条路是很难的，对，这肯定是很难的，那你在这干嘛呢？对啊，肯定打针吃药是避免不了的，你要想。所以我就觉得干嘛呢这样子。（讲了一段自己参兵时的经历）后来我说我一直以来都蛮幸运的，自己想要的都做到，唯独在这件事情上有一点磕磕绊绊，我觉得，唉，谁的人生没一个坎。

R：是的，也不要给自己太大压力了。而且不是也挺幸福美满的，现在也挺幸福。

P：对，反正我后来我爸妈也就看开了，其实我爸妈也很紧张。其实家里人的情绪你也会很关注的，你会觉得移植以后你家里人特别紧张，她不敢问你，好不好？舒不舒服？情况怎么样？他不太敢问你的，他又怕好像就是给你太大的压力。但是不问她很难受。所以整个紧绷的状态不是你一个人在承受，而是你整个家庭的氛围就是特别紧绷。反而我倒觉得我老公跟没事人一样，对他无所谓，他说不成就不成了，那能怎么办呢？对吧？该做的都做了啊。然后我爸妈就，但我妈心也蛮大的，我说你明天来给我送饭，她说明天？我好几天没去跳舞了，明天要去跳舞。

R：她会不会是故意让你放松一点点，

P：没有，然后我说随你随你，他就是这样子的，他特别爱玩。所以当时我生第一个的时候，女儿的时候我爸就怎么办，你妈这样，怎么办，好担心，后来也不就带过来了。

R：你爸爸还挺操心的。

P：我爸其实挺操心的，他比如说家里水开了，我说爸我去倒，他说不要，你们都不要动，等下烫到了。就我说我心想，多大了已经。

R：你看你在家里也是不用做事情的。

P：我们家一直都有阿姨。我家一直都有阿姨十多年了。反正觉得，唉就慢慢来呗那能怎么样，它成最好，不成那就考虑一下下一步自己到底是怎么样一个路去走（讲的比较慢）。关于这个二胎，但是我一直很坚定地。我就我觉得反正都已经到这一步了。即便不成功，我要首先，就是不生二胎，首先我要把我的身体调养好，就只能这样想，就只能这样想，所有人都跟我说，包括我同事他就说佳佳其实不用这么着急的，她有时候也不好讲太多，他就说不用这么着急，女人啊，你身体，身体变健康了以后，她说我们这几个你问问看，哪个没有在30岁怀过，都怀过。30多岁的时候最容易怀孕的，然后他们都是这么经验之谈，跟我说，因为我没有经历过，我也不知道啊对吧？

R：你看我今年都35，我还想再生一个呢！

P：还有一点，其实我生二胎的原因是我觉得家里人丁兴旺一点，整个家庭氛围都不太一样。那我哥跟我嫂子确实年级比较大了。我嫂子比我大12岁，43岁，我哥40。怎么说呢，我妈不能对我哥说太多，因为毕竟不是他生的，对吧？但是我爸也不能跟我嫂子说你们生二胎啊。因为我哥和我嫂子确实很难赚钱。

R：他们也就一个是吧？

P：对，他们真的是很难赚钱。所以我爸在这方面，然后觉得他们年纪也大了，不想要求他们太多。然后我嫂子之前也说过，她也不是很想生。那我觉得作为我来说，我第一个我年纪不是很大，第二个我觉得父母有这样的心愿。我觉得又不是我做不到的事情，我觉得这个事情明明是我可以做到的。既然他们想，我觉得我可以生，然后很多人都说你不要为了你父母生啊。

R：但是我觉得你底子也是想要两个的。

P：对，因为其实我挺想要两个孩子，我觉得孩子。那换句话来说，上了小学以后可能会很累，那累肯定是会很累的，但是你累。你累这么多年，后面呢，对啊。人生哪有一帆风顺的，对吧？其实想开就好。
