# Supplementary material for: An insurmountable obstacle: Experiences of Chinese women undergoing in vitro fertilization
Source: PLoS One. 2024 Oct 7;19(10):e0311660. doi: 10.1371/journal.pone.0311660 (PMC11458033; doi:10.1371/journal.pone.0311660)
Supplement: S1 Data — (ZIP) [file pone.0311660.s001.zip › data/P3.docx]

R：我们先来聊聊，你怎么会想到去做移植？

P：我是08年结婚的，然后结婚之后，一直有一年半的时间，我不是没有怀上吗，然后我就是做了一次输卵管造影，造影做出来是两侧都通的，然后造影做完之后，他就说好像就两个月之后可以自然怀孕吗，结果我竟然怀上了，怀上之后么结果就是89天的时候胎停了，胎停打掉嘛他说我有甲亢，甲亢之后么就是我治疗了两年甲亢，两年甲亢治疗完之后，医生说可以自己又可以怀了这样子，那我已经32岁了，然后我又花了三年时间，就是看医生各个地方，就是看医生吃中药，然后又是检查这样子，还是自己一直怀不上。然后后来到上海，最后到了上海红房子医院，红房子医院有一个叫徐军的医生，他看了我的输卵管造影的片子，然后他又给我查了什么AMH那个数值（抗缪勒激素），然后他就说了，他说好像卵巢功能有下，就是已经有下退的趋势，他说就是35岁之后你去做试管，他说成功率也会低的，我当时也不知道做试管有存在这种——我以为做试管嘛肯定能成的呀，然后他说如果你年轻个七八年十来年二十几岁的话，他说我再给你找找看原因这样子再给你做下手术什么的，他说你现在35岁了，他说我觉得你还是去试管比较好。以前从来都没有一个医生这么很明确的跟我说过，然后他说了之后我就决定就是去做试管了，都已经35岁了那一年，虚岁35岁，然后到了邵逸夫医院就开始做试管了！邵逸夫医院是什么时候去的？16年？16年7月份还是8月份？就那个时候去开始的，做到现在为止。

R：心理层面上自己是怎么样一个想法？

P：什么想法呢？肯定是——一开始嘛肯定觉得好像，因为我也查不出什么问题，然后一开始我老公的话么就是那个精子活力不是老是一会儿好一会儿不好这样子嘛，然后后来，后来的话感觉总是调理了之后好点起来了，总是觉得自己能怀的嘛，那会儿好像还是信心很满的，感觉，就是觉得反正已经也怀过一次了，肯定能怀的这样子，所以一直拖着就没去试管，然后后来就是去做试管了之后么总是觉得反正我都没有什么问题，我想想做试管肯定能成功的，然后我第一次取卵配了三个都是优胚，移进去之后回到家我是反正觉得很有信心，因为我觉得我好像除了内膜稍微薄一点，其他都没有问题的呀。然后结果出来没怀上，那个时候心理打击很大的，两次都没有怀上。然后第二次不是又要取卵了嘛，结果我到了取的那天竟然只取了三个，然后才配了一个，于是医生又建议我再取。就整一个过程不断的有问题出来，所以试管真的这个过程太考验心态了。那后来我老公好像对那个医院有点反感，我也有点反感，因为它整个流程就是好像就是那种上面定了方案之后，下面都是流水线一样小医生都给我再看一下。而且我第二次期望还蛮高的，能取多一点这样子，而且用的药也很贵的，第二次用了果纳芬2万块钱配的怎么下去才配了一个胚胎，那么就是而且我取的时间也给我安排在最后第二个，因为他们那边好像都这样子的，卵泡少的人先取多的人在后面，结果轮到我取了，才取了三个。那么后来想想一个又是优胚取出来，又是八细胞的，然后后来想想，那算了吧，反正我也没有打算再在这家医院做下去了，一个先移掉那还是没有成，然后就结束了那边。然后那边还说三次移植失败的人会给我们会诊什么的，结果报了名之后就什么都没有给我们做，一点建议都没有，然后你问医生吧，他们就是全部责任都推到你身上来的，就是说你们可能胚胎欠好，然后比如说你什么卵巢功能不好了，我们那个什么取卵的时间也是无法控制的这样子，你卵巢不好的，卵巢功能不好的那种病人，本来就是那种好像说时间比较难把握这样子。反正就所有的责任都推到你自己身上来的都是。然后呢我就不想去了。那三次也是没有着床的，反正也还好，就前期就这么打打针什么的，药停掉了之后还是正常来月经这样子！然后后来我在这边好像也做了个宫腔镜手术，做了两次，这边什么徐新亚啊什么，还有何医生说我好像B超单看起来稍微有点粘连什么的，然后周倩茹啊她说我子宫腔里面比较深的那个地方，她说有粘连，中度粘连，给我解开了。然后后来就是我本来我就打算去上海嘛，他们都说上海好一点，然后我就找来找去，又在上海的几家医院里面，在比较哪家好这样子。后来本来上海有几家医院在比较了，上海九院，还有什么国妇婴不是，都是在做试管的，后来上海国妇婴有个院长，他们的院长黄荷凤你知道吗？以前是这边妇保的院长省妇保院院长，然后去了那个上海国妇婴当了院长，他是什么中国科学院院士，然后是我们嵊州老家人，然后他后来也才去年开始吧他就是跟我们嵊州妇保合作吧回馈家乡那种，她每个月就是会派那边的那种专家过来到我们那里来坐诊这样子。然后有一次他就是派了一个生殖科的专家，就是做试管的嘛，然后到我们那里来会诊，会诊我就去看他。看他他就是把我前后这么几年来的那种病例都看了一下，然后他就是就感觉好像我觉得他分析的还是比较有道理的，他说我帮你看了一下，我觉得你在邵逸夫用的都是那种常规的方案，他说而且每次我们做的都是二代试管，然后他说我如果你到了上海来我这里看的话，他说我要用那种反常规的方案给你试一下，他说或许你这个人常规方案行不通这样。然后我就听听他讲的还是比较有道理的，我就去找他了。然后我到了上海之后，他给我用的那些促排的药都是国产的，还是很便宜的还，就一次取卵下来，而且他还给我做了一代试管，让我们我老公活力那天也取出来也比较好，然后娶了十个卵泡，那次最多了，我身上最多的一次！取出来，后来就是一代试管配了四个，全都是可以冻起来，就是质量都三天的冻胚质量看起来都可以的了。然后他就给我用了那种什么超长方案，就是先打那个，他说我就很久没怀孕了，很多年没怀孕了嘛，他就先给我打什么降调的针，然后那个针下去就是说，前面两个移进去之后，如果不成功的话，他就是马上再给我接着移第二次这样子。就这么一个连续移的方案。然后我前面放了，因为我会那心超做出来好像说过有卵圆孔未闭合，然后医生都不敢给我用，都不敢给我用那个什么放两个，万一两个成活了，他说要减胎，可能我心脏负担不了这样子。后来那么先放了一个进去，然后另外一个去养囊这样子，结果养囊的那个养不成，养死了，放进去的那个也没有着床。然后后来就是失败了之后月经来完马上又去进周，然后又开始打针吃药，然后又连续第二次再放进去，放进去又放了两个，我这次我说算了，养也不养了，放在外面也是死掉的，还不如放到肚子里面去，然后我就两个一起再放进去，放进去之后就是不是活了一个嘛就是这一次，然后这个活了不是又是不好了，就后面，后面不是医生说什么胚胎异常还是不行这样子。所以现在胚胎也没有了，就是B超做出来没有胎心胎芽了，然后医生说就是可能是异常胚胎，那就不行了，就这样子。大概都做了五次了。然后我老公其实身体也不太好，他是我们第一年做试管不是要体检嘛，他前面就是一直有着，三十几岁，三十一二岁开始，他就一直

有在说，他好像背比较酸什么的，背比较酸嘛到后来还去做了磁共振什么的，然后去看了，看了好像医生也说磁共振好像都拍不出，没什么问题这样子，然后后面她那种那种做试管不是要体检嘛，小便不是都要检查的，小便里查出来有隐血和蛋白。

R：肾功能跟不是很好

P：对，然后那会儿我们是不懂的，然后就觉得，好像然后邵逸夫的医生他说这个做试管呢不影响的，他说就是你们自己最好去找内科医生看一下这样子，医生觉得好像有点无所谓的那种感觉，我们也没有放在心上嘛，对，然后我们就回去老家就找了我们那边的一个中医科的医生，这没什么关系的，我这里有一个特效药，他说三帖下去就见效了，会转阴的，只不过发展到后面他说就是容易变成尿毒症这样子，那我们觉得他这么敷衍了事，又好像不放在心上，而且到时候三帖就转阴这样子，我们就，自己不懂呀，就是一直都没放在心上呀，然后后面就是那个到了过年的时候，那么就是一个下半年，我不是都在做那个试管，取卵啊干嘛什么的，然后我后来那个我老公也每次就陪我来的那会儿，然后就没有放在心上嘛，直到后来过年的时候，我老公平常也不打麻将的，然后过年么就是他们村里那些小伙伴叫他去打麻将了，然后他说好像打到后面，他那个牌都看不住了，感觉头昏昏沉沉的这样子，然后我说你要不去住院检查吧，然后后来就找了他的同学去他们科室里面住院做了体检出来，医生判断说它可能是肾性高血压这样子。然后我们就在那边一直治疗，他一直给他吃的是白令胶囊，然后也没有查什么的，他说为了让他血压先下来这样子。后来我们不是一直呃17年的时候，我不是一直在何医生那里看嘛吃中药什么的，然后看到下半年何医生说你那个宫腔镜也做了，粘连也解开了，然后输卵管也是通的他说，也是通通了一下，也是通的这样子，然后好像看看也没什么问题啊，她说要不你们回去自己先试着怀一下吧，我觉得他说可以的这样子，然后我说那我老公这个人，我说精子活力老是上上下下的那种，我说你能不能给他调理一下？然后我把我老公的那种报告拿去给他看了，他一看他说不行耶，他说你好像小便里面隐血跟蛋白不行，一定要去看好的，我说我们在看的，但是看了这么一个下半年了，我说好像医生一直给他吃这个药什么的，也不见得好转这样子，然后我说你那边有没有好的医生帮我们推荐一下的，然后他就说你去中医院去找朱院长吧，朱彩凤她说，然后我们就过来了，17年11月份的时候过来找朱院长，朱院长说你这个比生小宝宝还重要。他说，我建议你马上来住院，他说更全面的检查。后来我老公就来这里住院，住了十天，做了肾穿刺，然后他查出来他就是肾有1/3又硬化掉了就是，然后已经开始——因为嵊州的我们那个医生好像也不太懂，他那个肾功能，查出来好像是104这样子数值，然后它的最高那个指标是134，那么不是你抽血的指标里面不是没有箭头嘛，就是在它的范围之内嘛，然后嵊州的医生说是肾功能是还好的这样子，然后嘛到了朱院长朱院长就说你已经影响到肾功能了，我们说不是还在那个——他说像你这个年纪的话应该是在80以内的，然后我老公有104了已经。那么他说一定要先开始用药，用激素就先治疗嘛，后来就是治疗了之后，我老公可能对那些药什么的疗效还可以，就是一直就这么好像又降下来了，变成86这样子接近他该他的年纪该有的正常值了。然后我老公就是从17年10月份开始，11月份开始在朱院长那里看一直到现在，然后所以我就一直觉得我们年纪上去之后，可能精子啊也不太好，因为毕竟它就是这个肾不太好嘛肯定也影响。然后我们年纪也上去了，可能卵子也不太好，我就觉得我们两个人越来越难了。

R：一路过来也是真的挺不容易的。

P：然后一开始吧可能家里人都希望挺大的，后来做到后面吧自己感觉都有一点好像有点好像感觉做不下去那种感觉。然后像我老公吧可能他心里不说，他嘴巴里不说平常也不太说啊什么的，但心里他——我就明显的感觉他自己生病了之后，就是感觉心里那种，有一种焦虑症，一来呢他肯定也很担心他自己的身体嘛，他动不动就觉得自己会死啊什么的这样子。他就怕自己得了尿毒症什么的，就是做化疗就觉得活着没意思这样。心里其实很焦虑的那种。然后我嘛就是一次一次又失败了，又感觉自己又是很焦虑的也是，然后就感觉明显的两个人生活质量都下降了好多。真的做试管的话，我就觉得好像怎么说呢，身体上那种苦还是能接受的，你就这么打针什么干嘛。但是一次一次不成功，就是感觉心理压力会很大很大这样子。特别是像我们这种年纪嘛又上去，然后两个人就各种状况，像我卵巢功能下降，像他这样子肾功能不好啊这样子，就那种越来越觉得可能生不出小孩子那种感觉，有那种恐惧的心理，有那种恐惧心理就是这样子的。

R：具体就是说害怕哪些东西，哪些原因哪些点就引起你恐惧这样子？

P：那我跟我老公哦我们两个人是初中同学，我们平常都是比较很讲得来的那种，反正一直过来都是那种比较反正都两个人什么都比较聊得来。虽然我老公那种有的时候脾气比较急，什么比较急躁的，但是他还是很——就怎么说呢，该关心的时候还是比较关心的这样子，他嘴巴里是不太说的，但是那种行动上什么的都是还是比较关心的。但是你到后面啦一直都是家里一直都是两个人啦，两个人结婚都十年了，家里还是两个人，然后那种然后你做试管不是又要花很多钱嘛……

患者接了一个电话，然后——

R：好我们继续

P：就是做试管啦其实我觉得还是心理上的压力比较大。

R：对，我主要就是说想了解一下具体有哪些心理压力？跟我说的更细一点。

P：比如说一方面那你可能就是一次你比如说一两次你就能成功的话，那也还好这样子哦。一方面肯定是那种像我现在这个样子，我就是最忧虑的事情，我就是我的年纪，我不是38岁了，虚岁已经。然后年纪上去了不是试管的成功率会越来越低嘛，然后就是感觉心里有点那种很焦虑的那种感觉。感觉上了年纪之后就越来越难了，卵巢功能也降低，也下退了这样子。然后还有特别是我老公也不是很健康，然后我就觉得每次这种胚胎第三天的时候配起来是优胚。但是到了五天之后，或者放到肚子里之后，你虽然着床了，但是他那个发育潜力一直不好，所以我也是觉得肯定是我们那种我们卵母细胞跟精子细胞那种质量肯定不太好的这样子。所以好像虽然能配成胚胎，但是到底能不能成为一个人，感觉就是这方面很漫长的一个过程，你即使像我这次一样移植成功了，我就是好像心里也没有很高兴，我老公好像一直也是一种很忧虑这样子，就是怕这么漫长的一段时间能不能生下来，一直就是，一直很担忧的其实，就是那种着床的那种高兴程度，其实还是没有多少这样子。然后第二点嘛你还有一个压力，就是来自于比如说他妈妈他爸爸这样子，因为我老公是家里的独子，而且我婆婆这个人吧，你看她这么七天来陪我，来陪我的时候是很好的这样子，就是照顾的也很周到，但是一方面就是她这个人本来脾气也不太好了，她就这样子自己来就是，老是有的时候拉着个脸，然后可能是他自己心里比较烦，也不是说在怨我也不是说在怨谁这样子，她可能就自己心里比较烦，但是她就是不知道掩饰的那种，平常就是比如说我失败了，因为她前面几次我失败了，她都不在我身边，她也从来没有来照顾我，也没有看到我打针，看到我跑上海跑杭州这样子，也没看到我这么辛苦，所以每一次失败她好像看上去就是比我还还不舒服这样子那种感觉，那么肯定是我自己本来就不开心了，你还有的时候这么一个我碰到你的时候，你还拉着个脸，我肯定心里更不舒服了这样子，然后就是来自于他们那种压力也很大的。然后我们像我婆婆是住在农村里的，农村里么那种人肯定更多的话，他们有的时候很明显的就是比如说一大群女的在聊天，就说某某某家的媳妇结婚都十年了，现在还没有小孩这样子，然后我老公邻居也很搞笑，他们那户人家有点很不讲道理的这样子，上次好像为了一个什么厕所里面一个化粪池啊什么，就是两户人家城里的意思是我们我老公家跟他们家是连着墙的这样子，然后就是要选一户人家在这里挖一个就是小便池这样子的。然后他们家的人很奇怪的，就是说一定要挖在我婆婆墙角下，他说反正他们家嵊州房子有房子什么的，就是我们城里有房子，他们家就这么一个房子什么的怎么样怎么样，就是一直很来挑恤的那种，然后我公公

然后他们老是这样子也不舒服了，然后他旁边那个人那个女的就开始说了，我们家是已经四代同堂啦死了也不要紧的什么什么的这样子，那就是来骂我们家没有后代这样子，很奇怪的啦农村里这种人，那我跟我老公平常是住在我们自己家里的也不太回去的，也听不到什么，但是我婆婆就是有的时候，就是有的时候人家也看她的，可能她自己觉得自己出去别人看他的那种眼神都不一样这样子，就觉得好像你家媳妇娶了这么多年都没有小孩子这样子，她就心里也很不舒服的，然后那种压力有的时候肯定也会无形地转移到我的身上来的嘛这样子。还有么就是比如说你也有，那么做试管么肯定也还有经济压力的，那我老公自己做点小生意，那么平常自己两个人吃吃用也是够的，我自己也上班，工资也能赚一点这样子。但是你去做一次试管的话，起码3万以上做一次就起码3万以上，而且来来回回还要老是跑上海跑杭州这种费用这样子，又要请假又要干嘛这样子，而且像我的话请假已经算很好请了，因为我老板是我亲戚，他都知道我这么多年没有怀上都很理解的，每次反正只要我说一声就让我出来了反正。但是这么多年了，你说那种什么平常么又要保养，要吃那种东西，然后又要什么花费也很大的，然后我公公婆婆是反正也不支持的，就是我们自己两个人，就自己赚的钱都花在那里了，差不多这样子。如果能成功的话，其实也还好吧，就是不成功的话就压力更大。

R：那有没有想过以后的这个打算什么的？

P：跟你说哦，我就觉得好像这一次之后像我老公现在越来越，话啦越来越少了这样子。就觉得好像我都没办法跟他去提这个事情，我自己心里在想我，反正我还有40岁还有两年，我再怎么拼，我贷我去做一下贷款，我要去借钱，我也要去泰国试一下要么，泰国那边他可以那种做三代的嘛基因可以筛选的，但是我好像有的时候都无从跟我老公说起这个事情，怎么说呢…….

R：你们现在都不沟通了吗？

P：有的时候嘛也说一下，但是好像这次不是胚胎报告还没出来嘛，有什么沟通好像都没什么好沟通，但是，沟通越来越少了我感觉。

R：为什么呢？原来你说你跟你们两个很有话说的。

P：对，然后我也不知道最近怎么回事，感觉好像两个人沟通的越来越少。比如说我是一边做试管一边上班，然后我平常就是去医院，然后回来就是，那么厂里有事情的话，肯定又要去加班，要去干嘛这样子。然后我老公呢是这样子的，他自己不是每个月要来杭州看医生配中药嘛，然后他就心里哦肯定也不舒服的，他得了这个病，然后可能他自己有的时候想起来，小孩么也没有，自己么又得了这个病。然后去年，去年我老公就是说投资了两次都失败了，那不是钱也亏掉啦。然后就是压力也肯定比较大的嘛，他已经就是比如说把我们

的房子抵押出去去贷款，贷了一笔钱，本来想去赚一笔钱的，结果他亏掉了，他就可能就是心理上面可能就是越来越不舒服了。那我是这么劝他的，那我们还年轻哦，那个钱啦真的不用担心，反正再怎么样，反正可以再赚过来哦。但是他自己心里肯定也有想法的呀，可能觉得生意么也不顺，小孩子么又生不出来，自己么又生病，然后年龄么又上去，反正可能他心里也越来越，那种可能压抑的那种感觉吧。然后我的话就是看到他平常他回来不响我这个人也是这样的，因为我是本来也不是很活泼的那种，也是比较内向的，本来的话，他回来可能两个人聊聊天，聊聊八卦什么的都还聊得来，那后来就是我自己也试管我心里也压抑，他也压抑，两个人就好像就还是不要说话。他干他的事情，我干我的事情有一种这种事情，反正好像都没有什么共同话题可以聊了现在，就有那种感觉我是。

R：你们都不会把自己碰到的这些压抑相互诉说一下？

P：那反正我就这种事情嘛，我就这个试管不成功的事情。然后他有的时候是会来安慰我，没有小孩又会怎么样？有了小孩的话不是很烦的什么的。但是他有的时候又会说，我只是安慰你的呀，没有小孩回到家里来，一点生机都没有，每次回来都是冷冷清清的怎么样这样子。我老公这个人就是比较擅长掩饰自己心里的感情的，他有的时候就是自己怎么想，他也不太会真实的透露给你，他可能觉得一方面的话，可能他如果很想很想的话，他可能也会给我增加压力这样子。他就宁愿自己心里藏着这样子，不说出来，那么那你不说出来，你肯定还会表现出来的，反正有的时候我也心情不好，他也心情不好。不好的时候也没有什么好聊的感觉，反正我想还是等这次染色体报告先出来了。不是拿去北京做染色体报告嘛，报告先出来，出来再看情况吧，反正接下去要怎么样这样的。我婆婆是说了好几次了叫我们去领养。

R：有没有考虑过呢？

P：其实我老公去年就说过，他说谁谁谁跟他来说有一个小孩子，他说要不我们去抱一个来先养养吧什么的，但是我啦好像每次都是坚决反对。

R：为什么？

P：我就觉得我一直在想，我为什么要这么生小孩子？我不就是为了生出来是我们两个人的基因嘛，那不是我们两个人的基因，我养他干嘛呀我在想。我是这样子的，我妹妹去年生了小宝宝不是，有一个外甥哦，然后生出来之后我就老是看他，看他，有的时候看他像我妹妹有的时候看看像我妹夫，那种感觉好像很微妙，对。然后那你说我去领养一个，看看看看又像谁呢，不像跟我们，然后好像又不是我们的小宝宝这样子，我自己也有点想不明白。我跟我老公这么要生小孩，我就是想看到就是说我跟他生出来的小宝宝是长什么样子的，到底是怎么样一个人这样子是吧？我老是有这种想法。

但是如果我去领养一个根本就不是我们的小宝宝，那我就感觉好像没什么意思，但是有的时候我想想吧领养回来应该也会亲的，比如说我养了一只狗，我已经养了四年了，那只狗我都觉得好像我就感觉把它当我的孩子一样的。很亲它的这样子，我老公也很喜欢的，就好像那只狗有的时候，反正我上班我都带着的，我一直带着它的这样子，都把他当小孩子一样在养着，反正一直跟着我们，狗也很懂的这样子。我有的时候想想嘛领养来的话，人长得漂亮，比如说比如说什么养的亲，其实也还是一样的，亲是肯定也会亲的，但是我就是觉得有点不甘心。我就怕领养来了之后，我就没时间再去做试管了，也没有精力再去做试管，然后我的年龄又在上去，再上去，我就再也生不出来我跟我老公之间的孩子，所以我现在很矛盾。我婆婆说了好几次了已经，他前面就几年前他就有一个人物色好的，他叫我们去领养，其实这样说起来的话，我婆婆也还好的哦，其他婆婆的，有些婆婆的话可能就是说你们离离掉算了，再娶一个这样子，从这方面考虑的话，我婆婆还是比较开明的，她都不介意去领养来这样子哦。那我也想不好怎么样。我婆婆是肯定想这么想的，他们不是有一种说法吗，就是领养来了之后，一般都自己都能还上这样子。就是领子招子这样子，那我婆婆肯定是想我们领一个，然后领来一个然后自己能再生一个这样子，他可能觉得我们心情放松了，家里嘛她是这样子跟我说的，她说没有小孩子就感觉好像一户人家不完整的这样子。她可能觉得领来了之后么，就感觉家里有生机了么，然后我们两个人可能还能自己生一个这样子这么想的。我现在我都已经——然后昨天我有一个亲戚还发我，他说有小宝宝可以领养，啪一条微信发过来给我看。他们可能现在都觉得我还是去领一个比较好。

R：一路过来确实也很不容易的——呃我更想知道一点，就是你在知道你还上了，然后后来又知道小孩子不好了，甚至后面不是又做了清宫，主要对流产这一块心里比较深刻的感觉，或者说心理当时的体验感受。

P：我这次好像没有，因为怎么说，前面也已经经历了五次，已经做过五次试管，然后前面拉一次不成功，我就感觉好像伤心一下，就是感觉心里老是自己，我老是一个人很会——就是默默的掉眼泪这样子，然后把整个家氛围都搞得不好这样子。然后这一次成功了之后，我就我好像也没有特别那种喜悦感。成功了之后，我是第12天的时候去抽血的，抽出来293，然后我到第14天的时候去上海抽血的，550，然后再过两天我又去抽血了，只有800多。那么不是翻倍翻的不太好嘛，翻的不太好么我就马上找了上海的试管的医生，让我加一个克赛，然后我又找到了何医生微信，我还特意加了何医生微信找关系，何医生也给我开了保胎药，然后上海另外有妇婴我一个朋友的嫂子是那边的专家，我也找他问了这样子，然后找了之后那种药呢跟我在用的也差不多这样子，

那么我就等何医生不是她星期——十八号回国嘛，门诊我就直接找何医生来了，然后何医生说让住院这样子。那么住进来之后，就是心情感觉有点像过山车一样的，一会儿去做B超说我好像也——第一次做B超医生说这次好像也不是特别好，后面第二次做B超还是没有胎心胎芽，他说卵黄囊也只是那种似见这样子。再到血么抽出来数值又在涨的，然后我生理反应也很强烈的，这一次就是孕反很严重的感觉。老是反正闻什么都是恶心呕吐这样子。然后嘛再到后面就是不成功，你就反正这么一个坐过山车的过程这么下来，一会感觉看看激素还好，然后看看B超么又不好这样子，反正前面心情就有一种那种大起大落的那种感觉了已经。再到后面，我那个塞雪诺酮啦，雪诺酮拿出来的药渣啦就是颜色不是白色了，有点黑灰色，我自己预感，我感觉真的可能这次我觉得应该也是真的成功把握不大，其实我心里已经有准备了感觉。所以就是说不成功之后，我也就想想，反正不成功就不成功吧。我自己也已经是最尽最大的努力了已经，你说能怎么办？胚胎不好，我也没办法了呀，医生也没有办法，神仙也没办法的这样子你说是不是？我相信反正这次是我已经尽力了。所以我回去之后，我虽然这次就是说感觉好像你们可能看我会很伤心，可能我情绪还缓不过来，其实我这次是调整的最快的一次感觉。我反而比较放松，反正想想你胚胎不好，你有什么办法，而且我已经都这么帮你过了已经你说是不是，我能帮他（指小孩）的我都帮他了已经。那你反正就是说长不出来，我也没办法的呀，所以反正只能看后面吧，先把自己调整好，先调理好。如果自己如果经济实力允许的话，要么去一趟泰国要么，然后如果反正实在是反正感觉要么先去领养吧，先这样子吧。

R：你刚跟我说就这一次，还有上一次？你上一次也是流产，也是移植后有流过一次的是吧？

P：没有，我就第一次是自己怀孕的那一次，自己第一次就29岁那年自己怀孕那一年。也是流产过一次，也是做这个清宫的。现在已经是第二次了，前面的四次试管移植的话都是没着床，就是不需要做这么多那种什么清宫什么的，它就自己月经会来的嘛。（停顿了一会）但是哦我是存在这个问题，昨天晚上刚刚跟我一个朋友，也是那种邵逸夫一起的那种病友嘛，她是这样子的，她前面取的胚胎哦取的还蛮多的，有八个冻在那里，但她前面移了两个不成，移了两个又不成都没做上，第三次移植的时候两个养囊养成了之后，移进去就成功了，成功结果到了四个多月的时候，好像里面内环境不好，早产了说。四个多月就不行了，四个多月不行，然后她就引产引掉了嘛，然后她昨天跟发微信，她已经引产引掉已经四个月了，她说她现在才调整过来，她都感觉自己快得忧郁症了感觉。我就觉得好像做试管啦对精神压力还是真的蛮大的，真的要自己想开一点。有的时候想想吧真的是不理解，我除了内膜薄一点其他也没什么问题，为什么做了这么多次都没有成功，人家50岁的都能成功，我想想我40不到，现在科技这么发达，努力了总是可以的，但现实摆在眼前偏偏人家50岁的成功了，而你38岁就不成功，也没办法的。

R：别的你觉得还有没有哪些方面，主要是你自己心理压力，包括心理感受，心理体验这一块，你觉得有没有什么让你更深刻一点的比较深刻的？尤其是流产后的这种。

P：怎么说呢我觉得做试管你整个过程下来，其实人家老是问打针痛不痛，取卵痛不痛什么的，其实我觉得都是次要的。最主要的其实还是有家里人关心你，鼓励你关心你其实。我是这样想的，如果我老公跟我说，算了算了，我们不去做了，可能我也会放弃了，比如说我老公说这次算了吧，反正调理好，下次再去好了，给我一个明确的引导。这样子的话可能反正我感觉自己反正不管身体怎么样，就打打针啊什么的，我可能还是拼了命的会去的这样子。但是好像家人态度不明确，你就感觉难以坚持。那你不管怎么样，你也要给我一个，比如说你——不是我一个人在做这件事情呀，生出来的小宝宝又不是我自己一个人的，你说是不是，你态度不明确的话，我就不知道下一步该怎么走了。你打算——你到底想不想要小宝宝呀，你到底想不想要我给你生的呀什么的，你不明确的话，我就觉得好像就有点走不下去的那种感觉。所以我觉得现在还是先休息一下吧，让我老公自己也想一想，然后到时候再商量吧到底怎么样。一下子反正比如说那个胚胎结果也还没有出来，到底怎么样。但是上次跟我一起住的那个人哦她就是很坚强的，她就是自己赚钱。她做造价工程师，他可能年薪还高的，他所有的钱都拿去自己，他都是自己出的钱拿去做试管的。他就是一次一次的自己一个人去上海取卵，取卵移植，她不是做了八次吗，她成功啦做了8次了，她是很坚强的的，真的，她老公跟我老公不一样，她家里的人吧她公公好像就是态度有时候不太好但是也偶尔这样子，他们家也很要小孩子嘛，可能没有的时候，她公公有时候也拉着个脸，然后她老公呢感觉自己顾自己的，就怎么说呢，好像她自己所有的赚的钱都花在试管上，她老公赚的钱说花在车子上房子上，他们两个人这样，他们所有的都是分开来这样子。然后她老公是这样子的，一天到晚就是说什么事情都会来问她这样子的，但是呢你说让他来陪她或者贴心的事情给他做，好像倒是也不太有的这样子，拿件衣服都要拿个两三天才会帮你拿来这样子，就是每个家庭不一样的，然后我老公呢是这样子的，要他微信发你电话打你老是跟你说好话，跟你一天到晚聊日常，他都没有这个时间跟我来瞎聊的这样子。他就是自己很独立的，比如说我不在家，他也反正把自己家里弄得孩子整整齐齐地的这样子。

R：能说说病友间的人际关系感受吗？

P：我觉得做试管的女的，或者经历不孕不育的朋友，都是很热心的。就说一件事情吧。我去年年前移植，正月里验出着床，我隔天去查翻倍，但是2月14日那天，傍晚发现翻倍有点卡，我问了试管的医生，让我打肝素，大晚上的，我们小县城都买不到肝素，我们小区以为朋友曾经也是在上海一妇婴胞胎，用的肝素，我就联系了她。但是她家里没有，又帮我联系了她的朋友，那位朋友是我们人民医院的护士，也胞胎用过肝素，然后情人节的晚上，她取消了约会，让我去她家拿，还帮我打了，还教我怎么打，要注意什么问题，真的很感动。

R：所以你也因此交了不少相似经历的朋友吧？

P：对呀，这几年试管，交了好几个朋友。大家都是在试管途中认识，都有着相似的经历，可能会一起经历做小手术，取卵，移植，可以交流自己的感受，怎样吃药，怎样打针，分享什么运动对身体好，吃什么东西有助于卵泡成长，有助于着床等等等等，很多事情可以交流。然后真正觉得志同道合的，还可以分享家庭生活。但也不是每一位试管认识的都成为好朋友，试管本来就很苦，也是真正聊的到一起的，才能一直交往下去，成为好朋友

R：因为苦所以特别能理解是吗？

P：嗯，因为大家都一样的经历，所以更加能聊到一块，能理解感受。没有试管经历或者不知道这个事情的人，不太能理解这方面的感受，她们也不懂你的感受。所以别人面前也不想说起这样的事情。有相同经历的人就不一样了，什么想法都能敞开聊，她们懂。还有，其实很多姐妹有点不想让别人知道自己试管的事情的，可能特别烦的时候，又不能和同事，和朋友诉说，但是在试管的朋友面前，就什么都可以聊，也算是解压的一种方式吧。
