# Supplementary material for: An insurmountable obstacle: Experiences of Chinese women undergoing in vitro fertilization
Source: PLoS One. 2024 Oct 7;19(10):e0311660. doi: 10.1371/journal.pone.0311660 (PMC11458033; doi:10.1371/journal.pone.0311660)
Supplement: S1 Data — (ZIP) [file pone.0311660.s001.zip › data/P12.docx]

想简单了解一下就移植嘛了解一下你的心路历程。

移植就是20年——不是我胎停嘛，然后下半年就一直就备孕，可能也是我自己跟我老公，因为我们是经人介绍的，所以感情基础也不是很好，再加上我不是胎停以后心情也一直不好嘛，所以下半年虽然说有备孕，但是可能在一起的次数比较少，所以一直没还上，然后2021年我就着急了。然后我表嫂是就是在省人民医院做的移植，就一次成功的，他就建议我去看看生殖科，然后就是21年的3月份就去生殖科看了，那边那个苏主任也是建议我移植，呃我就开始移植加备孕嘛自己备孕，我是好像三四月份去检查完了以后，取完卵以后，又自己又怀上了，怀上了呢又生化掉了，就不知道什么原因就是21年就是两次都是生化，一次是自己怀的，下半年一次呢是移植，第一次移植也是生化掉，就一直就是好像HCG不太好，就是从21年开始，20年一次就是胎停，21年是2次，今年一次又是胎停就相当于4次了，反正就是不太顺利，就都是每次都是说我HCG不好。

有没有查过胚胎染色体？

呃第一次是那个就是省妇保的时候是就是出血了，然后我挂了急诊，急诊医生他就给我做了个B超说已经快掉出来了，然后他也没让我住院就让我回去了，他说自己会掉出来的，他也没跟我讲这个胚胎要带回去，那个所以没查。然后这一次胎停呢是因为还很小，就看不到，就很小那个做B超做出来就孕囊都还没有吧就是很小的

你跟你老公染色体也都查过的。

我们是之前就是要做那个移植嘛，不是各种检查都做过嘛，染色体都查了都好的，应该是20年那个胎停以后，省妇保就给我们查过染色体的问题，都好的，

所以之前总也很那个的，原因找不到

对原因找不到，然后省妇保查的时候呢那一次呢就是查出来说我有胰岛素抵抗，nk呢就是临界线，好像是第一次查是十九点几，第二次查是二十点几，那这次的话不是打挂了三组蛋白嘛然后我要求这里给我查一下那个 nk数值，因为我一直担心我nk高是不是导致胎停就是不着床的原因，后来查一下我nk只有只剩下8.1了，那说明蛋白对我来说应该是有用的，把NK降下来了吧。那应该不是nk问题导致的，所以也不清楚是什么原因。胰岛素抵抗呢是去年上半年3月份查的时候查出来说我胰岛素抵抗，后来我就一直减肥，然后再运动，嗯后面查了几次血糖也都好的，所以也不确定是不是胰岛素抵抗导致的，就现在就不清楚到底是什么原因导致的，查不出来，因为之前就是可能会导致不着床，就nk要么胰岛素。。。

R：其实我主要想我我这边啊主要是想了解一下就是你的就心理上就你的心路历程是怎么样的。

P：就是不能说这件事，一说这件事就整个人就崩溃掉了，所以那个状态真的很糟糕，因为就很——好不容易怀上，然后那时候已经其实有胎芽了，但是还没有胎心。然后就很期待就是有个自己宝宝嘛，因为毕竟年纪大了嘛，好不容易怀上就很期待.那个状态确实是很糟糕，就半夜里经常是哭醒的。

R：那次是自己也是移植的？

P：自己怀的，因为我自己年纪大了，好不容易怀上了，那时候都有已经有胎芽了，我是觉得自己会可以有这个宝宝的，当时是真的很想要宝宝嘛。那次就——第一次就特别崩溃，后面就是生化我倒觉得还没有那一次那么崩溃。这次又——又有点就是，胎停好像对我来说就比生化对我来说影响会大很多，像这次我也是很期待很期待的，因为毕竟是第二次移植了，然后前面三次HCG查都还挺好的，翻倍都可以，那我之前就查HCG都不太好，就这次HCG前三次是好的，所以也是好像很期待就觉得有希望的那一种。再然后就那天不是说去查了B超就没有长大嘛，就这边就去查 B超之前医生也跟我讲了，他说你基本上因为查了几次，那个就HCG翻倍不好，他说基本上就可能胚胎不长了，然后去做了B超也确实是没有长大。所以那一天我就在住院部，那个电梯门口就不行了，那边护士还挺好的，就安慰我了很久。就可能胎停对我来说比那个生化——就整个人不行，这次比上次就是20年那次胎停好一点。

R:为什么这么难过？

P:我不知道就控制不住自己，

R:心里想什么，

P:就觉得第一次是觉得自己好像呃——不够，因为当时就去了妇保，因为刚好疫情爆发嘛，其他地方去不了。我是2020年的元旦过后没几天查出来怀孕的，不是快过年了嘛，那时候不是刚好疫情爆发就去了省妇保，省妇保说不用吃药了，我也听他话就不吃药了。我就好像有点自责，因为如果说我多去几家医院或者来这里保胎，可能就不会出现胎停的情况，所以那个就很自责。

R:第一次的时候觉得自己保胎不够位。

P:对，就对第一次我就耿耿于怀，昨天还看到很多小孩子我就在说，就是第一次的时候，如果说我保他保住了话，我们宝宝也也可以跑来跑去了。确实第一次那次我会影响比较大一点。

R:主要就是希望比较大，

P:对，然后再加上就是可能自己确实是很想要，一个是很想要，第二个是自己觉得没有去多了解一下就是保胎这方面的相关信息啊，然后自己可能也没有——那时候没有太在意就是保胎这个事情，就觉得自己怀上了就可以平安地生下来了.

R:就觉得自己不够重视？

P:对，然后直到他说HCG，就是一直跟我说HCG不好，然后我就心理压力负担比较重，然后就之后，就但是心理压力重，但是我又没有去找别的医院去（指保胎），所以我就觉得自己那时候好像并没有为这件事情就是承担这个（指尽责）。

R:心理压力很大？

P:那时候心理压力确实很大。

R:有哪些心理压力？

P:呃怎么说呢就觉得自己没有像其他人都会去尽力保住孩子，去各个医院去找医生去咨询啊什么的。然后我是那时候太相信了，我是觉得妇保不是很专业的妇科医院嘛，那医生说什么我就信了。我如果说多问几个医生可能情况就不一样了。

R:你是说你胎停以后心理压力很大？

P:是胎停以后心理压力太大，对就这一块就觉得自己自责，另外好像也还好

R:家里人怪你吗？

P:我公公言语上面有过一次两次，那个我老公不会。

R:你公公直接说？

P:也没有，他是觉得那时候就是我怀孕之前是有工作的，但是工作有时候比较忙，可能到晚上12点就上班到晚上12点，时间又是年底又很忙，所以他就觉得我整天面对电脑，可能对生宝宝有影响，所以说过一次两次，其他人都没有。

R:那其实主要还是你自己给自己的自责

P:主要是家人都不太会给我压力，都让我放宽心，他说都说宝宝会有的嗯，但我自己就是对第一次就有点耿耿于怀。然后面就是移植我是就去了以后，我是觉得自己有点着急了，因为那时候取卵的时候，之前不是说有点nk高，有点胰岛素抵抗吗？那个我觉得我状态还没有完全调整过来就取卵了，我是在想是不是这个胚胎的质量也有问题

R:自己在找原因

P:对我就自己在想，就是那时候还缺维生素d，那维生素d那个剂量是比较低的，也不可能一下子补上去，所以我在想是不是这个也有关系，所以我自己不懂，然后呢又看了很多相关的东西，然后就在各种怀疑是不是这个什么原因导致那个原因导致，因为说句实话，没有人能告诉我到底是什么导致的，我一直就是 Hcg上不去导致或胎停或者是什么问题，甚至我觉得可能怀是我自己能怀上，但是就是那个保不保得下来的问题，

R:那后面的话你是做反正没做的，但他自己有药流吗？还是怎么药流？

P:吃了药吃了药就是前面第一天没有出血，第二天就是基本上都流完了就出血。

R:别的方面呢对自己的平时的那种人际啊社交啊这些方面有影响吗？

P:有。就胎停以后再加上去年生化就不太愿意见人。

R:为什么

P:不知道，就村里面就七大姑八大姨的就言语会比较多一点，然后我就不想，就屏蔽掉这些，再我自己可能也比较喜欢自己在家种种花啊看看书啊什么的，就也懒得去这些社交了。我们是农村的嘛，然后自家的院子铁门一关，就自己一个小院子里面。

R:那你们那边会说来说去给你听到吗，还是怎么？

P:有，有，对，有一段时间去年吧应该是21年就是那个生化以后，那之后我就是经常会跟我老公在村子里面就是散散步，就偶尔也会听到就是一些(闲言碎语)，再加上有时候他们走到门口了，那么可能会聊几句，他们就各种催也有，因为我不太去跟他们讲我的事情，他们也不知道我在移植也不知道我怀过孕，也不知道我情况，就在那里催，他说你们好要孩子啊什么的，我就不太愿意——因为那一次生化倒还好，就之前不是胎停那个状态就不好嘛,就所以我一直就不太愿意听到这方面跟他们去说这个事情，也不愿意听到这些事情，所以我就不太去跟村里面人接触，而且我觉得接触了也毫无意义，他们只是会给到我压力，也没帮到我什么东西，那我何必去跟他们接触呢，所以我还不如自己种种花或者做一些自己喜欢的事情。

R:上班还在上班？

P:我把工作停掉了，因为去年不是移植嘛，就是经常跑医院，所以也不方便，所以我就把工作停了。

R:那你现在就专心弄这个事情，

P:对。而且我这个年龄就是可能体力也跟不上，因为我们之前那个工作就是工作强度比较大，嗯有时候忙起来的话，下了班就整个人摊在那里，就有时候累得想吐，就集中精力的那种，所以也其实也有点累的，所以那时候我还在想是不是第一次胎停跟我工作压力大或者累到了，但是那那个其实我觉得我查出来有孩子就过了没几天我就把工作停掉了，所以我在想是不是也问题影响不大。

R:后面你是怎么进行调整的？

P:调整就是我自己自己慢慢地消化

R:通过哪些方式？

P:好像也就时间吧，然后就自己因为比较喜欢种花花花草草，然后就早上起来看看花花草草，就心情会好很多。就有一段时间之前还会听听音乐看看书什么的，就胎停以后就状态不好书根本就看不进去，也只有就是好像动起来还好一些，我即便是有时候，就是我有时候心情不好的时候会搞卫生，但是那次以后我不知道为什么就搞卫生的时候都会胡思乱想，就想想什么都不知道，就是乱七八糟的，很多思绪。

R:想哪些东西呢

P:我都不知道我自己想什么，对，就胡乱想，翻过来翻过去的，就这样子，反而是那个就是倒在院子里种花种草，然后那倒是稍微好一些，其他好像也没有，以前还会去旅游旅游，就是前面那个第一次胎停还出去走走，去外面走走去旅游了一次，然后那个还好一些，这两年都没出去旅游，我觉得更压抑了。

R:怎么安慰自己。

P:也没有，就尽量的——我老公说不要去想过去了，就不要去想了，他说实在不行我们领养一个都成的，就我觉得我老公对我还好，再自己就慢慢消化，真的是时间

R:那其实总的来讲在这个总的压力来讲也其实很多时候是你自己给你自己压力

P:就是我自己，因为是我其实我老公倒是没有像我这么着急，包括去要去做移植都是我要求的，因为我着急我想要孩子，包括那个后来就是各种调理都是他好像他就觉得就顺其自然，有最好没有那领养一个也没问题，之前有聊过这个事情，那他倒是还好，但是我就是自己就很想要一个自己的孩子，就可能我的想要孩子那个心情比他迫切很多。毕竟男的我经常觉得男的可能这方面生育方面压力没有女性来的大。

R:他可能对孩子一般没有很喜欢的那种

P:可能跟他就是成长的那个经历也有关系，那可能我是因为一直以来我都很喜欢孩子，所以就结了婚，我就觉得有自己的一个孩子是必须的那一种，所以就很迫切想要。他那倒还好。

R:有没有一些让你感觉到比较积极的一些方面啊什么的？

P:想不出来，

R:平时有没有像病友之间的交流啊这一类，

P:这会有，交流可能少一点，我不太在群里说话，但是我会去看他们聊天。我不是在家有时候也无聊，然后我会去刷一刷他们之前的聊天记录，看一下什么东西对自己身体好的，有利于助孕的，有利于或者保胎的，或者对宝宝好的，对，然后也会去关注一下哎为什么会生化会胎停啊什么的，也会去看一下。

R:那么从你一开始一开始接触移植，到现在相当于移植了两次了，就有没有说对移植就从开始进来的想法跟现在的想法一样吗？

P:那时候就着急就想着可能移植是不是成功率会更高一些？那现在看来就跟我自怀其实也相差不大，就是一个几率问题了，也不一定说是一定能成，或者说我当时觉得呃因为不了解说就是胚胎优质的胚胎，我以为就是最好的，是挑出来，那后面我了解的多了，优质胚胎它只是形体上面看起来比较好的那种，挑出来形状好的，这并不能代表它质量是最好的。所以我我在想那跟我自怀其实一样的几率嘛，那我就觉得

我是不是需要剩下的两个胚胎是不是还要移，就有一点纠结了。因为我做过宫腔镜就是里面就是有点炎症，然后当时是有一个什么就里面有个宫腔里面有一个很小的息肉，然后粘连都还好，输卵管也是畅通的，也是说明是可以怀的，那我自己也怀过两次了，那说明对我来说可能一代的移植跟自怀是没什么区别，所以好像也就没那么期待再继续移植，如果说实在不行的话，如果要移植的话，也估计要重新取卵做三代。我是觉得我可能怀是可以怀，就在于怎么把它保下来，我的重头是在保，所以我就纠结要不要再继续移植这个事情，

R:就一开始你觉得移植可能比较简单，也可以帮你去解决这个问题，然后现在发现并没有。

P:是的，而且那时候因为第一次经历心理压力也会很大，而且对——因为取卵啊什么的，毕竟是一个小手术，那之前都没有做过任何手术，就很害怕。然后就很害怕，然后取卵也好，移植也好，我就前面一天我就几乎不能睡觉我就紧张很紧张很害怕，其实也还好，就是我自己心里害怕，可能是对未知的一些东西的害怕，因为那时候都没有经历过，所以就心里就觉得恐惧害怕，然后紧张，包括我后来做宫腔镜，我看旁边那几个女孩子都宫腔镜做完状态很差，我倒是还好我都还好。对，没有那么的敏感。因为我宫腔镜做的，那当时我还说是做全麻还是半麻什么的，然后后面好像是局麻，然后我倒还好，所以就是对未知的一个恐惧就当时因为没有了解，也没有经历过，所以就有些害怕。

R:你会跟你的朋友身边的朋友这种交流这些吗？

P:极少，就一两个比较要好的会偶尔聊起，

R:就相当于你的这些情绪，你就是能跟老公说说，

P:跟我老公聊的也不是太深入，就基本上靠自己消化多一些。

R:那会压力大一点，

P:对，因为我老公他其实他觉得什么事情都 ok没问题，他说不要，他没有那么——他也不会去深入了解，也不会好像就是没有那种太多的感同身受吧应该说。

R:你觉得人家不能感同身受，所以你就不聊了。有别的原因吗？

P:一个是我觉得他没法做到感同身受，包括朋友，偶尔我会聊一些，但是就聊的比较少一些，因为我也不太想把自己的负面的那些情绪带给他们。因为他们现在都是有孩子有家庭的，每个人都有自己的压力，那我跟他讲的这个他其实也没有太多的这方面能给到我意见什么的，因为他们根本就没有经历过这个，反倒是我表嫂，就是建议我去移植的，她倒是有时候会跟我讲讲，因为她也经历过，她可能也会了解一些，倒还好，有时候跟她讲她还能给到一点意见，但是讲的比较少。

R:那跟病友间会分享这些情绪啊什么的吗？

P:会（情绪比较高昂），上次住院保胎的时候，那个室友就第一个室友挺好的，她跟我年龄也差不多，经历也差不多，所以会聊的比较投机一些。她也是去年经历一次胎停，就情况比较接近一些，就聊得挺好的，然后也会相互哎怎么对身体好，怎么对住院什么保胎有效有用，或者说什么对——就是包括是吃什么东西会好一些，都有聊。

R:我觉得这也是一个积极的方面，

P:对，就是聊的人也会感觉开心一些，因为找到了共同话题就有人能理解你了。就跟朋友或者跟老公，我觉得他们好像就听你在那里说，但也没有实质性的能可以相互交流的一些东西，因为我老公属于那种你拨一拨他动一动拨一拨动一动，他不会说主动也去看看移植会需要什么的，需要吃一些什么东西会比较好什么的，没有。大直男一个，所以也就是说的聊的少一些。

P:我自己也知道就是后面保也未必能成，但是我没有去做，那又是另外一回事，所以我会有耿耿于怀。我清楚就是道理我是明白的，就是我当时去保了，或者说后面也有类似情况，我也保了，也没有成，那第一次也未必会有好的，但是我就觉得不行。
