# Supplementary material for: An insurmountable obstacle: Experiences of Chinese women undergoing in vitro fertilization
Source: PLoS One. 2024 Oct 7;19(10):e0311660. doi: 10.1371/journal.pone.0311660 (PMC11458033; doi:10.1371/journal.pone.0311660)
Supplement: S1 Data — (ZIP) [file pone.0311660.s001.zip › data/P4.docx]

R：怎么想到去做移植的？

P：就想做试管，主要是说一个是年纪大了，当时觉得因为我就是正经地开始备孕时间也比较晚，大概是32岁的时候，然后边工作，然后边备孕。一开始呢总觉得这是一件很自然而然的事情，后来就发现没那么容易，然后后来到34岁的时候，我就觉得因为35岁相当于一个高龄产妇的临界的年龄嘛，我就想干脆我就做试管好了，我不想再等了，那么我老公的话他那个精子质量也不是很好，当时如果说再等他调好再怎么样，就是等我再怎么就感觉好像等得太久，主要是35年龄卡在这里，所以说就试管好了这样子的，然后就去做，对，然后呢结果后来呢也确实没想到试管做起来其实也是没那么容易的。它这个像我的话前面已经做过两次了，都是胎停的，而且就是说着床啊什么都没有问题，去第12天抽血血值也很好，感觉也正常的，但是就是到后面——到了移植35天左右，看起来就不大好了，然后血指尤其是雌二醇就上不去了，胎停了嘛。两次嘛你伤心嘛肯定是有的，但是就是说我也没有说特别的沉浸在里面说好久不能自拔，就——就觉得反正它已经是既成事实嘛也就接受了。然后——后来的话，因为我还有其它的胚胎在嘛，所以就说那就继续调理身体再移植这样，反正我的想法就是说，比如说我拼到40岁，如果说有那么最好，如果说没有，那么这件事情对我来讲，我已经尽力了，对吧？就是没有遗憾而已，然后你说至于而且我现在的话对有没有孩子这个理解的话，就不像原来刚开始备孕的时候那么的——呃急切吧，现在我就觉得一个是孩子，他确实是也是有缘分的，对吧？到你有的时候你就有了，肯定是该你的就是你的，不到你有的时候肯定要经历一些波折的，对吧？然后另外的话么也觉得就是说人这一生的话也不是说必须有一个孩子，然后就才意味着说你后面的生活就很幸福或者怎么样，不是这样的，你最终幸福的决定的这个人还是在你自己嘛，有孩子或者没有孩子，这种生活都有他自己的优缺点，对吧？无非对我们来讲就是，你选择一个当下，就比如说你选择我就是没有孩子了，OK了对吧？无非就是说可能到年老的时候，比如说假设就是夫妻两个，有一个人走掉了，另一个人会比较孤单，这是最难受的时候，对吧？那么之前的话我们肯定也有很多的很多优点，比如说操心的事情也少呀，或者是吧，如果是有的话当然好，总之就是说不去纠结了，所有的生活都有它的优缺点也都有它的那个。

R：您刚刚跟我说，一开始你也是比较急切的想早点怀嘛，然后现在你反而是这样一种观念，为什么会有这么大的转变呢？

P：一个是你年纪也大起来了嘛，其实最主要的转变是什么，就是我后来调理过程当中也是机缘巧合，然后去做艾灸嘛，然后艾灸馆的话它是这边也有中医什么的，然后那我说一句

话可能你不一定认同，但是我认为是认同的，就是像中医院的很多中医的话基本上都西医化了，为什么？大家主要也是看指标给你用西药或者怎么样，但是实际上有很多地道的中医的话，他其实不是这样子的套路的，他会给你讲人体它是一个——就是一个整体性，人体是有自我调节能力的，你的经络也好，或者说你的什么五脏六腑也好，如果说你功能很好，经络通的话，你有很多的问题，再加上你保健意识，就是该防风的时候要防风等等的生活习惯，比如熬夜等等弄得好的话，你自己的身体是有自愈功能的，所以就根本不需要说太多的外力来干扰他。所以而且再加上我也了解到，就是说我感觉到其实人生就是无常的，那既然说人生是无常的，其实，而且人生也没有完美，所以的话就大家都如此，是吧？每个人都有他自己缺憾的地方，所以既然如此的话，没有必要去纠结，就这样子。

R：现在这么想？

P：对，就这么想的。就这么讲，其实就是说我就说人生这段路，你其实要做的就是你看好前面可能有很多路，那我们是说孩子这件事情上可能会没有孩子，对吧？很多人他有可能结婚这条路上他就不是很顺利，是吧？还有可能我就选择单身，或者说我就选择丁克或者是怎样，不管哪条路你选定了，然后呢你能够真实的去接纳，选择这条路以后，就是我可能将来面临的这些挫折，然后我也能尽情的享受选了这条路以后他其中欢乐的那部分，坦然接受就可以了。是吧？不就没事吗？是不是？你说你很多那个家长生了孩子以后，然后那孩子教育的也不是很好，最后就是啃老的啃老，或者说有其他更好的事情，那你说他的晚年幸福吗？并没有，对吧？所以而且就是说人呢也不是说不要指望任何人，包括你的孩子说给你带来幸福感。这个孩子生下来以后，他就完全是一个独立的个体，你唯一能做的其实就是说你总想就是说把你最好的给那个小孩，让他少走弯路，少受挫折，实际上可能吗？不可能的，你就再把你更最好的经验告诉他，他自己没有去经历，他是理解不了，他也不会去听的，所以他该走的弯路，该碰的壁一个都不会少。所以你说，所以我就说实际上你作为你家长来讲，你唯一能做的就只能是说，应该说你不远不近地看着他，是吧？然后需要帮助的时候你扶一把，然后你还不能太近，太近以后你会觉得你会觉得你自己压力很大，就觉得他怎么不听我的，他这个挫折了怎么办，是吧？自己就无比焦虑，也不能太远，太远的话我们关心不够，对吧？所以呢我就觉得教养小孩什么，这是其实也是一个大人和家长自我修炼的一个过程。反正不管怎么样吧，总之还是就是说，只要我觉得是只要人对这个人生他能够有一个比较清楚的认识的话，就不太会纠结于——就是说我就有没有孩子也算一种得失，对吧？一城一池的得失，我觉得可能就不纠结，他会尽量在自己能把握的当下去做好自己能做的事情。

R：能不能多跟我讲讲就关于移植给你带来的影响？

P：移植对我带来影响。心理的？

R：对，包括移植失败，两次的流产。

P：呃——我就是觉得我就是觉得怎么讲呢，一个是肯定是尤其第一次的话满怀期待，结果突然之间意外就没有了，当时就突然才意识到，原来还有胎停这件事情，并不是说你或比如怀上了，这就是一劳永逸了，没有的。

R：意外？

P：对，然后呢第二个当时也只是说算了，因为我后面还有好几个胚胎，然后再来吧对吧？然后后来第二次以后呢是觉得这件事情怎么就是感觉它是一件事情，就这件事情如此的不顺利，让我觉得有点受挫感，然后但是呢还没有说特别的焦虑。让我焦虑的是我去做艾灸的时候，艾灸的这边的中医呢，他们是，就打比方我现在看的中医我理解有可能他是觉得你全身的气血可能不够好，但是我用药我可以有办法就是帮你的子宫的内环境的气血好起来，然后帮你进到怀孕，然后我艾灸的那边的医生他是他认为你全身的气血还不够好的时候，实际上是很容易发生，比如说大月份胎停或者是怎么样的，还是希望说你尽量多调一调再去移植，但是就是我自己养血是很难的，总是比如说推了半年又推半年又推半年，就是因为首先排除了他们故意，比如说想让我多调一调，然后他多赚点钱这个原因它应该不存在这种原因，然后他也确实我自己我也感觉到我气血是弱的，这个确实是，所以这件事情让我会觉得怎么一而再再而三的拖，就是心情会很焦虑，总觉得更把它变成一件事情，就是我不是说期待有了这个孩子以后我多么的幸福美满，而是我觉得这么多年了这个事情干不出结果了就这样子，其实我对孩子很多时候不是情感上的需求，是一件事情，就是我要结束这件事情，然后我去拼工作是这个意思，我并不想把生命浪费在这里。然后这次的话就是移植完之后，至少目前我感觉还是应该是比较好的，然后前面两次的胎停我自己也找了找原因，我感觉应该是找到了，后面怎么做，大概也有个思路，所以现在呢心态也会比较稳一些，这样子。总的来说没有说太痛苦吧，主要还是就这样。

R：那移植所引起的躯体伤害有没有给你带来什么心理压力？

P：肯定有一点，但是所以我就说也花了一段时间去调理嘛，做艾灸，基本上每天都去，那么我自己也是感觉到这个身体是有好的变化的，对，然后再加上我是感觉就是说可能缘分到了的话，因为有很多人这么讲，缘分到了的话再弱的气血该有还是会有的。所以我现在好像并没有那么忐忑，而且我现在倒是不希望这种事情发生，但是我想如果说真的是有难以避免说又那个什么了，反正该怎样怎样吧，因为你纠结这些，真的没有用。

R：你比较坦然

P：对我比较坦然，我尽力了，就这样。这件事情，谁都不是神啊，不可能所有事情都如你愿，尽力就行了。

R：那有没有别的方面的压力？

P:没有。

R：比如家里人——

P：完全没有。真心没有，就是我老公他们家里还有个弟弟已经有两个女儿了吗，也都挺大，然后我公婆的话他们也就是挺开明的，不是说什么一定要你们一定要生个孩子，甚至我婆婆刚开始都说我老公的精子质量不好的话，要不然用就是别人的精子，我是坚决不同意的，我就觉得要么我就不要孩子，要么我就是抱养一个跟我们完全没有任何血缘关系的孩子，我是绝对不会用别人的，绝对不会，我婆婆的意思说那好歹还有一半是咱家就是说是我的。但是我心里有这个洁癖我接受不了，对是的。然后他不是那个可以用非父精子嘛是吧？然后后来当然没那么惨，我们配型配了有十个，就是7个优3个普还可以的，对。反正尤其呢我父母家里的话，他肯定也不可能，更不可能给我施加什么压力，反正现在他们都是他们更坦然，就觉得没事，有就有，没有的话就是我们抱一个怎么样的，但是说他们不是很——像我妈，她不是很接受就不要孩子，我的想法是如果没有的话，我可能就不打算抱养。就两个人这样过好了，我老公也是这个想法。他就觉得两个人过也挺好的。然后我妈可能还不同意吧，但是我觉得还没有到那个份上说一定要去讨论这个问题到底抱不抱。而且我觉得这次应该还好的。

R：对，心态放好蛮关键。那就是关于移植失败以后就给你带来最大的心里感触是什么？

P：最大的心理感触啊，我要说这些，可能我觉得做下去，感觉人啊该什么年龄就要做什么样的事情，千万不要错过，否则的话，后面你要花千倍百倍的努力，然后你来弥补他，反正是要付出更大的代价，就是这样的。

R：是不是以前有经历什么？

P：没有，我们是什么情况，就是外地人嘛到杭州来工作的话，当时我跟我老公事业心都很强的，然后再加上我是设计行业嘛就是挺累的，经常熬夜加班通宵的，然后再加上我的身体可能就是气血确实从小就不是非常——

R：但我看你脸色挺好。

P：是的确实我也发现好像好神奇就是照镜子看见——哈哈哈。好像现在还透亮一点，之前好像没这样。反正总之——跟你说哪了？（开怀大笑）

R：就是说你们两个人年轻的时候比较拼。

P：哦对，然后当时我们就记得二十八九岁那会儿，我们的朋友一起都生孩子了，然后我老公还觉得哎呀你看他们多傻这么早就生孩子，我们那会就完全不想要，而且我也觉得我要生可能也就是30多岁的考虑就行，所以就是按照这个32岁去考虑怀孕的，然后结果就没——后来发现就很难，那么我现在知道原因是什么，因为我这个生活作息，包括我老公是地产公司，我是设计院都是房地产这个行业都很忙，然后高强度的这种加班很多，再加上像我的话，尤其是通宵，就像你们上夜班一样，这种你应该知道，对，而且是经常有。对这种是很伤害身体的，然后再加上我自己有的人可能身体他先天底的好一点，我可能肾气先天就弱一点，就耗的比较多。然后再一个就是说你缘分不到嘛就这样。所以，反正就是说比较坦然，但是我为什么说该什么年龄就做什么事情，我是在想假设如果说二十八九岁的时候，那会儿就像我跟我朋友一样也生孩子的话，至少我那会儿例假的量各方面什么都还蛮好的，肯定气血要比现在你看十年以后要好很多对不对？他哪怕无意之间有了肯定结果说不定也是好的，对吧？但是这种——这种总的来说，但是当时就没那么成熟嘛，就觉得刚毕业好像自己还完全没有做好任何思想准备，自己还没有长大，你就去要一个孩子。工作啊然后家庭啊什么的都没有一个着落是吧？房子啊那会就想房子车子都没有好，怎么样怎么地的。然后反正就是说，所以我就说你要单纯的从这个人，人你要遵循它的自然发展规律，我在看黄帝内经嘛。

R：你对中医很有兴趣，我看得出来。

P：还有一句，对，黄帝内经嘛，所以我就说你要根据它那个规律来，对吧？（停顿一会儿）所以你看聊不出什么太痛苦的地儿（放声大笑）。是没有，我是没有那么痛苦——

R：那就你自己想呗，你就觉得哪一点对你印象比较深刻。你就想到哪里就跟我讲。

P：就是悲伤的这个事情？

R：也不光是悲伤，不一定是悲伤，就是你觉得给你造成的影响心理的影响，悲伤也好，别的感悟也好。

P：反正这件事情给我最大的感受就是，（笑了一下）我知道到底人是怎么一回事情（大笑）。。。

R：就是看透了是吗？

P：对，对是这样子的，就是包括人体包括人世间这件事情什么的我就看得很清透了，对，我就放下了很多以前想不明白纠结的部分，然后还有执着的部分。

R：那是从哪件事情以后突然你是突然间这样子放下了呢还是一点慢慢放下的？

P：慢慢的放下的，调理我就是说艾灸调理这是个契机。

R：那你到底是因为移植失败这件事情然后给你引起放下了呢，还是你去做中医治疗艾灸——

P：我觉得是中医，因为为什么我是一个就说好一点事业心比较强的人，所以我要早点结束这件事情，然后去拼工作，我是这样的人。然后呢所以我说之前我为什么说我一定要到30多岁我才去怀孕，因为那会就想我这个工作，岗位职位我要到一个平台之后，然后我再去生孩子，然后说回来以后我依然在这个平台上。而且后面有了孩子以后，你要肯定有很多精力要放在孩子上，那么我依旧在这个平台上的话继续往下做会更好一点。其实就这样，我老公更是，我老公他这个人他事业心更加强，然后所以呢——再加上我比较强势，我现在好一点，现在好一点，以前是很强势的。那么就是说起来就是说如果老婆比较强势的话，怀孕这件事情也不是很顺利，好像是这样子。

R：都说强势生儿子的会多一点

P：嗯生儿子多一点，关键我跟你说，前段时间很奇怪的，我就想问一下就是那个叫什么——成为在家修行的居士要怎么弄嘛，然后后来就——不是这就不影响你正常生活的，因为什么，除了中医以外，我发现佛教原来是这么好的一个大智慧，他不是说迷信或者什么，它是一种智慧生命智慧，所以现在我就说对我触动最大的不是那件事情本身引起的伤痛，是应该说这件事情给我带来的挫折，然后这些挫折呢让我而且我本身从小我是一个手纹特别乱，就是想法很多，我很小的时候就在想人活着到底是——有什么就是目的或者怎么样，很小都在想这方面的东西，所以然后这件事情就彻底的让我感觉到我以前迷茫那些东西都找到了答案。就这样子，是的。

R：那你现在是透彻了对不对？那你总有没有透彻那时候的吧，那时候是怎么想的？

P：对，当然，相对迷茫。但是那会不是因为孩子，那会儿都是工作啊人际关系啊什么的。

R：那你刚刚开始碰到孩子的事情受挫的时候呢？

P：所有我所受挫的这个事情当中，孩子这件事情是最最不伤心的，真是这样子的。我跟你说我所以我最伤心的事情都是20多岁的时候，比如说到二十八九岁的时候，也完全我好像比同龄人就感觉晚成熟五年的样子。然后到二十八九岁哪怕就到了三十二三岁我还是有很多工作上比如说人际关系，因为老是争强好胜嘛对吧？然后你争强好胜的人，有时候你尺度把握的不好的话很容易遭人记恨的，你这个人就关系你情商又不是很那个的话是处不好的对不对？所以呢在很多在这方面是包括工作这方面是有困惑和比较折磨人的这个部分的。我认为生孩子这件事情就是那不是很简单，只要你想要那不是就还能没有对吧？是这么想的，后来才遇到这些事情，慢慢再加上当时的话工作上也觉得可能也走到一个瓶颈了，为什么？就觉得可能如果我拼尽全力，我也只能做个这个中上等我成不了最优等的那批。就是这件事情给我的打击还是有点大的，因为我一直以来一直好拼，对，就是想在成绩这方面做到我能想到的高度，但问题就是说你想的跟你的能力不匹配的时候是吧，你必然会产生痛苦，你这个痛苦不和解，你在心里，那你如果一定要套上去的话，必然肝郁，肝郁必然气血不好，气血不好必然怀不上是不是？这都是有生理基础的嘛对不对？对，然后，所以但是当时并没有把生孩子事情当回事情，后来也是说算了已经到这个位置

老公事业心更强了，而且到这个年纪了我停一停，我要去生孩子啊怎么怎么样？那么生孩子这件事情我是辞职去那个的，因为我没想到这么挫折，我从34岁的后下半年辞职，你看今年虚岁39岁了，这么多年都没有上班。所以你说这几年当中你说自己内心能没有触动吗？因为对我来讲本来我是很在乎事业也好，或者存在感也好，价值感也好，突然之间这些东西都没有了，然后突然之间你要去解决这个孩子的事情，然后孩子的话其实前几年我也并没有做好，就说我能做一个好妈妈吗？这种心理准备我没有的，但是你现在问我的话，其实我觉得我好像有这种心理准备，因为我已经想明白我很多困惑东西我都想明白了，然后也不是说现在不上进，我依然我是上进，但是我不会说太执着于自己达不到的那种，而且我觉得我心里会更踏实一些，也能够更坦然一些，不会说特别的急功近利，或者说特别的急躁，就是爱别人要那样，我也那样，不，我现在有自己的这个想法，然后我也有自己的节奏，就是我也不在乎别人的眼光，反正就很坦然，对。

R：给我的感觉就是你现在是大彻大悟了的那种，但是我觉得通常情况下在大彻大悟这件事情之前通常都是很难过来的感觉

P：当然难过咯，但是我说的难过的话，我就说是——

R：不是这件事情引起的吗？

P：对，工作，主要是工作引起的，真的是工作，对，所以我跟你说我们说的不好听一点就是我跟我老公都有一点不懂人事你知道吧，不懂人事是什么，因为我们的生活背景是什么，两个人都是老家是农村的嘛，都是通过考学，然后一步一步然后去做到自己那个什么的，然后所谓比如说情商教育也好，什么教育也好，其实并没有，至少或者说我们家里人都是比较这种正直啊什么，就是靠自己的能力啊怎么样，最讨厌那种弄虚作假那一套，就是所以呢两个人骨子里都是那种相信，就说我要靠自己的力量要怎么样，不像有的人成熟得比较早，就觉得生活不就这样吗，结婚什么生孩子的那种家庭怎么样，我们是正儿八经的那种有点像某个根正苗红的年代那种很单纯的这种想法，然后直到就是说30多岁的时候，那会你知道我老公怎么讲，就说我说生孩子的话怎么办？我们都没有时间带，她说把孩子送回老家让父母带不就行了吗，然后或者是怎么样让父母带着小孩，然后住一套房子，然后我们住另外一套房子怎么样，我当时我就觉得你说他怎么会那么幼稚，因为你不可能你显然是不可能的，但是这就是我们当时的这个状态，但是我当时是觉得大事上我还是挺放心我老公的，他的很多决策我大事上都是非常不错的。所以我刚好是赶上了一个为什么说这个孩子没让我很伤心，就是因为我们从小的这个生活背景并没有说被家里教育成，就说你工作以后赶紧结婚，结婚后就赶紧生孩子，你这一辈子怎么样并没有，然后我父母他父母都是觉得你们要好好工作，

而且我父母他父母从来不会对我们做的选择做任何干涉，都是支持，你们说要干嘛，我就支持。我们父母就说我这一点绝对是比较幸运的，就是说他们不一定说能给你帮上什么忙，但是他不干涉，然后你只要说你想这样做那么我就支持你，都是这样子的，所以呢我们两个人就不太懂人世间这套东西，直到就是年纪大了以后，然后我慢慢开悟了，那么刚好开悟的时候遇到了这件事情受挫了，而且再加上受的心里最磨难的那部分，我觉得我从25岁开始吧，就25岁到35岁之间属于就是说特别的很懵懂，然后呢不停的自己在跟自己内耗的这么一个过程，最痛苦的是那段时间，跟孩子就是说关系还不大，就痛苦的，其实一个表面上看起来是工作，实际上就是说你怎么样跟自我与这个世界的关系，对吧？因为你想就是说以前你的眼里只有学习，对吧？只有什么这工作，但是那会你想你之前学习的时候都很单纯的无所谓的，你这个人关系好不好什么的，或者说压根没有也不是应该不好，压根不会去有意识的去建立那种人际关系，但是你一旦工作以后你就会发现人家那些人比如说有利可图也好，或者是怎么也好，都是这种平时以前自己完全看不过眼的这些行为，反而是受欢迎的，就是自己原来原生家庭成长起来的这种价值观跟是非的观念跟现在这个完全是两样的，所以就是说出整个我觉得十年都纠结在这个痛苦当中，然后但是自己又不想就是说，因为你已经习惯了，好像一直看起来是挺优秀的样子，对吧？在这个环境当中水土不服，但是我又不承认说他这个就是对的，这个就是好的，然后但我又不承认说我就是落后的，我这个就是错的，他一直在这样内耗，真正就是说后面你就想开了，然后就说孩子这件事情，你现在问我就是说，如果让我有个选择，就是你什么拼工作，还是说拼孩子，我可能觉得还是会拼工作的，就是可能我就这种性格的人，即便有了孩子以后，我觉得我不可能说把所有的这种精力都放在孩子身上，就是说好像我就全职的完全啥都不干了。是的，我好像是能够觉得有的人有了孩子以后，可能我当然现在我没有啊，但我想象我感觉我应该不是说有了孩子以后就全绕着一个孩子转那个人，不是的，我还是会说给他独立的这种空间，放手啊或者怎么样子，我也得忙我自己的，对，所以我觉得你觉得那些人，因为孩子这件事情特别伤心或者怎么样，可能他们家庭观念本来就很重。

R： 对，然后还有一点就是他们可能跟你有一点不一样的是，多多少少他们会有来自一些社会家庭这种压力，

P：那我没有，就包括试管婴儿件事情，很多人她都不愿意跟人讲的，那我身边的朋友都知道，然后我也没有什么好隐瞒的，我觉得无所谓的事情。对，我觉得我比较坦荡，好像我也并没有说因为做这件事情我就低人一等，并没有。

R：事实上也确实没有

P：对，但很多人会介意，对，是这样。

R：那你觉得这个事情有没有影响到比如说你的一些社交，P：那没用，为什么会影响到？别人会歧视你或者怎么的？

R：不，不是说歧视，可能两方面，一个有些人可能觉得一个我可能觉得我好像没生出孩子挺没面子，另外一个还有一个孩子也是一个话题嘛对吧？

P：那并没有，我像我关系走得比较好的，这都是认识 了十来年20来年或者说七八年这种朋友都是关系很稳固的，我们像包括他们有孩子的，我们绝大多数的时间也不是聊孩子，我们都是聊各自内心的一些成长啊收入啊，最近有一些什么好的感受啊，或者说比如说单位的事聊一聊，两个人就是开解开解啊同仇敌忾一下或者怎么样，就我们那个我说的这个女生的话，她的成长经历跟我也是差不多的，但是她比我小时候就受到的挫折更多，所以她会成熟的早一点，然后他们的话也是要的你看现在很多人要孩子二十二三二十几就开始要了，我们那会在上学想都不会想，对，刚毕业的时候更加也不会去想说，比如说那个了就是说结婚我都二十七八，我虽然是26岁领的证，但是我办婚礼到了30岁我才办的婚礼，因为那个之前我都觉得我就觉得好像我还是个小孩，然后在那种觥筹交错之间好像又递烟又干吗，好像那种事情，我就觉得就不应该是我做的就那种，对，所以就是还是说性格不同。是这样的。

R：那可能你确实感触不深

P：但是我觉得你写这个东西的话，其实也未必说一定要写这个有多悲惨，实际上还是跟他的内心需求有关系的嘛。

R：对对对，跟每个人内心需求不一样，我也不一定说一定要把他觉得所有人都觉得很难过特别什么，但是可能是因为在我们自己科室总归是这种需求的会多一点，对，像你这样看开的倒不是很多，但是肯定也会有。

P：肯定有的，对每个人性格不一样吧，对，是这样。

R：到底是哪一个点让你就这么个大彻大悟了？就这么想开了！因为有很多人都走不出这个影子

P：我并不能理解他们为什么走不出。我之前看新闻的时候说周润发的老婆是吧？她不是是他们之前怀过一个孩子，好像也是几个月没了，还花了七年才走出来，然后我就特别不能理解为什么这件事情要花七年才能走出来，我是这样想的，R：他可能是个个例七年时间，但是我觉得大部分人可能都觉得这是一次这是相当大的一个打击，或者说是挺难以接受的

P：我觉得可能还是我月份小呀，你想我才比如说移植完以后移植完才开心了40天，然后就没了，

R：但是你移进去怀孕也不容易的呀

P：没有，很容易。对，我跟你说，所以我就是说我是基本上一旦放进去它肯定能成就这样子，我没有试纸之前我就知道肯定有的，因为我有感觉，对，然后但是就是说我刚开始

第一次确实是不知道为什么不知道还有胎停这个事情，就是功课也没做好就没当回事情，然后再加上实际上我备孕的那几年也我现在是没有在上班了，但其实也在忙别的事情，之前就是也带了带着一个微商团队（不禁笑出声），忙得不可开交，根本也没有太多的想法说什么，对是这样子，后来就是因为年纪越来越大，因为我给自己主要是定了一个时间是40岁，你想你35岁跟40岁还五年，但是像这次移植之前，本来呢18年的11月份是要想移一次，然后结果那会内膜就没长好，然后中医也是劝我。你看我是最后一次胎停是17年的2月份，然后整个17年到18年完全没有移植，一直在调理，每次调理就说行不行吧不行再调理半年，不行再调理半年又半年，我最后我都真的是特别的躁，我觉得这件事情什么时候是个头啊，我现在最后就变成了那种我不是那种就是说人家什么头然后就哭，我就什么时候是个头，然后结果就是要么我现在只想要一个结果，要么有要么没有你给我哪个都可以，只要这个事情画上句号就行了，我就这么想。至少我就觉得我不要把我的生命都耗在这件事情上，因为我还是觉得就是说人的生命不是说只有孩子才是唯一选择，其实孩子你也看到了你自己，包括你自己长大以后你会说把你的父母当成非常重要，你其实还是会忙你自己事情对不对？这样的，所以我的孩子将来也是这样子的，无非就是我要陪着他走一段路，但是你说而且我在这边在你们这边医院看牙，然后你们医院看牙，有一个牙科的医生，他也说哎呀你不要着急，他说你这个东西要有，老天要给你一个缘分的，没有给你缘分，你急也急不来的。而且我也不瞒你说，我也是看过大师的。其实我觉得这件事情确实也是给了我一些，是释然了很多，我具体内容就不讲了。

R：你指的是什么事情？怀孕这件事情吗

P：对。那个大师他就给我解释了一下，意思就是说给我解释出来之后，意思就是说之前没有或者什么的这些我就释然了，其实这件事情也给我一点就是很大的一个心理安慰的一个部分吧，这样子的。因为像我们这样的多年不孕的，不管他们别人有没有跟你讲，绝对都是要去拜一拜，或者求一求问一问，有的一定有的，对，所以呢总之就是说，而且关键就是说我自己就相信缘分这件事情，我不是跟你说我都要想去皈依吗？就是在家修行或者什么这种是吧？所以总的来说就是说已经明白了这个世事，就是世事是无常的，是吧？就是叫什么月有阴晴圆缺，人有悲欢离合，本来就是无常的，你就何必一定要去纠结了，而且就是也不要把那个事情想得太完美，包括我的表弟媳妇老是很纠结，哎呀我到底要不要生二胎，你看我自己，她是独生子女嘛，我自己的话从小到大就感觉好像小时候挺幸福的，没有人跟我抢东西，但长大了以后现在好有压力，就是没有人跟我商量，然后她就觉得她的小孩会不会很孤单，后来我也跟他讲，你也不用说老纠结你自己的事了，首先你妈是不可能再给你生的，其次是也不要把这个事情讲的太美好，你这个兄弟姐妹有缘分的固然是好的，也有很多没有缘分大打出手太多了是不是？所以包括孩子这件事情也一样的，也不要把它想的太过于完美。

R：所以和你们聊聊我也是收获很大，每个人的内心都不一样，有时候你们想的比我透彻。

P：主要是我们这个都年纪也大了，然后天天也经历过很多磨人的事情，你不想开点怎么办？不想开一点，天天——我们我跟你讲我那个中医，而且就是我也觉得挺那个什么。确实想看以后这个心情好了很多，然后呢有之前想不开的时候，我想不开是说在执着于这件事情我为什么没有成功，就怎么讲呢，因为呢总感觉我拼了这么多努力来总该有收获了，是这个意思。然后后来我们那个中医就说你要每天就是要开心两个小时，我这就当药方的就是说要怎么想开或者怎么样，所以你就得想办法去（开怀大笑）。

R：很有哲学

P：是我后来想我其实更适合做心理学方面的，然后——

R：包括你把这个事情看得这么开，我觉得其实像你这种想法跟像你这样的有这样这种经历的人，跟你类似这种经历的人多交流一下，我相信他们多多少少会得到一些一些启发。

P：但是绝大多数人不会听的，因为绝大多数人他们认为一个完整的家庭，没有孩子的话不完整什么，没有当母亲心里依然有这种传统的观念，其实我这种想法我之前也不是说完全没有，但是我不是说特别的坚持，就说我一定要有一个怎样，我只是慌，我只是说假设没有的话，我只是会慌。

R：慌什么呢？

P：就是比如说我能不能扛得住，比如说我现在不后悔，那么将来我到了四五十岁的时候，我会不会后悔，我这一点上我是稍微有一点慌，但是我就前段时间去了灵隐寺以后（放声大笑）

R：又是大师的结果

P：没有没有没有，我那天回来真的感悟很多，然后我突然觉得好像我也不怕了，因为什么就是觉得其实人啊再说的直白点都会死，是不是？你在这个世界上再加上我也经常读心经呀或者什么的，其实都是真的都是皆空的诸法都空巷的，所以你何必于纠结于外部这些事情呢，就干脆一点选定一条路，因为什么任何就想白了一点，任何一条路都是有好的必然有坏的。那么你虽然表面上你看别人都有孩子或者什么，你想象中觉得很好未必如此，你选择这条路也有他们所不能及的好处，而且就是我的朋友也会跟我讲他不是自己本来他们小孩已经十一二岁了已经上小学了，他自己意外有了一个二胎，然后他是很想生下来的，然后她老公又不想生下来，后来他还是生下来，生下来以后她老公觉得就干扰了他们正常的生活节奏，本来老大已经是基本上不用怎么太那个了嘛，他们后面的生活就会很比较舒服，结果现在又有一个相当于路要重新走一遍就埋怨他，所以他自己现在又工作又带小孩压力挺大的，对，所以他也跟我说，他说你也不要压力那么大，跟你说有孩子还有的好处没孩子也有没孩子好处，他之前老跟我这么说，我其实也并没有说听到心里去，因为我还没有下定决心说，如果说我不敢保证说我就是没有了，我以后不会后悔，然后但是呢我跟你说，后来最近我觉得应该说上个月春节过后吧，然后就觉得其实确实是这样的道理，什么都有它的好和不好，你也不要就是说看着人家，只是看着人家的好，然后你呃你也要想想，人家的不好，你自己你愿意经历吗，对吧？所以想到这些东西，其实也就反正我没那么纠结，至少就是说我不去想说我会不会后悔，我就觉得你既然选一条路就不要后悔。我就这么想，就这样子，但是究竟到时候会不会我不知道，但是至少就选那条路就坚定的走下去，因为我也不想再回头，没意思了，是不是？反反复复反反复复，这个事情是没有头的。你说哪怕如果我反反复复，或者说我还一直很执着，我要一直生到为止生为止，我假设45岁我生了一个孩子，你有精力带吗？你后半生还有你的生活吗？这个孩子如果说带的不好的话，我觉得我的心里反而觉得更失败。是不是？对我来讲我需要一种成就感，我可能我无法接受那种只是肉体上感觉你活得很好，但是你的精神上活的不舒服，我不能接受这样的生活。我可以说物质上我没有太多什么很浮华或者什么很那个的奢求，但是我精神上我一定要是非常的这种清明的，非常的舒服的。然后自在，我需要这种感觉。就这样想的。（停顿了一会儿）还有什么问题？

R：呃也没什么了，我觉得。基本上也都是说了你的感受了，关于流产的这件事情有没有什么想补充的？移植流产的这件事情有没有什么想补充的？

P：移植流产啊，移植流产嘛反正也——（停顿）

R：或者说你当时刚发现流产，或者说流产过后，当时那个点有没有特别让你印象深刻的？

P：好像也就挺麻木的，就那种。因为我听到第一次听到的时候是很震惊是吧，反正后来嘛又跑了好几家医院，他也确实说发现说胎停啊怎么样？也没办法，就感觉那个算了是这样子的，然后后来第二次的话就又胎停了，当时肯定眼泪也肯定要掉眼泪的嘛，但是后面的话好像有点——呃并没有说天天哭，没有，好像就那两三天很不舒服，然后后面做也就做了，再后面好像有点麻木反正并没有很伤心说，没有，也没有并没有说我又怕胎停，我不敢去移植或怎么样，我只是觉得好像有点像越挫越勇那样子，对，我就觉得我前面为什么这样胎停，我就想去找原因，然后呢所有的原因都找了，像第一次的话，他胎停我去上海一妇婴查了所有的免疫的因素都好的，然后第二次胎停，然后我又查了胚胎所有的染色体的也全部都好的，所以我其实最主要的事情都是在做那个找原因，然后后来原因都找过了嘛因为医生说起来就是说难免流产嘛什么的说明这个胚胎有什么优胜劣汰啊什么的，你反过来想想，如果说真的是一个有问题的胚胎，你生下来更麻烦，是吧？最后反正事情过了没嘛也就算了。而且我是有点心理上有点不会记那么久的，就是有些事情就是说不会——

R：不会钻牛角尖？

P：也会，有很多事情也会，但这件事情上我不会因为这事，而且最主要的是什么？最主要是我觉得在我当时所知和所那个的能力范围之内，我尽力了。我觉得能是否尽力这件事情，是让我能否放下这个的一个重要的因素，如果说我没尽力，那我肯定是很懊恼。但是我也不是说因为纠结于这个东西的失去而懊恼，而是因为我自己没有做到而懊恼。可能还是说跟个人的性格有关系吧。

R：挺好的，觉得我今天收获还是挺大的

P：真的我以后生了孩子的话，如果有机会的话，我可以真的告诉你我生了孩子以后具体是什么感觉，因为现在没生的话，你站着说话不腰疼对吧？你没有切身的感受，所以我经常我也问我朋友，就是你说你生这个孩子以后，你是不是真的觉得这孩子你的所有就是你的全部，然后这两个朋友都说那倒没有，但是就觉得如果说离婚什么的话，绝对我不会把这个孩子给我老公，我一定要我什么都不可以不要，但我一定会要孩子。还是这么说的，但是,我没有任何感觉，对,我没有任何感觉，所以就可能就是说还是要真的是经历，经历到那些之后，才会——可能那会的想法可能又不一样。到时候如果有机会的话，会肯定不需要这个课题了，你都做完了。

R：没关系，到时候可以再聊

P：是吧，所以应该顺利的话应该是好吧。

R：行吧，都快9点半了，不耽误您休息。
