# Supplementary material for: An insurmountable obstacle: Experiences of Chinese women undergoing in vitro fertilization
Source: PLoS One. 2024 Oct 7;19(10):e0311660. doi: 10.1371/journal.pone.0311660 (PMC11458033; doi:10.1371/journal.pone.0311660)
Supplement: S1 Data — (ZIP) [file pone.0311660.s001.zip › data/P9.docx]

R:能不能跟我说说你在生孩子这件事情上一路走来的感受什么的？主要侧重于自己的心理方面。

P:这个说一下马上要流泪的（哽咽，一下子眼眶就湿润了，我递上了纸巾，等着患者情绪缓和下来）。就觉得很难嘛，因为——我是14年结婚，差不多一开始就促排，促排后面不是输卵管不好么又试管。那反正就说给我感觉就是——就是辛苦吧，可能最怕的一步就是取卵。

R：不麻药吗？

P：邵逸夫有局麻，但是跟不打麻药没什么区别。

R：就是怕痛吗？还有别的因素吗？

P：也不是怕痛吧，因为这个刺激太大。就是第一个他不是取卵之前打的药促卵泡嘛，那个药就是副作用很大。然后取完以后那段时间漏尿啊。。。就这些，生个孩子难嘛。

R：那么有没有让你印象特别深刻的事件的？

P：大概就是促排取卵，然后或者是移植前那段时间吧。就一直增内膜啊什么的。

R：这些都是治疗的辛苦。

P：对。

R：心里辛苦呢？

P：心里的话——怎么说呢，心里其实还好，像我的话怎么说呢，我感觉我生孩子压力主要还是来自于父母。其实让我，如果我自己——我和我老公两个人其实无所谓对，主要是父母——父母催得紧，压力比较大。然后另外其实倒没有什么。

R：别的方面压力？

P：别的方面倒没有。

R：都没有。你之前是胎停过的是吧？

P：对。

R：那胎停胎停以后有什么感触？

P：开心

R：啊？

P：我那天邵逸夫，然后那时候二超，二超以后，医生说胎停要约流产，哇——那个心里那个兴奋，一下子冲到501赶紧去买了跟甜筒。

R：胎停为什么开心？

P：也不是开心吧，就是一块石头落地了。因为我在家里就怀孕的那段时间，在家里，我老公就和我妈什么都不让我坐，然后什么都不让吃。然后给我的压力就是我自己还没有给自己压力，他们已经给我很多压力，所以我整个人就那时候心情也不好，整个人很抑郁的，然后医生跟我说，就是小孩子心跳停了嘛，然后其实反而——也不是说开心吧，就是感觉那块石头落地了，然后就赶紧去吃了个甜筒。

R：那之前是什么样的一个心情呢？

P：之前其实开心也开心，但是我觉得家里人给的压力太大了，可能第一次怀孕他们也紧张，他们给我的压力太大了，

R：就是觉得压力大。

P：对。

R：那为什么后面还要做？既然胎停这么开心，为什么后面还要去做移植？

P：那我自己也会想要小孩子啊，只不过说没有说像我父母他们，就可能在农村里面别人会议论什么的，所以他们特别想要个孙子什么的。所以就这样，我其实还好。

R：因为我觉得一般去移植的肯定也都是承受了蛮大的压力什么，然后自己应该期望值应该也蛮高的，想要的。

P：对啊，你像我这段时间住在这儿，虽然说没开奖就没开奖的过程，就是我老公就说哎呀你盘腿也不能盘，头发也不能老是洗，然后干嘛干嘛，像我这样有洁癖的，我每天都要洗澡洗头的人，然后就变成三四天洗一次（笑），然后整个人就不舒服。

R：那么现在心情是怎么样？

P：现在，失望吧。然后就在想会不会是移植医生出问题了。因为我觉得我移植着床应该是没什么问题，可能是移植医生的缘故。因为我自己也感觉这次医生好像移的过于快了。

R：那之前胎停以后除了开心，还有别的想法吗？除了那块石头落地了，有别的情绪想法这种吗？

P：后面就是开始调理嘛。

R：前面知道这个消息以后，你的心理过程说什么样对？

P：那时候其实你说开心，其实也不是开心，就是放松了。家人和老公给我的压力就就是放松了，我其实那时候我自己没有给我自己多少压力，然后流产了，肯定自己也不会多开心。但是我第一次怀孕，我自己也没有什么特别大的感觉，因为也没有孕吐，也没有各种反应，所以我就好像没有特别大的那种感觉。

R：：那当时怀孕的状态有没有说自己特别紧张这个孩子什么的？

P：：那有啊，因为我——邵逸夫她保胎不是很好，移植好保胎不好。所以我那时候像隔天查HCG都是我听别人说和我自己查来的，然后才知道要隔天查。等到我去查这个指标的时候已经不行了，然后就等于说翻不上去嘛，翻不上去我才到市中来看。然后医生那时候跟我说，等于说已经翻得太慢了等于说住院也是没太大必要，然后就一个星期我就胎停了。

R：这一个星期有什么特别的感受吗？

P：这个星期啊——焦虑。

R：因为什么原因引起你焦虑呢？

P：一方面嘛其实我也想这次成功，因为毕竟旁边住的都是孕妇，也有一种想当妈妈的感觉。然后另外一方面就是最后两颗胚胎移掉了，后面如果不成功的话，就等于说还要重新取，想想取卵也是蛮痛苦的。

R：那后来是怎么样调整过来的呢？

P：也没调整吧，我觉得还需要回家再调整一下，

R：心情是怎么调整的？就是从你胎停到后面不是又重新去移植嘛

P：我那时候花了一年啊，我到一年以后才去移植。

R：那这一年都有什么改变吗？

P：就跑健身房啊，然后——

R：除了行动上的改变，心理上有什么变化吗？

P：心理上也就——好像没什么变化，就该吃吃，该喝喝，该睡睡，呵呵——

r：也挺大大咧咧的我看你。

P：其实我还好，我自己还好，就是很多我基本上生孩子啊或者干嘛的压力，全部都是我老公和我爸妈给我的。

R：他们的压力相当于给你了其实也就到你身上来了，慢慢的也就变成你自己的压力了。

P：对，就我不是很喜欢被别人管的一个人。然后他们每次在什么时期的时候不停地让你不准这个不准那个，虽然说知道为你好，但是我这个人比较叛逆，就是很不喜欢这样子，自然而然心情就不好，心情不好就影响到自己。（停了一下）

R：当时怀孕也好，胎停也好，就是心里有没有一些担忧方面的具体一点的想法。

P：怀孕的时候担忧嘛就是想着毕竟做试管才第一步，成功了么，成功了你想想后面还有保胎，还有NT还有三维四维还有唐筛。想想都是卡。想想都可怕。是吧？胎停了以后，反正就是失望最大吧，但是那段时间还是蛮快调整过来了。

R：为什么呢？

P：可能——可能那一段时间我朋友住我家，然后每天我就跟她在一起，因为我去医院也是她陪，她和我老公都陪我的。所以反正胎停，就她说胎停就胎停了嘛？胎停就火锅啊冰淇淋啊走啊吃啊（轻松地笑）。所以就这样好很多。也没去多想，想想看还有两颗（冻胚）大不了再试一次。

R：当时是还有两颗的。

P：嗯，现在就等于说没有了，要重新来过了嘛，所以这次可能压力更大一点是吗？

R：压力——对，压力肯定有压力，因为我还是比较怕取卵的。不过听说今年有全麻。

R：是有全麻，之前我也是听那个谁也在说现在有全麻了。

P：可是全麻我都不敢做，因为我麻药打太多了，怕脑子打坏了（笑）。

R：那家里人呢？你说你家里人也挺给你的压力挺大，那这件事情对他们造成的影响具体有哪些能跟我说说吗？

P：像我妈——反正，基本上每天都要问一下，怎么样啊，然后一到抽血的日子，“记得给我打电话哦”。对，她一方面也舍不得我做试管。因为做试管对女人的确来说是太伤了。

因为我有嫂子，我嫂子做了四次试管，取了三次卵。然后她现在小孩子上一年级。就整个人看起来就是很老，眼皮下垂啊反正就是各种腰痛啊，腿痛啊，就各种后面的那种后遗症都出来了。所以我妈也很担心，我后面会不会也这样子。

R：那时候除了跟闺蜜经常在聊聊以外，就胎停以后的调整了，然后心理方面还有没有别的方式的调整？就自己调整以后的想法是怎样的？

P：自己调整以后的想法——那时候其实后面我本身也没有太过追究说孩子胎停干嘛，我后面就是找原因嘛，因为我那时候不是查出来，APS阳性，我就开始找APS阳性有哪些方面，然后就开始看专家，然后检查各种全套的免疫啊什么的。查出来医生说没什么关系，没有太大问题。然后所以就等，身体也觉得差不多了，然后觉得各方面什么可能会导致之前胎停的因素，那我把它排除掉了，我才去做第二次移植。

R：感觉就是自己去找原因，找问题去解决问题。

P：对。

R：别的想法有吗？

P：别的想法好像没有。

R：家里人就是在这件事情上前后有没有什么变化什么的？

P：没有，因为我妈也知道我这人脾气还蛮大大咧咧的，我调整好了她其实看看也看得出来。

R：老公方面呢？

P：我老公其实还好，他其实大不了生不出么就家里养养狗啊，就两个人也挺好的。

R：也是这种想法，那你也是这种想法？

P：最好么是有个孩子，是吧？我们俩其实夫妻感情好的，就是能努力一下生个孩子么生个孩子。

R：那老公对你在这件事情上说怎么影响你的？

P：这次我看得出来他蛮失望的，比上一次好像要失望。

R：那上一次呢？怎么影响你就是比如说类似于比如说有些鼓励或者这一类的，它是怎么影响你的？

P：他就心疼我嘛，就要打这么多针啊取卵干嘛的。本来我老公在家里也蛮体贴的，干家务烧饭啊都会。

R：那胎停后那段时间她是怎么影响你的，或者说他是怎么一个状态？都做了哪些事情？

P：因为他看我也没啥事儿，他也没有来说说来安慰我啊干嘛的，也没有什么特别的那个，因为我自己已经调整过了。

R：周围的人际关系呢？

P：周围的人际关系——工作的话是我开始要孩子开始我就辞职了，因为老是要跑医院嘛，你去单位也不方便。是吧？所以我就专心生完孩子再上班吧。

R：那压力会不会更大一点？

P：那还好，其实压力不会大。主要就是打针啊促排啊这些方面比较有压力。金钱方面无所谓。

R：你觉得比如这次又失败了，你觉得有没有哪些方法可以让你重新树立信心这种？

P：那——今天回家肯定先去吃顿火锅，然后冰可乐来一罐。

R：除了这方面别的方面有吗？

P：我是打算下午回家先去找我闺蜜，我也不想呆在家里，因为我觉得我一回家面对我妈我就不舒服，所以我觉得一回家放下东西，我就赶紧去找我闺蜜去。

R：为什么呢？

P：呃——怎么说吧，像我闺蜜她们其实还蛮懂我的，就是我做了移植以后他们不会来主动找我，除非我去找他们，他们不会来主动找我，也不会说问你怎么样或者干嘛，就等我去跟他们说。就这样子。像家里人就是那种关心则乱的感觉。是吧？一个劲的问你，反而给你压力。

R：就需要理解支持。

P：我觉得跟我朋友在一起可能会好一点。就跟一个人或者跟我爸妈在一起的话，就可能会想的比较多（哽咽）。

R：那老公呢？

P：也不要。

R：老公也会给你压力

P：也不是，他自己现在心情也不好，她可能心情还没我好。他也不是特别会表达出来的那种。

R：那你自己这方面还有别的一些补充吗？有没有害怕什么东西？

P：害怕什么东西？好像没有吧，我觉得如果这辈子生不出孩子么就生不出吧。我觉得反正这么多人现在都不结婚，不要孩子，是吧？也没什么多大关系。就是家里皇位没人继承了呵呵呵——

R：你挺可爱的。

P：我妈那时候还说，别人还建议我们家去领养一个。那我那时候还在想，我才不要领养，一点血脉关系都没有，万一养个白眼狼出来怎么办？

R：那么——有没有什么感悟的？

P：感悟啊，我觉得下次来保胎的话，还是确认官方以后再来。

R：为什么？

P：因为住在医院里，旁边都是孕妇啊，自己会很期待这次赶紧受孕成功，然后待个三个月再回家。然后你一下子知道自己落空了，就是那种空虚感很强。就在这里会比较焦虑。其实我在家里的时候，上一次的话也就验孕棒测了也就有了，也就去验血了。也没有说特别大的那种焦虑感。但在医院里可能都是孕妇，你就是焦虑感特别足。上一次没有住院，那时候不知道大家那么多，移植好以后会跑到中医院来保胎，而且不知道保胎有这么早，就是后面她停了以后我去问了，问了很多做了试管的人，他们都建议官方以后马上过来市中医院保胎。这样子孩子比较容易保得住。对。然后所以这次就过来了，但是我觉得我住的太早了。心情紧张，对，这样落差反而大。你可能待在家里，该吃吃该喝该睡睡该玩玩，其实也没有多大影响。毕竟医院里也无聊。

R：对，每天就等这么一个结果。躺着等就是。

P：对，就打针方便一点。

R：那关于移植以后的病友群体，这方面有没有什么感想吗？

P：怎么说呢？因为我们群里的话很多都是基本上不是一次成功，基本上试了很多年的，都有好几次，也觉得跟别人比吧，我也不是一次成功的那种幸运者。反正继续试吧？再试一次看一下，我觉得自己还能坚持，就再试一次。说坚持不了就算了。

R：我该问的都问完了。你还有什么要告诉我的吗？

P：那没有了。
