# Supplementary material for: An insurmountable obstacle: Experiences of Chinese women undergoing in vitro fertilization
Source: PLoS One. 2024 Oct 7;19(10):e0311660. doi: 10.1371/journal.pone.0311660 (PMC11458033; doi:10.1371/journal.pone.0311660)
Supplement: S1 Data — (ZIP) [file pone.0311660.s001.zip › data/P5.docx]

R：你觉得这次的事件给你造成了哪些影响？

P：影响是很大的，首先因为我之前有移植失败的，直接就是第十天官方就失败了，那么虽然遗憾，但也没有什么，但这次希望很大的嘛，反正也都正常的长，也没说什么不好，突然之间就没有心跳了，然后我就是一开始想不通，一开始觉得不是真的是假的，然后后来不是又去做了个B超，那么是真的嘛，那就。。。就也只能这样接受了，勉强的接受了。然后就是会想很多，为什么会这样子，然后想了很多，其中有几点原因，因为我都也没跟别人说过。第一点，因为我当时问刑医生，他就给我看了一下，因为当时失败了，作为医生他也没说什么，他就说胚胎质量问题可能大一点。那么可能这个是一个原因，但是我觉得这个原因就是最模糊的，最不知道到底是不是这个原因是吧？但其次还有一点，我妈老是那个心理就跟我爸有点耿耿于怀，就是楼上不是那天装修，装修油漆味很浓，我那个房间特别浓，然后我们就走出来了，并且没地方去，我们后来还去了9号楼一楼地方坐着，那天我正好也是回家了，然后我妈就是说可能就是油漆味蒙了蒙，然后就是说回家我要开一个多小时车子嘛，颠波了一下，然后，因为老人嘛，他心里会有点那个的，就不大舒服。毕竟我到你这里来保胎的，然后因为你这个事情而导致我要回家这么路远，他肯定，就算不是，有其他原因，他想就可能这个原因也占一点点，老人他不懂科学的，他一点科学不懂，他不懂油漆味其实闻一天也不会说是停胎的最多畸形，我常识还是有的，但是他作为他来说，我好好的在这里保胎，我为了这个要回去，然后颠簸了一下，然后我爸甚至说要么再去说一下，因为当时，当时我问这里的王护士长，她说她说我去看过了，她说楼上在装修根本停不下来她说，我这个时候我觉得没有办法是我打的，12345，我打了12345以后，可能那边电话打过来，可能就是这边就停掉了，可当时是没法停的这里。然后因为他也不跟我们说一声，就是要油漆了，如果说一声我就那天就打完针，我们早上8点钟打完针我就回家，然后等一下没了再回来嘛。我们也理解，我不是说——我是到这里来保胎的，也不是说来找事情的，然后嘛后来第二天十楼的护士长来找我了，说叫我给他撤掉什么的，那我也给他撤掉了，我想想也没有，反正这个事情是一个插曲，正好那天弄了以后抽血还是好的，HCG涨到了十万八，那天就是大家还蛮高兴的，就是说因为也还可以蛮理想的，涨的倍数。好，隔了一天就是做B超嘛，之前医生查房的，说你这次B超好的话就是可以回家了，家里养，那我心里也挺高兴的，做B超之前还挺高兴的去做。然后做着做着他就说——他就说没有心跳，那么我也不相信，然后他叫别人来看，没心跳嘛我觉得天都快塌下来了。（苦笑）

R：觉得天都快塌下来了。。。

P：嗯，那是的，多少难受啦，然后当时在那边缓不过来，

一直坐在那里，也没有回去，坐在做B超的外面。

R：当时想的最多的是什么？

P：想得最多的是怎么办？怎么能救救他，就是能把它弄活一下，然后就怎么办，我，我爸我妈都在嘛，然后三个人商量，这怎么办？然后怎么回事情怎么办？要不再去其他医院看看，会不会误诊啊什么的。后来坐了大概有半个多小时，后来我就回来，回到这里了，问医生了嘛。问那刑医生，我说我心。。。B超做过来了。。。我说好像心跳没了。他说“啊？”他说他也很吃惊嘛，他很意外，他说，没心跳了？然后他就给我看了，他说，看了一下，给我——他也试图找原因嘛就是，给我从头到脚这个病历翻了一下，他说：“你之前是有一两次HCG涨的并不很理想，但是涨么也在涨。后来是涨得还好的，后来就是说，第一次做B超嘛他说什么疑似心跳，说明心跳不是很厉害。他说可能这个有点不大舒服，一般它出来心跳也蛮强的，他说。然后也说不出什么其他也没说什么。可能胚胎质量。那我妈不是还一直问他：翻倍好好的怎么会这样，翻倍好好的怎么会这样？他说那人家好好的到35周不是也胎心没有了。

R：我们看过是蛮多，但是对你们而言心里确实有这个疑虑的。

P：对，是的。

R：你自己是怎么想的？

P：我——呃现在，当时的想法就是说呃——已经接受事实了，就是——怎么办？然后就问了一下那个——哦当时去邵逸夫做了一下，然后回来以后问了一下，吴医生在嘛，问了一下怎么办？怎么弄，去怎么弄，要保身体的事要怎么弄嘛，就是说他要去掉胚胎什么的。那么就咨询这些东西，后来马上就咨询这些东西了。然后现在包括吃那个药嘛（药流），其他的没想，就往这方面想了，怎么样把它顺利拿拿掉，那也没办法啊，他都没法救——然后就是让自己受到少一点的，我说我不想做清宫，然后他就说帮我挂盐水。今天我出血还是有点厉害的，不然不会挂盐水的。其它还有什么？

R：最深刻的就感觉最深刻的是什么呢？对你而言，或者说你觉得就是说以后怎么办？为什么觉得是天塌下来了？

P：因为我觉得——我觉得他对我来说很重要，然后我很在意很在意这件事情，导致于以至于——因为可能别人说有嘛没有就没有了，但是我觉得因为我付出很多，我很想成成心心的生一个孩子，我就是——为什么会这样子。他给了我希望，又突然之间没有了。然后，然后就觉得呃到底就是，就是自己嘛我觉得挺没用的，怎么会这样？

R：为什么觉得自己没用呢？

P：为什么好好的就是这样子了，肯定是因为我不好而导致他不好了，就是因为，就是有点这样子感觉。

R：你觉得是你不好导致他不好？

P：嗯，因为如果说正常的话，一般孩子的话，肯定是那个嘛，肯定是谁愿意，他说他自己胎停了什么，肯定是有某种是我带给他某种不好的地方，然后导致他心跳可能他本来心脏就不大好，那天颠了颠就把他心脏给颠坏了要不就是。

R：也就是说你也是觉得当时自己太累，或者别的一些因素，可能会影响到这个事实。

P：我就是自己身上找原因咯只有这样子，也有想过，这样。但是客观的说，我自己还是有点常识的，他们说胚胎质量不好。那么我想想，也许可能吧，但是占百分之最多占40%，其他的是另外的东西。

R：有研究显示就是早期胚胎质量不好的因素可以占到百分之七八十。

P：那为什么翻倍都好呢，我每次都是好好的。。。

R：很难说，这些东西有时候也解释不清楚。

P：因为我，我是第一次胎停，第一次人流都是第一次，之前从来——因为怀不上我之前是多囊，然后本来一直都没有，第一个怀——从来都没有说打过胎，或者从来都没有说流过产，从来都没有说意外怀孕过就是怀不上的那种。然后做试管，我做了三次人授失败了，然后这次是第三次试管又是失败了，本来还以为是能够成功的嘛1。那当中不是还做很多检查的嘛，很痛苦的，这个过程就是比如说宫腔镜，还有什么输卵管。都做过的，这肯定要做的，还有各种检查不是，然后前前后后反正你说我只能不上班了嘛，一年，反正我都不去。去中药调理，然后再那个，再自己不上班嘛，本来工作还挺好的，也差不多20万一年，那你20万工作工资不算的话，反正几十万都扔掉，就失败的话，就光试管的话就扔掉10万了。就连这次第三次，你想每次保胎，这里都住了一个多月，两个月了你费用都不好说，上次是红会医院，那次好像是生化掉了，就直接，官方的时候才19点多，就是那不是没成功了。难过是难过但也就一阵就过了，这次希望很大，全家人都很开心。然后人付出痛苦也多好多倍，是吧？打了多少针啊这里全都是那个淤青，那个是肝素嘛肝也打坏掉，我跟你说肝也坏掉嘛，我很有感觉，胃也坏掉了，胃也很难受，气胀。

R：胃难受倒不一定是针打的

P：是是是，我一天要吃36颗药，就这个不是药，就是说外加两包中药，12颗的是那个护肝片，然后人胎盘片早4晚4嘛，那个就快20颗了嘛，然后再是早3晚3的补佳乐加上那个叫什么达芙通加上叫什么阿司匹林，那个时候都吃，然后还有，还有那个叫说那个叫什么，晚上两颗的安琪坦。然后还有什么小的冲剂，忘记他说是什么，我什么不记得是那时候什么不舒服，反正一天吃好多药，搞得我就是是肾脏也不大好，整天去上厕所，两小时上一次厕所。

R：上厕所不一定是肾脏不好引起的，然后怀孕了本身就会

尿频。。。

P：然后我就跟你说那几天手上长湿疹，我知道的，我有点感觉就是因为便秘嘛那个人胎盘片吃了便秘，然后出不去坏的东西了，然后排不出来，这都长湿疹，现在好了，排出去就好了。然后哎呦反正人很难受很难受，你不知道有多痛苦。人整个本来就是那个内分泌有点那个，很难受的。所以付出的痛苦也很多倍嘛，然后你说一旦失去了，你说是不是那个？然后家里面我表妹他说什么床也送给我了，什么都准备好了，然后什么餐椅什么。这种嘛也算小事情了，家里面亲戚也知道的，感觉也挺没面子的，一下子又没有了。

R：挺没面子的？你觉得这个挺没面子的占你的因素大吗？

P：我是还好，但是我怕我父母有点挺没面子，因为为什么，因为我也不是第一次，你说两三次了都是这样。然后他们不是过来照顾我，我家宁波的，然后老家里面搓麻将什么的，他们都说哎呀他们女儿那个。。。然后农村里嘛就有的时候你老是这样，他会看看不起的，好像，她怎么又没有嘞，好像被人取笑的感觉。

R：为什么会取笑这种呢？

P：不是说别人取笑我听见了，我是我自己，就是感觉我父母会就是说他们会受点影响。我倒是无所谓，我又不在那里生活。

R：你们是听到别人在说呢，还是你们自己觉得有这种可能？

P：呃——可能久而久之会造成一种自卑的心理，可能就是说好像——

R：会有哪边耳朵上也会听到过吗？还是就你自己有这样一种想法？

P：因为我就是我自己的这边亲戚阿姨舅舅这边就是会，呃，因为我不是之前做试管的，之前不是也会去，他们带我去什么拜菩萨什么，求一求，迷信嘛，反正亲戚嘛他们也就带我去都知道的普陀山那边，然后感觉好像这次一定会成功了，这次我感觉又没成功。他们说起来好像是好像运气不大好什么的。

R：就是说之前求过一次，所以你自己也感觉自己心里有寄托。。。

P：我想这样转一下，我的脖子有点酸。（患者感觉一个姿势坐久了有点累，便更换了一个姿势）

R：你会不会太累，太累可以先去休息。我们改天再聊也没关系。

P：没事，刚才可能一直这样坐着难受。

R：相当于就是说你当时其实也相当于去拜过了，然后这次又怀孕了，就觉得这次肯定，蛮有寄托，心理面也蛮有寄托的那种。

P：是的

R：那为什么会觉得人家会觉得好像取笑你什么的，为什么

会有这种想法？

P：因为我单位你不是也是请假出来，然后又去上班，然后单位里比较现实的，就是说同事之间没有那么。但是给我感觉好像你怎么又（流产了）。。。然后言语之间稍微是有感觉一点的，能感觉到。特别同事之间有些事情，等于说比如说有利益分歧，或者是，或者是——反正就是他们有些有的时候或者有些事有点故意兮兮的这样子，我现在都想换个公司。

R：你是辞职是没有辞职是吧？

P：呃——因为老板娘很比较喜欢我的。然后我在那里上班很多年了嘛，他就说我随时都可以去，想上班就去。她作为朋友也希望我再有个孩子。

R：这几天不是刚发现不好了嘛？后来你不是又慢慢的自己有点接受了，你这几天都是怎么过来的？

P：怎么度过的？第一天，不是那天就回家了嘛，然后做梦就不敢睡，很怕很怕。

R：怕什么？

P：怕——怕什么，就是怕那个嘛，就没有孩子嘛，当时还不能接受这样子，然后眼睛闭下去，就是医生跟我说的一些话，一些情景这样子，然后闭上眼睛，然后我睡不着，第一天是这样子度过的，第二天嘛，反正第二天是那个——（停顿半晌）

R：没关系，你就自己心里，就说自己心里是怎么样一个过程？

P：第二天我正好我女儿到我这里来，正好是星期六嘛，到我这里来。星期五第二天是。然后我就又转移下注意力，然后还好，就是说还好，人也还不痛苦嘛，药还没开始吃嘛，然后第三天嘛开始吃药了，那个药反应很大。首先我觉得他那个药吃了以后，胃很难受，我就把那个药就看成是毒药。我老公给我吃这么小小的一颗，我说这药太厉害了，好像把我的孩子夺走就是打胎那种感觉。但是孩子不是本来已经就没有了嘛。然后嘛老公的意思就是说，也没什么反正就是不要怕嘛，反正家人们都是这样安慰一下，其它的，别人肯定是那个，我也都还没说。

R：你觉得你的家人在这方面就发生这个事情以后，你的家人给你的感受是什么，有没有什么改变，或者说给你感触比较深的是什么？

P：我不喜欢我父母，他们就是说比我还伤心，就是一点都不知道来安慰我一下，还要每天两个人就坐在那里脸色很差，然后叹气什么的。然后就影响我情绪。他们对我好是挺好的，照顾的很周到。但是情绪上面他们不会照顾我的，最多就说别人没有孩子也这样子过的什么。但是他们的表情或者是那个，他们自己都走不出来，他们自己都很痛苦。然后嘛我老爸嘛老是耿耿于怀，那件油漆的事情，他就说别人心里不舒服，那个是正常的。我不是说他嘛，他不是还说还要去说来

着，因为这个事情多多少少有点原因，我说不要去说了，他说现在就是说医院他说都是很怕的什么什么的，我的意思我又不是说为闹点事情出来，或者你医院赔我点钱，我又不是少点，差点这点钱。自己就是已经这样了。

R：你觉得经过这件事情以后，你觉得接下来你有什么打算呢？

P：接下来我跟我老公说是说了，不打算再去试管。因为我我想想可能跟年纪有点关系，老是不成功，也不能说是怪别的，可能各种方面都有，可能胚胎也占一点，那个年纪也占一点，或者外在原因也占一点，然后我觉得就是打算的话，还是很想要个孩子，但是自己怀是有点不怎么希望，当然正常生活如果怀上了，那就是很好的事情，因为这么多年都没有嘛没有措施都没有怀上，然后嘛就是想去领养。我跟我老公反正就是也在商量，就说没有就没有了，那怎么办，两个人生活吧。

R：就不打算再要了？

P：我是个人是不打算再要了。

R：你觉得你现在走出这件事情了吗？

P：没有。

R：那你觉得哪些方法可以让你走出这件事情，重新振作起来？

P：那我不知道，如果我知道我就自己去做啦，但是我现在也没有。

R：感觉你自己现在最期望的是什么？

P：我期望给我一个孩子，期望的话就是说或者。。呃。。

R：或者有没有在现有的条件下，比如说家里人怎么样？身边的事情会怎么样？现有的条件下，因为再给你一个孩子，这毕竟不太现实对不对？现有的条件下有哪些做法可以让你重新振作？

P：那是多赚点钱咯。然后就是孝敬父母咯。父母嘛是对自己很无私的，而且我爸妈就我一个孩子，然后他们就是挺心疼我的，就是我觉得挺对不起他们。然后照顾了半天，希望落空，他们比你还难过。然后我是想想，应该不要叫他们来这么照顾的，就是，我就觉得你哪怕请个阿姨两夫妻苦点，都不要叫他们。但是你说又不现实，又做不到，你去移植了什么，你家里人会不知道吗？知道了，他们也会照顾你，你会拒绝他们照顾吗？所以说也很矛盾。我这里头有点疼。（患者挠了挠头）

R：啊那你回去吧。我们不聊了，你有没有觉得有什么要特别要跟我说的呢？

P：特别跟你说啊，我觉得现在社会生孩子怎么这么难生，就是说有没有什么医疗科学能够发达一点，把很多东西预见性的。就我这次怀孕了两个月，他就这样没了。我就觉得医生这方面也没有什么预见性。我是来保胎，因为市中医院很

有名，我是也冲着这个名气来的。然后我觉得并不是说我觉得他哪里哪里不好，就觉得这里的医生也没什么特别没什么，就是说让我觉得你这么好这么有经验或者是这么有预见性的——并没有，并没有。每次都是也是就是说看结果，然后看看很多检查，然后跟你说怎么样。但是你怎么样了，比如说你现在你现在比如说是血指低吧或者是怎么，但是他也没有什么方法，也就这几种其他医院也在用，但是也没有告诉你，你赶紧这样或者那样，就是有预见性一点。没有，反正也就是这样。我自己付出的那么多，也不要去管了反正。你说我现在这么邋遢，以前上班的时候也还不是这样的。现在挺胖的，以前还瘦的，反正这个东西都是次要的。感觉，精神上面有点受不了。

（闲聊了一会儿，向患者列举了一些有相似经历的人的一些积极的态度。）

P：你说的这个想法我是理解的，但是因为我也好多年这样过来了嘛，我从去年过年，不就是今年的春节，我去看了一个孤寡老人（讲述一个远亲没有孩子的悲惨状况）。然后就是觉得孩子还是要生一个，然后今年又做了这个东西，然后剩下的胚胎还有，但是我也不做了因为质量也不太好。剩下的是普胚嘛，这个是囊胚，反正是蛮痛心的胚也浪费了。（讲述自己移植的过程，成功了很开心）。邵逸夫医院嘛我觉得也有点不负责任，你去移植就是进了流水线，自从你官方过了以后，他就放养的，他不给你监测血多少。他是这样的，你达到比如说你比较低的嘛他就叫你来验个。像我那次验300多，他就说你35天以后再来，这当中你出了什么问题，就是除非出血什么，你到医院，其他的他都不管你的。你不是我打个比方，我不是到这里来住院，那我还知道每天抽血什么的，别人都不是回家了嘛，那如果说本身年轻还好一点，也就成功了，有些人像我一样的，不就是什么时候用药都来不及了挽回了都。血指低两次低过以后不是都来不及了，它又不会加针什么的了，他自己又不会去加的。。。。。
